# Histone Variants and Their Post-Translational Modifications in Primary Human Fat Cells

**DOI:** 10.1371/journal.pone.0015960

**Published:** 2011-01-07

**Authors:** Åsa Jufvas, Peter Strålfors, Alexander V. Vener

**Affiliations:** Department of Clinical and Experimental Medicine, Linköping University, Linköping, Sweden; University of Tor Vergata, Italy

## Abstract

Epigenetic changes related to human disease cannot be fully addressed by studies of cells from cultures or from other mammals. We isolated human fat cells from subcutaneous abdominal fat tissue of female subjects and extracted histones from either purified nuclei or intact cells. Direct acid extraction of whole adipocytes was more efficient, yielding about 100 µg of protein with histone content of 60% –70% from 10 mL of fat cells. Differential proteolysis of the protein extracts by trypsin or ArgC-protease followed by nanoLC/MS/MS with alternating CID/ETD peptide sequencing identified 19 histone variants. Four variants were found at the protein level for the first time; particularly HIST2H4B was identified besides the only H4 isoform earlier known to be expressed in humans. Three of the found H2A potentially organize small nucleosomes in transcriptionally active chromatin, while two H2AFY variants inactivate X chromosome in female cells. HIST1H2BA and three of the identified H1 variants had earlier been described only as oocyte or testis specific histones. H2AFX and H2AFY revealed differential and variable N-terminal processing. Out of 78 histone modifications by acetylation/trimethylation, methylation, dimethylation, phosphorylation and ubiquitination, identified from six subjects, 68 were found for the first time. Only 23 of these modifications were detected in two or more subjects, while all the others were individual specific. The direct acid extraction of adipocytes allows for personal epigenetic analyses of human fat tissue, for profiling of histone modifications related to obesity, diabetes and metabolic syndrome, as well as for selection of individual medical treatments.

## Introduction

Adipose tissue has a central role in whole body energy metabolism as a dynamic store of energy in the form of triacylglycerols and as an endocrine organ that coordinates energy stores with energy intake and utilization by other tissues. In type 2 diabetes this control is perturbed by an impaired response of the fat cells to insulin [1], [2]. This is a disease of environmental effects as it is strongly linked to obesity and a sedentary lifestyle, but there is also a well recognized genetic aspect. Around 30%–70% of the risk to get type 2 diabetes has been attributed to the individual genetic background and several recent genome-wide screens have identified a number of genetic variations that carry an increased risk for the disease [3]. The studies indicated that type 2 diabetes is a very heterogeneous and polygenic disease with identified risk alleles of a very low disease penetrance and high prevalence in the general population [3]. On the other hand the intrauterine environments of either rodents or humans appear to affect the probability of later developing this disease [4]–[7] indicating the importance of environmental influences and epigenetic factors independent of the genetic predisposition. Epigenetic factors are recognized as chromatin modifications at the level of DNA-methylation, histone modifications and through RNA-interference.

Recent studies suggest that post-translational modifications (PTMs) of histones may be related to insulin resistance [6], [8]–[14]. Histones are part of a complex and dynamic gene expression control via specific combinations of their modifications [15]–[18]. The most common PTMs of histones are acetylation, methylation (mono-, di-, and tri-methylation), phosphorylation and ubiquitination, but also other modifications have been described, such as sumoylation, ADP-ribosylation, citrullination, and biotinylation [17], [19], [20]. However, epigenetic changes related to obesity and diabetes cannot be reliably studied in cell cultures or in cells from other animals. Primary human adipocytes contain a large droplet of fat which occupies more than 95% of the cell volume. The high oil content creates experimental problems to protein isolation from primary human adipocytes. To enrich histones for proteomic analysis one can take advantage of their basic properties and solubility in acid. The most common approach is to extract basic proteins from the nucleus, which means that nuclei first have to be prepared from homogenized cells [21]–[26]. Extraction of histones from whole insect cells [27] or mouse fibroblasts [28] has also been used, as well as extractions of normal and cancer tissues using HClO_4_ for isolation and characterization of the linker histone H1 variants [29], [30].

In this work we isolated histones by acid extraction of isolated nuclei and of whole primary human adipocytes. We found that a substantially higher yield of histones of all subfamilies can be achieved by direct acid extraction of the primary adipocytes, as compared with extraction from isolated nuclei. The method is suitable for direct mass spectrometric analyses of PTMs of histones. Moreover, direct homogenization of cells in acid rapidly terminates enzymatic reactions which otherwise can alter protein modifications during the cell fractionation. At the subsequent step we subjected isolated histones to differential proteolysis by trypsin or Arg-C protease and analyzed peptides by LC/MS/MS with the use of alternating CID and ETD fragmentation of peptide ions. This study identified histone isoforms characteristic for primary human adipocytes, revealed known and novel PTMs of these proteins and provided the methodology for personal epigenetic analyses and profiling of human fat tissues.

## Materials and Methods

### Isolation of human adipocytes

Samples of subcutaneous fat tissue were obtained during ordinary elective abdominal surgery on female patients at the University Hospital of Linköping. The study was approved by the Regional Ethics Committee at Linköping University; all patients obtained written information and gave their informed verbal approval before the surgery, as approved by the ethics committee considering that the procedure posed no discomfort or threat to the health of the patients. Participating female subjects were 35–76 years old, with BMI ranging from 24.4 to 32.2 kg/m^2^ and no one of them was diagnosed with type 2 diabetes. The characteristics of participating subjects are given in the Table S1. Adipocytes were isolated from human tissue samples by collagenase (type 1, Worthington, NJ, USA) digestion in Krebs-Ringer solution essentially as described before [31]. At a final concentration of about 25% cells were incubated in Krebs-Ringer solution and Dulbecco's Modified Eagle Medium (GIBCO), containing 200 nM adenosine, 25 µM HEPES, 100 u/mL penicillin and 100 µg/mL streptomycin, pH 7.4, at 37°C and 10% CO_2_ as previously described [32]. Following the overnight incubation, the cells were washed with Krebs-Ringer solution and incubated with 0.1 µM N6-phenylisopropyl adenosine and 2.5 µg/mL adenosine deaminase for 10 min before further treatment.

### Direct acid extraction of histones

Basic proteins from whole cells were extracted by acid. The cells were homogenized in 0.33 M H_2_SO_4_ with five strokes in a Dounce homogenizer, followed by 10 min centrifugation at 250 g, and removal of floating oil. The pellet was resuspended in the supernatant and incubated in a clean tube with slow rotation at 4°C for 2 h. Non-basic proteins were pelleted by centrifugation at 100 000 g for 60 min at 4°C and discarded. Trichloroacetic acid was added to the supernatant up to 25% and the samples were incubated on ice overnight. Proteins were pelleted by centrifugation at 100 000 g for 60 min at 4°C and washed 4 times with ice cold acetone with centrifugations at 16 000 g for 12 min at 4°C. Proteins were dissolved in 0.1 M HCl for protein determination by Bradford reagent (BioRad, Richmond, CA) using a bovine serum albumin standard curve. Finally the protein mixtures were dried, dissolved in 50 mM NH_4_HCO_3_ and dried again, which was repeated four times to remove residual acid.

### Extraction of histones from nuclei

To isolate nuclei, the cells were washed and homogenized in 10 mM NaH_2_PO_4_, pH 7.4, 1 mM EDTA, 0.25 M sucrose, 25 mM NaF, 1 mM Na_2_-pyrophosphate, 0.5 mM EGTA, 4 mM iodoacetate, 2 mM Na_3_VO_4_, 10 µM leupeptin, 1 µM pepstatin, 1 µM aprotinin and 0.1 mM PMSF. Nuclei were enriched and basic proteins extracted as previously described [21], [33]. Briefly the homogenized samples were layered on 0.8 M sucrose in 0.5% NP-40, 10 mM NaCl, 10 mM KCl, 1.5 mM MgCl_2_, 1 mM EDTA, 20 mM HEPES, pH 7.4 and centrifuged at 2 700 g for 15 min at 4°C. The pellet was washed with 0.25 M sucrose in 10 mM NaCl, 10 mM KCl, 1.5 mM MgCl_2_, 1 mM EDTA, 20 mM HEPES, pH 7.4 and then dissolved in 0.4 M H_2_SO_4_. After incubation in the sulfuric acid with slow rotation at 4°C for 2 h, proteins were treated the same way as described for whole cells above.

### Analysis of histone recovery after direct acid extraction

To control the recovery of histones we used immunoblotting of equal amounts of cell samples and of acid-extracted proteins, as calculated from the volumes of the starting material and of the final protein extract. The proteins were separated by SDS-PAGE (14.5% acrylamide) and transferred to a polyvinylidene difluoride blotting membrane (Immobilone-P, Millipore, MA, USA). The membrane was mounted in a SNAP i.d. system (Millipore, MA, USA), blocked with 0.5% BSA and then incubated with rabbit polyclonal anti “Histone 3 C-terminal” or “Histone H2B” antibodies (Active Motif, Carlsbad, CA, USA). Bound antibodies were detected by incubation with a secondary goat antibody conjugated to horseradish peroxidase (Santa Cruz Biotechnical, Santa Cruz, CA, USA) followed by ECL-plus (Amersham Biosciences, Little Chalfont, Bucks, UK) and chemiluminescence imaging (LAS 1000; Image Guage, Fuji, Tokyo, Japan) according to the manufacturer's instructions. The immunoblotting was repeated three times for extracts from each analyzed subject sample.

### Analysis of proteins by in-gel digestion and MS/MS

Acid-extracted proteins from whole cells or nuclei were separated on SDS-polyacrylamide gels (14% or 12% acrylamide) and stained either with silver or with Coomassie Blue R-250 (BioRad, Richmond, CA, USA). Stained gels were scanned and Photoshop Elements software (Adobe Systems, Mountain View, CA) was used for intensity analysis. Protein bands were excised from the gels, reduced with 10 mM DTT for 1 h at 56°C, alkylated with 55 mM iodoacetamide for 1 h at room temperature in darkness, and digested by trypsin for 4 h at 37°C essentially as described [34]. The extracted peptide mixtures were desalted on C_18_ reverse-phase ZipTips (Millipore) according to manufacturer's instructions. A 2 µL sample was loaded into a nanoelectrospray capillary and analyzed using API Q-STAR Pulsar I mass spectrometer (Applied Biosystems, Foster City, CA, USA). CID of selected peptide ions was performed according to manufacturer's settings with manual control of collision energy. All spectra were analyzed manually as well as using MASCOT search engine (http://www.matrixscience.com/) against the NCBInr database.

### Analysis of all acid extracted proteins by microLC/MS/MS

The acid-extracted proteins were dissolved in 25 mM NH_4_HCO_3_, pH 8.0, at a concentration of 1 µg/µL. For partial digestion with trypsin, 2.5% sequence-grade modified trypsin from Promega (Madison, WI, USA) was added and the mixture was incubated for 4 h at 20°C. Digestion was terminated by addition of 5% formic acid. To digest the proteins with ArgC-protease, 1.25% ArgC (sequencing grade endoproteinase ArgC from Clostridium histolyticum, Roche) dissolved in 25 mM NH_4_HCO_3_ with 1 mM dithiothreitol was added and incubation was performed for 1 h, 4 h or 20 h at 37°C. Digestions were terminated by addition of 5% formic acid. The peptide mixtures were dried and dissolved in 0.1% formic acid. The peptides were analyzed by microLC/MS/MS using HCTultra PTM Discovery System (Bruker Daltonics, Bremen, Germany). A 30×0.32 mm pre-column followed by a 150×0.3 mm column, both reverse phase C18 material with particle size 0.5 µm, were used for separation of the peptides at a flow rate of 7 µL/min in a gradient from 0.1% formic acid in water to 0.1% formic acid in 100% ACN. The separation gradient was distributed as follows: 0% B for the first 19 min; 0%–5% B in 19–20 min; 5% B for 20–31 min; 5%–40% B in 31–110 min; 40%–90% B in 120–130 min. An MS/MS method providing alternating CID and ETD fragmentation of the multiply charged ions was used. Peak lists were created from the raw data using Bruker Daltonics DataAnalysis 3.4 (Bruker Daltonics, Bremen, Germany). Resulting MGF files were used for searches in NCBInr (20081116) on an in-house Mascot server (version 2.2.06) (www.matrixscience.com). Search parameters allowed data mass errors up to 0.7 Da for MS; for MS/MS data 0.6 Da for CID ions and 1.3 Da for ETD ions were used. The numbers of missed cleavages allowed were 3 and the charge states were varied. Modifications allowed were N-terminal acetylation (−89.03 Da), acetylation of lysine (+42.01 Da), monomethylation of lysine and arginine (+14.02 Da), dimethylation of lysine and arginine (+28.03 Da), ubiquitination of lysine (+114.04 Da), oxidation of methionine (+15.99 Da) and phosphorylation of serine and threonine (+79.97 Da) in several different combinations. Criteria for identification of a protein were as follows: at least two peptides in the protein identified by both CID and ETD or with a MASCOT score over 45, all spectra also had to pass manual validation.

### Histone analysis by nanoLC/MS/MS

For comparative analysis of histones from six different subjects the acid-extracted proteins were dissolved in 25 mM NH_4_HCO_3_, pH 8.0, at a concentration of 1 µg/µL and digested with trypsin for 4 h at 20°C or with ArgC-protease for 1 h, 4 h or 20 h at 37°C, as described above. The digestions were terminated by addition of 5% formic acid. The peptide mixtures were dried and dissolved in 0.1% formic acid. The peptides were analyzed by nanoLC/MS/MS using HCTultra PTM Discovery System (Bruker Daltonics, Bremen, Germany). A 100 µm ×2 cm pre-column followed by a 3 µm; 75 µm ×10 cm column, both reverse phase C18 material, were used for separation of the peptides at a flow rate of 0.3 µL/min. The separation was performed in a gradient from 0.1% formic acid in water (A) to 0.1% formic acid in 100% ACN (B) as follows: 5%–20% B in the first 120 min; 20%–30% B in 120–150 min; 30%–100% B in 150–170 min; 100% B for 170–180 min. Two automated MS/MS methods were used to analyze each sample: one with alternating CID and ETD and the second performing only ETD with exclusion of 1+ and 2+ ions. The nanoLC/MS/MS analysis was implemented at least six times for samples from each of all six subjects in the study.

Peak lists were created from the raw data using Bruker Daltonics DataAnalysis 3.4 (Bruker Daltonics, Bremen, Germany) and the resulting ETD.MGF files were used for searches in a small human histone database containing all proteins (5170 entries) found when searching the NCBI database for “Homo sapiens” [porgn:__txid9606] and H1, or H2A, or H2B, or H3 or H4 on an in-house Mascot server (version 2.2.06) (www.matrixscience.com). The peptide tolerance was set to ±0.7 and the MS/MS tolerance was ±1.3. The number of missed cleavages allowed was 1 for ArgC and 3 for trypsin and the charge states of the peptides were varied between 2+, 3+, 4+, 5+ and 6+. Allowed variable modifications were: N-terminal acetylation (−89.03 Da), acetylation of lysine (+42.01 Da), monomethylation of lysine and arginine (+14.02 Da), dimethylation of lysine and arginine (+28.03 Da), ubiquitination of lysine (+114.04 Da) and phosphorylation of serine and threonine (+79.97 Da). To search all nine variable modifications in our big data sets against a database like NCBI or Swissprot an enormous data processing power is required. Thus, to overcome this problem we created the small “human histone” database. Scaffold (version Scaffold_3.0.00, Proteome Software Inc., Portland, OR) was used to validate the ETD based peptide identifications. All MASCOT-search files for one subject were loaded into scaffold and peptide identification criteria were as follow: the peptide had to be found in at least two separate LC/MS/MS experiments and pass a rigorous manual analysis validation. If peptides matched more than one member of a histone family the member with the highest coverage was reported.

## Results

### Extraction of histones from isolated nuclei and from whole cells

**Figure 1:**
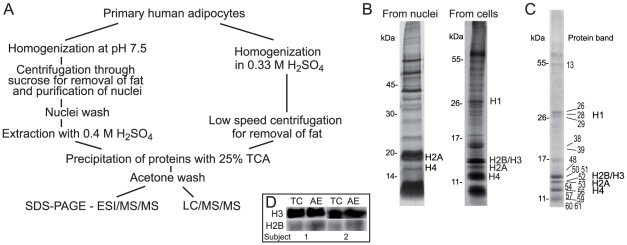
Comparison of histone preparations from nuclei and from whole cells. (A) A scheme for histone preparations from nuclei (left) and via whole cell extraction (right). (B) Silver stained SDS-PAGE gels with proteins extracted from nuclei and separated on a 14% acrylamide gel (left), and proteins extracted from whole cells and separated on a 12% acrylamide gel (right). Protein bands were cut out and in-gel digested with trypsin, the peptides were analyzed using ESI-MS/MS. Identified histones are indicated to the right. (C) Coomassie stained SDS-PAGE gel with proteins extracted from whole cells showing protein bands identified by MS/MS after in-gel digestion with trypsin. The numbers to the right indicate positions of the bands and histones as it specified in the Table 1. Molecular masses of reference proteins are indicated to the left of the gels in B and C. (D) Western blots showing efficiency of the direct acid extraction of histones from the cells by comparing immunoresponse of the samples corresponding to the equal amounts of total cell (TC) and acid extract (AE) from two different subjects. The antibodies against H3 and H2B were used, as indicated.

To analyze histones and their modifications in human adipocytes we isolated adipocytes from abdominal subcutaneous fat tissue of female subjects, obtained during elective surgery. Extraction of proteins from primary human adipocytes is challenging because of the limited amount of material and the large droplet of fat that occupies most of the cell volume. Our first approach was to isolate nuclei from adipocytes and then extract basic proteins with acid, a protocol that has extensively been used with other cell types [21]–[25] (outlined in Figure1A, left). SDS-PAGE separation of the proteins extracted by acid from isolated nuclei revealed a distinct protein pattern (Figure 1B, left lane), and analysis of the major proteins by in-gel digestion and peptide sequencing using ESI and Q-TOF analysis identified several nuclear proteins, including histones H2A and H4. However, the yield of these proteins was rather variable, depending on the particular fat tissue sample. We therefore developed a technique for histone extraction from whole cells (outlined in Figure 1A, right; and B, right lane). We tried different concentrations of sulfuric acid and found that 0.33 M H_2_SO_4_ was an optimal concentration for extraction of the histones, which yielded 2–3 times as much protein as acid extraction from nuclei. This was due to an increased yield of histones at the expense of non-histone proteins (Figure 1B). From 9–10 mL of adipocytes we extracted between 80 to 140 µg of protein. To characterize the extracted proteins we separated them by SDS-PAGE, performed in-gel digestion of 62 protein bands with trypsin and subsequent peptide sequencing using a Q-TOF instrument. Among almost 20 proteins identified by unambiguous sequencing of at least two peptides (Figure 1C; Table 1) we found histones of all the subfamilies, namely H1, H2A, H2B, H3 and H4. From the intensities of Coomassie blue stained protein bands (Figure 1C) we estimated that histones corresponded to 60%–70% of the total amount of protein in the whole cell acid extract. Such a high yield of histone proteins demonstrates a real advantage of the direct acid extraction of the adipocytes.

**Table 1:** Proteins from acid extracted whole cells identified by MS/MS after in-gel digestion.

Protein	NCBI acc. number	Band^1^	MW (kDa)	pI	Cellular location^2^	Number of peptides
Annexin A1	GI:4502101	26	38.7	6.6	Cyt, Nuc	2
Alpha 2 globin	GI:4504345	56, 57	15.3	8.7	Rbc	3
Beta globin	GI:71727161	56	16.0	7.8	Rbc	7
Chaperonin 10	GI:4008131	59	10.6	9.4	Mt	2
Crystallin, alpha B	GI:4503057	38, 39	20.0	6.8		3
Fatty Acid Binding Protein 4	GI:4557579	56	15.0	6.6	Nuc, Cyt	3
Gamma synuclein	GI:3347842	51	13.3	5.0	Cyt	2
Histone H1	GI:4885375	26, 28, 29	22.0	11.0	Nuc	6
Histone H2A	GI:28195394	52, 53, 54	14.0	10.9	Nuc	4
Histone H2B	GI:1568557	51, 52	14.0	10.3	Nuc	7
Histone H3	GI:1568559	50, 51	15.0	11.3	Nuc	3
Histone H4	GI:51315727	52,54,56,57,59	11.0	11.0	Nuc	7
PTRF	GI:42734430	54	43.5	5.5	Nuc, Mem	2
Ribosomal protein S18	GI:119624103	48	16.3	9.7	Rb	3
Ribosomal protein S19	GI:4506695	52	16.1	10.3	Rb	2
S100 Calcium binding protein A6	GI:7657532	62	10.2	5.3	Cyt, NucE	2
S100 Calcium binding protein A10	GI:4506761	58, 60, 61	11.2	6.8	Mt	6
S100 calcium binding protein A11	GI:5032057	58, 59	11.7	6.6	Cyt, Nuc	3
Vimentin	GI:62414289	13	53.7	5.1	Cyt	4

1: bands are marked in Figure 1C.

2: Mt  =  mitochondrion, Nuc  =  nucleus, Rb  =  ribosome, NucE  =  nuclear envelope, Rbc  =  red blood cells, Mem  =  membrane, Cyt  =  cytoplasm.

We also analyzed the histone recovery after the direct acid extraction of cells by SDS-PAGE and immmunoblotting using antibodies against histones H3 and H2B. There were deviations between different preparations, but in all of them we found quantitative recovery of histone H3 and the recovery of H2B was never less than 70% (Figure 1D). Further major advantages of this technique are the elimination of time-consuming steps for isolation of nuclei and the nearly instantaneous termination of all unwanted enzymatic reactions at the time point when cells are homogenized in the acid, which means that we preserve the natural PTMs of the extracted basic proteins.

### LC/MS/MS analysis of proteins extracted by acid from whole cells

The procedure of protein in-gel digestion often causes the loss of post-translationally modified peptides [35], [36]. To make a comprehensive analysis of proteins extracted from whole cells we also made digestions in solution, with either trypsin or ArgC-protease, and analyzed the generated peptides by LC/MS/MS using an ion trap performing alternating CID and ETD fragmentations of peptide ions. For identification of proteins we used MASCOT database search with the following criteria: peptides should be identified by both CID and ETD fragmentation or have a MASCOT score above 45, as well as be confirmed by manual validation of the fragmentation spectra. We identified 31 proteins with vimentin, plasma membrane protein S3-12, which is a lipid droplet associated protein, and histones as the best hits (Table S2). Vimentin and plasma membrane protein S3-12 have much higher molecular masses compared with histones; they thus produced a high number of peptides resulting in a high score. However, as shown by analyses of protein band intensities in Coomassie blue-stained gels (Figure 1C), the histone bands represented the major protein content in the whole cell acid extract. There was a significant difference between in-gel digestion followed by Q-TOF MS/MS compared to LC/MS/MS. Vimentin was for example identified by 4 peptides using in-gel digestion followed by Q-TOF MS/MS and by 27 peptides using LC/MS/MS; and plasma membrane protein S3-12, which was identified by 30 peptides with LC/MS/MS, was not found using in-gel digestion and Q-TOF MS/MS of the same sample.

### Optimization of histone analyses

Histones contain many lysines and arginines, especially in their flexible termini, which also are the most interesting parts of these proteins concerning PTMs. The frequently occurring lysines and arginines produce very small peptides when the histones are digested with trypsin. We therefore limited the digestion with trypsin to only 4 h at room temperature to create an incomplete digestion and thus formation of longer peptides, which are more suitable for characterization of modified amino acids. Moreover, we also used ArgC-protease for 1 h, 4 h or 20 h to have both complete and incomplete digestion of the proteins with this protease. Controlled proteolysis by trypsin or ArgC-protease combined with LC/MS/MS using CID and ETD fragmentation allowed us to gather sequence information from complementary peptides that differed in length and ionization states. Modifications by acetylation and trimethylation cannot be distinguished under the conditions of the study, thus we annotate all +42 Da modifications except for N-terminal acetylations in this work as acetylation/trimethylation (ac/me3).

**Figure 2:**
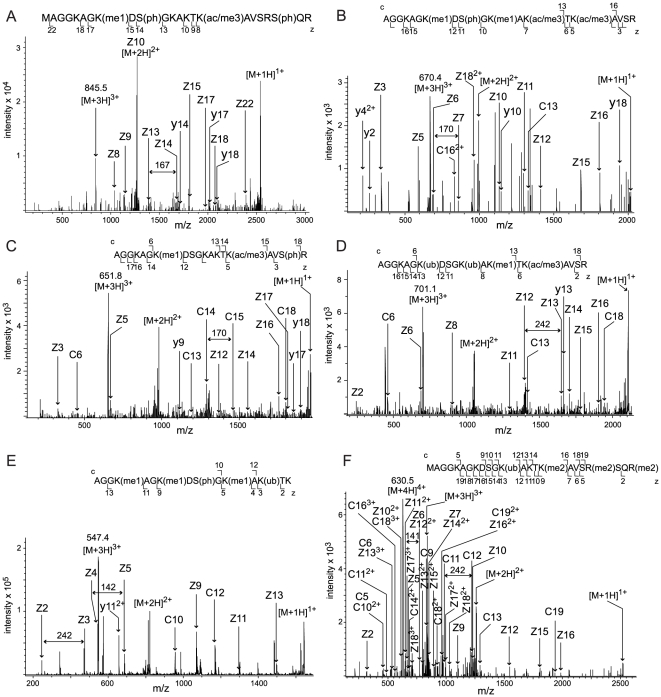
Differentially processed and modified N-termini of histone H2AFZ. ETD fragmentation spectra corresponding to six differentially processed N-terminal peptides of the variant H2AFZ (GI: 4504255). A–E show peptides identified in the histone analysis optimization process, while F presents the spectrum of the peptide identified during the following nanoLC/MS/MS analysis of six different subjects. The positions of z,y (C-terminal) and c (N-terminal) fragment ions are indicated in the spectra and in the presented peptide sequences with the lower case (ac/me3) (me1), (me2), (ph) and (ub) corresponding to modifications of the amino acids by acetylation/trimethylation, monomethylation, dimethylation, phosphorylation and ubiquitination, respectively. (A) ETD spectrum of the ion (M+3H)^3+^ at m/z 845.5 corresponding to the peptide with the amino acid positions 1–23 in H2AFZ. The peptide is modified by methylation, acetylation/trimethylation, and two phosphorylations. The mass difference of 167 between the fragment ions z13 and z14 corresponds to the phosphorylated serine at the position 10. (B) ETD spectrum of the peptide corresponding to the amino acid positions 2–20; the ion (M+3H)^3+^ at m/z 670.4. The peptide is modified by two monomethylations, two acetylations/trimethylations and a phosphorylation. The mass difference of 170 between the fragment ions z6 and z7 corresponds to the acetylated/trimethylated lysine at the position 13. (C) ETD spectrum of the peptide corresponding to the amino acid positions 2–20; the ion (M+3H)^3+^ at m/z 651.8. The peptide is modified by monomethylation, acetylation/trimethylation and phosphorylation. The mass difference of 170 between the fragment ions c14 and c15 corresponds to the acetylated/trimethylated lysine at the position 15. (D) ETD spectrum of the peptide corresponding to the amino acid positions 2–20; the ion (M+3H)^3+^ at m/z 701.1. The peptide is modified by two ubiquitinations, monomethylation and acetylation/trimethylation. The mass difference of 242 between the fragment ions z12 and z13 corresponds to the ubiquitinated lysine at the position 7. (E) ETD spectrum of the peptide corresponding to the amino acid positions 2–16; the ion (M+3H)^3+^ at m/z 547.4. The peptide is modified by three monomethylations, phosphorylation and ubiquitination. The mass difference of 242 between the fragment ions z2 and z3 corresponds to the ubiquitinated lysine and the mass difference of 142 between fragment ions z4 and z5 corresponds to the monomethylated lysine. (F) ETD spectrum of the ion (M+4H)^4+^ at m/z 630.5 corresponding to the peptide with the amino acid positions 1–23 in H2AFZ. The peptide is modified by ubiquitination and three dimethylations. The mass difference of 242 between the fragment ions c11 and c12 and the difference of 141 between the doubly charged fragment ions z11^2+^ and z12^2+^ correspond to the ubiquitinated lysine.

During optimization of the peptide analyses we first used protein digests from five subjects and found that the main challenge of the study was in the extremely high variability of the peptide patterns between the subjects. This variability originated from different histone variants as well as from diverse and multiple peptide modifications. For instance, the H2AFZ variant, which is known to substitute for the canonical H2A histones and change the chromatin structure to affect the promoter regions of specific genes [37], [38], was found in two out of five subjects by sequencing of five differentially processed peptides corresponding to the N-terminus of this histone (Figure 2). Only one of the peptides contained the N-terminal methionine (Figure 2A), while in the other four this residue had been removed (Figure 2B–E). Besides the alternative removal of the methionine, the N-terminal peptides of H2AFZ were differentially modified and contained between three to five different modifications by methylation, acetylation/trimethylation, phosphorylation and ubiquitination (Figure 2). To increase the reliability of peptide identification we substituted microLC/MS/MS by nanoLC/MS/MS and made six LC/MS/MS analyses for each acid extracted sample from six additional subjects. All identified peptide variants, modified as well as non-modified, were sequenced in at least two separate LC/MS/MS runs of histones extracted from the same person and all spectra were manually verified. These LC/MS/MS runs corresponded to samples digested by the ArgC-protease for 1 h, 4 h or 20 h, each with a technical replicate. They resulted in the identification of 18 histone isoforms carrying 78 different modifications (Tables 2, 3, 4, 5, 6). Notably, in one of the six additional subjects we found yet another N-terminal peptide variant from H2AFZ which contained the initial methionine and four other residues modified by ubiquitination and dimethylation (Figure 2F).

**Table 2:** Histone H1 peptides identified using LC/MS/MS of the acid extracted proteins digested by either trypsin or ArgC.

Protein	Peptide sequence	AA	M/Z	Z	Score/E-value
**HIST1H1E,** protein name: H1.4, GI:4885379, Acc: NP_005312, Coverage: 36%					
	(ac)-SETAPAAPAAPAPAEKTPVKKKAR	2–25	810.9	3+	61/0.00057
			608.5	4+	40/0.074
	KASGPPVSELITK	34–46	664.0	2+	65/0.0067
			443.0	3+	60/0.018
	ASGPPVSELITK	35–46	599.9	2+	67/0.0041
	KASGPPVSELITKAVAASKER	34–54	714.3	3+	100/5.8e-08
			536.1	4+	74/2.6e-05
			429.3	5+	90/5.5e-07
	SGVSLAALK	55–63	423.3	2+	71/0.0011
	SGVSLAALKKALAAAGYDVEKNNSR	55–79	1268.1	2+	80/7.3e-06
			845.8	3+	87/1.6e-06
			634.7	4+	90/9.2e-07
			507.7	5+	96/1.7e-07
	ALAAAGYDVEK	65–75	554.3	2+	80/0.00015
	GTGASGSFK	95–106	406.2	2+	35/0.52
					
**HIST1H1T,** protein name: histone H1t, GI:20544168, GI:184084, Acc: NP_005314, Acc: AAA19936, Coverage: 16%					
	ALAAAGYDVEK	69–79	554.3	2+	80/0.00015
	GTGASGSFK	102–110	406.2	2+	35/0.52
	AKKPRAT(ph)TPKTVR	153–165	512.6	3+	41/0.017
					
**H1F0,** protein name: H1.0, GI:4885371, Acc: NP_005309, Coverage: 18%					
	(ac)-TENSTSAPAAKPKR	2–15	500.0	3+	47/0.0065
	LVTTGVLKQTKGVGASGSFR	75–94	669.9	3+	70/5.1e-05
			502.4	4+	75/2e-05
					
**H1FOO,** protein name: histone H1oo, GI:28839618, Acc: AAH47943, Coverage: 8%					
	K(me1)QGGAAKDTRAQSGEAR(me2)	199–215	887.8	2+	34/0.15
			592.2	3+	46/0.012
					
**H1FNT,** protein name: testis-specific H1, GI:44953495, Acc: AAS49492, Coverage: 6%					
	AK(me2)EEAGATAADEGR	183–196	468.6	3+	29/0.17

**AA** - amino acids of the peptide numbered in the sequence of corresponding protein; **M/Z** - mass over charge ratio; **Z** – ion charge.

ac - acetylation, me1 - monomethylation, me2 - dimethylation, ph – phosphorylation.

**Table 3:** Histone H2A peptides identified using LC/MS/MS of the acid extracted proteins digested by either trypsin or ArgC.

Protein	Peptide sequence	AA	M/Z	Z	Score/E-value
**H2AFZ,** protein name: histone H2A.Z, GI:4504255, Acc: NP_002097, Coverage: 31%					
	MAGGKAGKDSGK(ub)AKTK(me2)AVS-R(me2)SQR(me2)	1–23	630.4	4+	29/0.97
	AGLQFPVGR	24–32	473.3	2+	59/0.00025
	HLQLAIR	86–92	426.0	2+	34/0.041
**H2AFX,** protein name: H2A.x, GI:4504253, Acc: NP_002096, Coverage: 36%					
	(ac)-SGRGKTGGKAR(me1)	2–12	377.6	3+	37/0.046
	GK(ac/me3)T(ph)GGK(ub)AR(me2)AKA-K(me2)SR	5–18	570.1	3+	41/0.044
	(ac)-GK(ub)TGGKARAK(me1)AK(ub)SR	5–18	567.6	3+	37/0.053
	(ac)-GK(me1)TGGKARAK(me2)AKS(ph)R	5–18	527.6	3+	20/2
	AGLQFPVGR	24–32	473.2	2+	59/0.00025
	HLQLAIR	83–89	426.0	2+	34/0.041
	VTIAQGGVLPNIQAVLLPK	101–119	966.1	2+	89/2.4e-005
**H2AFY,** protein name: core histone macro H2A.1, GI:15426458, Acc: AAH13331, Coverage: 4%					
	MSSRGGK(ac/me3)K(me2)KSTKTSR	1–15	571.0	3+	28/0.63
	SS(ph)RGGKK(me1)K(me1)STKTSR(me2)	2–15	549.3	3+	41/0.023
	SS(ph)RGGKKK(me1)STK(me1)TSR(me2)	2–15	549.4	3+	49/0.0044
	SS(ph)RGGK(me1)KKSTK(me1)TSR(me2)	2–15	549.2	3+	31/0.24
	GK(ub)K(ub)K(ub)ST(ph)K(ac/me3)TSR(me1)	6–15	533.7	3+	32/0.14
**“similar to H2AFY2”,** unnamed protein product, GI:10433885, Acc: BAB14049, Coverage: 5%					
	SGR(me1)SGKKKMS(ph)NLSR	2–15	544.5	3+	53/0.001
**HIST1H2AC,** protein name: H2A type 1-B/E, GI:24496274, Acc: AAN59965, Coverage: 29%					
	HLQLAIRNDEELNKLLGR	83–100	712.3	3+	21/7.6
			534.7	4+	40/0.069
	HLQLAIR	83–89	426.0	2+	34/0.041
	VTIAQGGVLPNIQAVLLPK	101–119	966.1	2+	89/2.4e-005
			644.5	3+	65/0.008
**histone H2A (fragment),** GI:2118981, Acc: I79341, Coverage: 47%,					
	GK(ac/me3)QGGKAKSRSSR	5–17	463.7	3+	27/0.38
	AGLQFPVGR	24–32	473.3	2+	59/0.00025

**AA** - amino acids of the peptide numbered in the sequence of corresponding protein; **M/Z** - mass over charge ratio; **Z** – ion charge.

ac - acetylation, ac/me3 - acetylation/trimethylation, me1 - monomethylation, me2 - dimethylation, ph - phosphorylation, ub – ubiquitination.

**Table 4:** Histone H2B peptides identified using LC/MS/MS of the acid extracted proteins digested by either trypsin or ArgC.

Protein	Peptide sequence	AA	M/Z	Z	Score/E-value
**HIST1H2BM,** protein name: Histone H2B type 1-M, GI:45767717, Acc: AAH67487, Coverage: 94%					
	PEPVK(ac/me3)S(ph)APVPK(me1)K(me1)GSK-K(me2)AINKAQKKDK(ub)K(ub)RK(me1)R	2–31	642.3	6+	44/0.096
	QVHPDTGISSK	48–58	584.7	2+	58/0.011
	AMGIMNSFVNDIFER	59–73	872.5	2+	109/4e-007
			582.2	3+	68/0.0059
	IAGEASR	74–80	352.2	2+	52/0.037
	LAHYNK	81–86	373.2	2+	31/10
	STITSREIQTAVR	88–100	732.0	2+	77/4.4e-06
			488.2	3+	69/4.7e-05
	EIQTAVR	94–100	409.0	2+	51/0.0026
	LLLPGELAKHAVSEGTKAVTKYTSSK	101–126	911.0	3+	49/0.014
			683.5	4+	33/0.57
			546.9	5+	104/2.6e-08
			456.2	6+	125/2.6e-10
	LLLPGELAKHAVSEGTKAVTK(ac/me3)YTS-SK(ac/me3)CASR(me2)	101–130	543.9	6+	37/0.2
**HIST1H2BA,** protein name: H2ba, GI:4254257, Acc: AAH66240, Coverage: 50%					
	QVHPDTGISSK	49–59	584.7	2+	58/0.011
	IASEAPR(me2)LAHYS(ph)K(ac/me3)R(me1)	75–88	588.8	3+	38/0.12
			442.1	4+	37/0.12
	STISSR(me1)EIQTAVR	89–101	488.4	3+	47/0.0064
	EIQTAVR	95–101	409.0	2+	51/0.0026
	LLLPGELAKHAVSEGTKAVTKYTSSK	102–127	911.0	3+	49/0.014
			683.5	4+	33/0.57
			546.9	5+	104/2.6e-08
			456.2	6+	125/2.6e-10
**HIST1H2BN,** protein name: H2bn, GI: 119623520, Acc: EAX03115, Coverage: 48%					
	QVHPDTGISSK	49–59	584.7	2+	58/0.011
	AMGIMNSFVNDIFER	60–74	872.5	2+	109/4e-007
			582.2	3+	68/0.0059
	IAGEASR	75–81	352.2	2+	52/0.037
	LAHYNK	82–87	373.2	2+	31/10
	STITSREIQTAVR	88–100	732.0	2+	77/4.4e-06
			488.2	3+	69/4.7e-05
	EIQTAVR	94–100	409.0	2+	51/0.0026
	LLLPGELAKHAVSEGTKAVTKYTSSK	101–126	911.0	3+	49/0.014
			683.5	4+	33/0.57
			546.9	5+	104/2.6e-08
			456.2	6+	125/2.6e-10
	LLLPGELAKHAVSEGTKAVTKYTSS(ph)-K(ac/me3)K(ub)R(me2)	101–128	547.1	6+	77/1.7e-05
	LLLPGELAKHAVSEGTKAVTKYTS(ph)-S(ph)K(ac/me3)K(ac/me3)R	101–128	543.8	6+	40/0.09
**HIST1H2BJ,** protein name: H2B type 1-J, GI:20336754, Acc: NP_066402, Coverage: 38%					
	QVHPDTGISSK	48–58	584.7	2+	58/0.011
	AMGIMNSFVNDIFER	59–73	872.5	2+	109/4e-007
			582.2	3+	68/0.0059
	IAGEASR	74–80	352.2	2+	52/0.037
	LAHYNK	81–86	373.2	2+	31/10
	STITSREIQTAVR	88–100	732.0	2+	77/4.4e-06
			488.2	3+	69/4.7e-05
	EIQTAVR	94–100	409.0	2+	51/0.0026
	LLLPGELAKHAVSEGTKAVTKYTSAK	101–126	679.3	4+	50/0.0083
			543.9	5+	78/1.4e-05

**AA** - amino acids of the peptide numbered in the sequence of corresponding protein; **M/Z** - mass over charge ratio; **Z** – ion charge.

ac - acetylation, ac/me3 - acetylation/trimethylation, me1 - monomethylation, me2 - dimethylation, ph - phosphorylation, ub – ubiquitination.

**Table 5:** Histone H3 peptides identified using LC/MS/MS of the acid extracted proteins digested by either trypsin or ArgC.

Protein	Peptide sequence	AA	M/Z	Z	Score/E-value
**H3F3A/H3F3B,** protein name: H3.3, GI:4504279, Acc: NP_002098, Coverage: 45%					
	KQLATK(ac/me3)AAR	19–27	515.2	2+	41/0.012
	K(me2)SAPSTGGVK(me2)KPHR	28–41	503.5	3+	60/0.00031
	YRPGTVALREIR	42–53	477.6	3+	44/00.1
	LVREIAQDFKTDLR	71–84	569.3	3+	60/0.00079
	EIAQDFKTDLR	74–84	668.5	2+	34/0.084
			466.0	3+	44/0.0083
	VTIMPKDIQLAR	118–129	463.0	3+	35/0.084
**HIST1H3I,** protein name: histone cluster 1, H3.i, GI:45219796, Acc: AAH66884, Coverage: 35%					
	KQLATK(ac/me3)AAR	19–27	515.2	2+	41/0.012
	YRPGTVALREIR	42–53	477.6	3+	44/00.1
	LVREIAQDFKTDLR	71–84	569.3	3+	60/0.00079
	EIAQDFKTDLR	74–84	668.5	2+	34/0.084
			466.0	3+	44/0.0083
	VTIMLKDIQLAR	118–129	701.5	2+	43/0.014
			468.0	3+	54/0.00096

**AA** - amino acids of the peptide numbered in the sequence of corresponding protein; **M/Z** - mass over charge ratio; **Z** – ion charge.

ac/me3 - acetylation/trimethylation, me2 – dimethylation.

**Table 6:** Histone H4 peptides identified using LC/MS/MS of the acid extracted proteins digested by either trypsin or ArgC.

Protein	Peptide sequence	AA	M/Z	Z	Score/E-value
**HIST2H4B,** GI:124504316, Acc: AAI28106, Coverage: 85%					
	VWRGKGGK(ub)GLGKGGAK(ub)R	1–17	647.8	3+	45/0.016
	VWRGKGGK(me1)GLGKGGAK(me2)R	1–17	585.6	3+	53/0.001
	GK(me2)GGK(ac/me3)GLGKGGAK(ub)R	4–17	486.0	3+	31/0.23
	GK(ub)GGKGLGK(me1)GGAK(ub)R(me2)	4–17	514.6	3+	44/0.016
	K(me2)VLRDDIQGITKPAIR	20–35	617.9	3+	75/2.1e-05
			463.8	4+	81/4.3e-06
	K(me2)VLRDNIQGITKPAIR	20–35	618.0	3+	54/0.0028
	DDIQGITKPAIR	24–35	664.2	2+	30/0.26
			443.1	3+	64/8.6e-05
	DNIQGITKPAIR	24–35	663.9	2+	59/0.00017
			443.0	3+	68/5.3e-05
	ISGLIYEETR	46–55	591.4	2+	88/2.2e-07
	GVLKVFLENVIR	56–67	694.4	2+	57/0.00055
			463.3	3+	53/0.0014
	VFLENVIR	60–67	495.5	2+	53/0.0012
	DAVTYTEHAK	68–77	567.8	2+	49/0.21
	KTVTAMDVVYALKR	79–92	532.8	3+	80/2.6e-06
	TVTAMDVVYALK	80–90	655.9	2+	65/0.0052
**HIST1H4A-L/HIST2H4A-B/HIST4H4,** GI: 4504301, Acc: NP_003529, Coverage: 74%					
	GK(me2)GGK(ac/me3)GLGKGGAK(ub)R	5–18	486.0	3+	31/0.23
	GK(ub)GGKGLGK(me1)GGAK(ub)R(me2)	5–18	514.6	3+	44/0.016
	K(me2)VLRDDIQGITKPAIR	21–36	617.9	3+	75/2.1e-05
			463.8	4+	81/4.3e-06
	K(me2)VLRDNIQGITKPAIR	21–36	618.0	3+	54/0.0028
	DDIQGITKPAIR	25–36	664.2	2+	30/0.26
			443.1	3+	64/8.6e-05
	DNIQGITKPAIR	25–36	663.9	2+	59/0.00017
			443.0	3+	68/5.3e-05
	ISGLIYEETR	47–56	591.4	2+	88/2.2e-07
	GVLKVFLENVIR	57–68	694.4	2+	57/0.00055
			463.3	3+	53/0.0014
	VFLENVIR	61–68	495.5	2+	53/0.0012
	DAVTYTEHAK	69–78	567.8	2+	49/0.21
	KTVTAMDVVYALKR	80–93	532.8	3+	80/2.6e-06
	TVTAMDVVYALK	81–91	655.9	2+	65/0.0052

**AA** - amino acids of the peptide numbered in the sequence of corresponding protein; **M/Z** - mass over charge ratio; **Z** – ion charge.

ac/me3 - acetylation/trimethylation, me1 - monomethylation, me2 - dimethylation,

ub – ubiquitination.

### Advantage of peptide fragmentation by ETD

General proteomic studies have not found ETD preference over CID peptide ion analysis [39], while the combined use of ETD and CID MS/MS have been shown to increase the total number of identified modified peptides [40], [41]. We found that using ETD was absolutely critical for the identification of heavily modified human histone variants. At the initial stage of the study when we analyzed protein digests from five subjects by microLC/MS/MS we found 64 histone peptides: 35 were detected with both CID and ETD fragmentation, 2 only with CID and 27 only with ETD. The peptides sequenced exclusively by ETD contained the vast majority out of 78 identified PTMs. Thus, for detection of the modified peptides at the following stage we focused on the MASCOT database searches using ETD data sets obtained using nanoLC/MS/MS.

**Figure 3:**
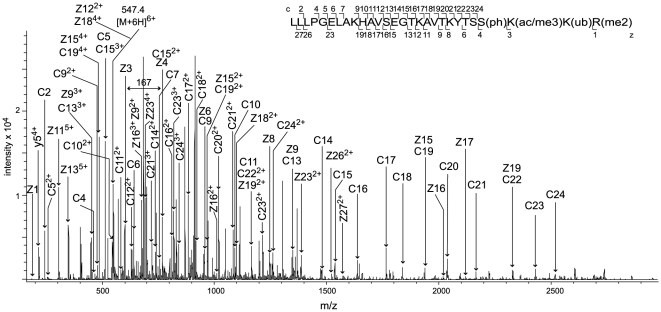
C-terminal peptide sequence of HIST1H2BN. ETD spectrum of the ion (M+6H)^6+^ at m/z 547.4 corresponding to the peptide with amino acids 101–128 in HIST1H2BN (GI: 119623520). The positions of z,y (C-terminal) and c (N-terminal) fragment ions are indicated in the spectrum and in the corresponding peptide sequence, where the lower case (ph), (ac/me3), (ub) and (me2) designate modifications of the amino acids by phosphorylation, acetylation/trimethylation, ubiquitination and dimethylation, respectively.

Figure 3 presents the ETD fragmentation spectrum of the 28 amino acid long, modified C-terminal peptide of HIST1H2BN, a histone variant not characterized at the protein level before our study but only known from the human genome sequence [42]. This spectrum with the MASCOT ions score 77 and the expect value of 1.7e-05 identifies an earlier unknown cluster of modified sites with Ser-125 phosphorylation, Lys-126 acetylation/trimethylation, Lys-127 ubiquitination and Arg-128 dimethylation. The ubiquitination of Lys-127 is of special interest because the C-terminal H2B ubiquitination is an evolutionarily conserved histone crosstalk mechanism regulating chromatin dynamics and the trans-histone H3 methylation [43]. Ubiquitination of Lys-123 in yeast histone H2B enhances nucleosome stability [44] and, notably, the residues Arg-119 and Thr-122 in the H2B C-terminal helix play a direct role in controlling this ubiquitination, as well as methylation of the Lys-4 in histone H3 [45]. Thus, the cluster of modifications around ubiquitinated Lys-127 in HIST1H2BN (Figure 3), found only due to the application of ETD, may have similar regulatory functions.

### Variants for the first time found at the protein level

We sequenced peptides from four human histone variants, namely HIST1H2BN, H2A (fragment), the protein “similar to H2AFY2” and HIST2H4B, which have previously only been found at cDNA or gene levels [42], [46], [47]. The unique quadruply modified peptide from HIST1H2BN (Figure 3) was found in cells from three persons, while the same peptide was identified from one more person with alternative modifications: Ser-124 and Ser-125 phosphorylations and Lys-126 and Lys-127 acetylations/trimethylations (Table 4, Figure S2B). The peptides from the H2A (fragment) and the protein “similar to H2AFY2” (Table 3, Figure S2A) were sequenced from the acid protein extracts obtained from the fat tissue of two and three different subjects, respectively. The isoform of histone H4, HIST2H4B, was identified from three persons with the specific N-terminal peptide differently modified either by Lys-8 and Lys-16 ubiquitination or by Lys-8 monomethylation and Lys-16 dimethylation (Table 6, Figure S4). These four histone variants found at the protein level for the first time contained 13 previously unknown PTMs (Tables 3, 4 and 6).

### Histone H1 analysis

We identified five histone H1 variants: HIST1H1E, HIST1H1T, H1F0, H1FOO and H1FNT (Table 2). H1F0 was found in five out of six persons, while HISTH1E, also known as an abundant histone protein H1.4, was found in all studied subjects. HIST1H1T, H1FOO and H1FNT were, however, found in adipocytes of one person each, probably indicating the low abundance of these histone variants, which have been considered to be oocyte and testis specific [48]–[50]. The initial methionine of HISTH1E was found to be removed and the following N-terminal serine was acetylated. N-terminal processing and acetylation of H1F0 was found as well (Table 2). H1FOO was modified by Lys-199 monomethylation and Arg-215 dimethylation, while HIST1H1T was modified by Thr-159 phosphorylation which has been mapped before [51] (Table 2, Figure S1). H1FNT was found dimethylated at the Lys-184 (Table 2, Figure S1).

### Histone H2A analysis

We identified six different variants of the histone H2A subfamily in the human adipocytes: H2AFZ, H2AFX, H2AFY, HIST1H2AC, a protein variant called histone H2A (fragment), and a protein “similar to H2AFY2” (Table 3). Strikingly, none of the variants was found in adipocytes of all six participating persons. Histone H2A (fragment) and the protein “similar to H2AFY2” have previously only been found at cDNA or gene levels [42], [46], [47]. The peptides from the H2A variants were sequenced with different PTMs (Table 3, Figure S2A). Only one of these modifications was known from previous studies of a human myeloid leukemia cell line: acetylation of the Lys-6 in H2AFX [52]. The following 32 modifications we found for the first time: in H2AFZ Lys-12 ubiquitination and dimethylations of Lys-16, Arg-20 and Arg-23; in H2AFX Lys-6 ubiquitination or monomethylation, Thr-7 and Ser-17 phosphorylations, Lys-10, Lys-16 ubiquitination, Arg-12 and Lys-14 monomethylations, and dimethylations of the Arg-12 and Lys-14, Lys-16; in H2AFY Ser-3 and Thr-10 phosphorylation, Lys-7, and Lys-12 acetylations/trimethylations, Lys 7, -8, -9, -12, and Arg-15 monomethylations, Lys-8, and Arg-15 dimethylations and Lys-7, -8 and -9 ubiquitinations; in H2A (fragment) Lys-6 acetylation/trimethylation; and in the protein similar to H2AFY2 Arg-4 monomethylation and Ser-11 phosphorylation (Table 3, Figure S2A).

**Figure 4:**
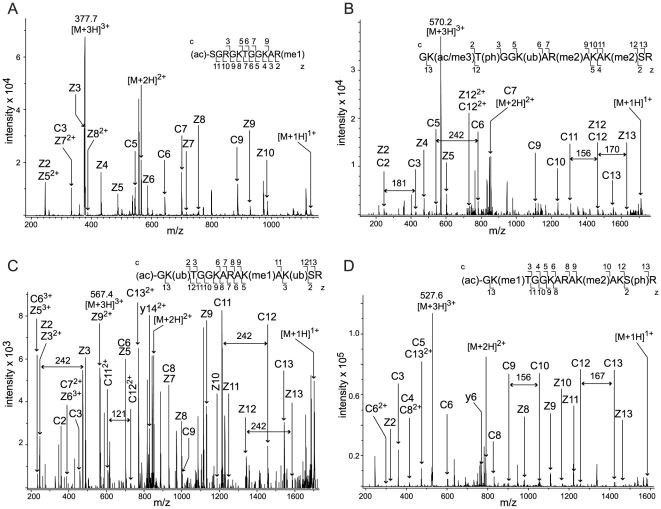
Identification of alternative processing of histone variant H2AFX. ETD fragmentation spectra corresponding to four differentially processed and modified N-termini of the isoform H2AFX (GI: 4504253). The positions of z,y (C-terminal) and c (N-terminal) fragment ions are indicated in the spectra and in the presented peptide sequences with the lower case (ac), (ac/me3) (me1), (me2), (ph) and (ub) corresponding to modifications of the amino acids by acetylation, acetylation/trimethylation, monomethylation, dimethylation, phosphorylation and ubiquitination, respectively. (A) ETD spectrum of the ion (M+3H)^3+^ at m/z 377.7 corresponding to the peptide with the amino acid positions 2–12 in H2AFX. The initial methionine has been removed, the N-terminal serine acetylated and the arginine in the peptide was monomethylated. (B) ETD spectrum of the peptide corresponding to the amino acid positions 5–18; the ion (M+3H)^3+^ at m/z 570.2. The peptide is modified by acetylation/trimethylation, phosphorylation, ubiquitination and two dimethylations. The mass difference of 181 between the fragment ions c3 and c4 corresponds to the phosphorylated threonine. The mass difference of 242 between the fragment ions c5 and c6 corresponds to the ubiquitinated lysine. The mass difference of 156 between the fragment ions c11 and c12 corresponds to the dimethylated lysine. The mass difference of 170 between the fragment ions z12 and z13 corresponds to the acetylated/trimethylated lysine. (C) ETD spectrum of the peptide corresponding to the amino acid positions 5–18; the ion (M+3H)^3+^ at m/z 567.4. The initial glycine has been acetylated after cleavage of the four N-terminal amino acids of the protein. The peptide is also modified by a monomethylation and two ubiquitinations. The mass differences of 242 between the fragment ions z2 and z3, z12 and z13 and c11 and c12, as well as the mass difference of 121 between the doubly charged fragment ions c11^2+^ and c12^2+^ correspond to the ubiquitinated lysines. (D) ETD spectrum of the peptide corresponding to the amino acid positions 5–18; the ion (M+3H)^3+^ at m/z 527.6. The N-terminal glycine is acetylated. The peptide is also modified by monomethylation, phosphorylation and dimethylation. The mass difference of 156 between the fragment ions c9 and c10 corresponds to the dimethylated lysine. The mass difference of 167 between the fragment ions c12 and c13 corresponds to the phosphorylated serine.

In addition to identification of the novel PTMs described above, we revealed a differential N-terminal processing and modification pattern for the H2AFX variant. H2AFX, also known as the histone H2A.x protein, is the abundant histone variant involved in the repair of double stranded DNA breaks [53], [54]. Removal of the initial methionine in H2AFX with the following acetylation of the N-terminal serine is expected by similarity according to uniprot (http://www.uniprot.org/uniprot/P16104). We experimentally demonstrated it here for the first time (Table 3, Figure 4A). Moreover, we also found the alternative N-terminal processing of H2AFX by removal of the first four amino acids and acetylation of Gly-5 (Table 3, Figure 4C,D). On top of this alternative N-terminal processing, each of the four sequenced peptides contained from one to four additional modifications by differential ubiquitination, monomethylation, phosphorylation, and dimethylation (Table 3, Figure 4).

### Histone H2B analysis

In the human adipocytes we identified four different variants of histone H2B: HIST1H2BM, HIST1H2BA, HIST1H2BN and HIST1H2BJ (Table 4). The variant HIST1H2BN, not found at the protein level before this study, was identified by sequencing of two differentially modified C-terminal peptides from four subjects, as described above. These peptides contained seven novel modifications by phosphorylation, acetylation, ubiquitination and dimethylation (Table 4, Figure 3, Figure S2B). The 30 amino acids long N-terminal peptide from HIST1H2BM was sequenced using ETD, which revealed eight modifications by Lys-6 acetylation/trimethylation, Ser-7 phosphorylation, Lys-12, Lys-13 and Lys-30 monomethylations, Lys-17 dimethylation and Lys-27 and Lys-28 ubiquitinations (Table 4, Figure S2B). Out of these modifications, only the acetylation of the Lys-6 has previously been described in human myeloid leukemia and airway epithelial cell lines [52], [55]. We also sequenced the C-terminal peptide from HIST1H2BM, which was modified by Lys-121 and Lys-126 acetylations/trimethylations and Arg-130 dimethylation, none of which have been published before. HIST1H2BM was sequenced with a coverage of 94% (Table 4, Figure S2B), the highest of all histones found in the study. The other variant HIST1H2BA was sequenced with a coverage of 50% (Table 4). This variant has earlier been described as a testis/sperm-specific replacement histone H2B with unknown biological function [56]. We identified two modified peptides from HIST1H2BA: one carrying Arg-81 dimethylation, Ser-86 phosphorylation, Lys-87 acetylation/trimethylation and Arg-88 monomethylation, and the other peptide monomethylated at Lys-94 (Table 4, Figure S2B). Both peptides were found in cell extracts from two different persons and none of the found modifications was previously known. Most of the unmodified H2B peptides were found in the adipocytes from all six studied subjects.

### Histone H3 analysis

We identified the isoforms HIST1H3I and H3F3A/H3F3B from the histone H3 subfamily. A peptide sequenced with Lys-24 acetylation/trimethylation was found in both isoforms, but the one carrying Lys-28 and Lys-37 dimethylations was specific for H3F3A/H3F3B, also known as the histone variant H3.3 (Table 5, Figure S3). This peptide was found in adipocytes from two of the studied subjects and so was the unmodified peptide specific for HIST1H3I. The histone H3 family is so far the most studied and all three modifications that we sequenced were previously known [57]. Notably, the N-terminal peptides from both H3 variants that we sequenced with high confidence started from the amino acid residue 19 of their translation products (Table 5). The peptides corresponding to the first 18 amino acids of histone H3 were also detected but intensity of their signals was always very low, which resulted in fragmentation spectra and sequencing quality that did not pass the identification criteria of this work. Figure S5 demonstrates an example of not accepted identification for the N-terminal peptide, the amino acids 3–18 of the histone H3, with six modified residues detected in the MASCOT search. The ion fragmentation spectrum had a low MASCOT score, a high E-value and was not considered as peptide identification, although it was found in several LC/MS/MS runs from the same subject. We suggest that the diversity of the H3 N-terminal modifications in the primary adipocytes results in numerous heterogeneous N-terminal peptide products, which decreases the probability of obtaining a high quality MS/MS fragmentation for each of them.

### Histone H4 analysis

Histone H4 is considered to be expressed in humans as only one protein [58]. The N-terminal peptide of this protein, originating from the genes *HIST1H4A-L/HIST2H4A-B/HIST4H4*, was found to be modified by previously known [59] dimethylation of Lys-21, and Lys-9 acetylation (Table 6). We also observed an earlier described deamidation of Asp-25 to Asn [60], and found the novel modifications of Lys-6 and Arg-18 by dimethylation, as well as Lys-6 and Lys-17 ubiquitination (Table 6, Figure S4). Interestingly, we sequenced two differentially modified N-terminal peptides from the earlier uncharacterized variant of histone H4, encoded by the *HIST2H4B* gene. These peptides were found in the protein extracts from three of the participating persons. The HIST2H4B variant differs from the canonical H4 only in its N-terminus where VW residues are present instead of the SG pair (Table 6). The variant specific peptide was sequenced in two differently modified versions, one with Lys-8 and Lys-16 ubiquitination and the other with Lys-8 monomethylation and Lys-16 dimethylation (Table 6, Figure S4).

## Discussion

### Histone preparation from primary human adipocytes

Differential expression of histone variants as well as their PTMs may serve as potential markers in the classification of diseases affected by chromatin abnormalities. For example, a significant decrease in the relative abundance of histone H2A variants H2AFL and H2AFA/M was observed in primary chronic lymphocytic leukemia cells as compared to normal B cells [61]. Mammalian histones show tissue-specific differences in their expression and PTMs [62], as well as pronounced differences between cell cultures and primary tissues [29]. Human subcutaneous fat tissue acquired from elective abdominal surgery could be a readily obtainable source for the analysis of histone variants and modifications related to obesity, type 2 diabetes and the metabolic syndrome. In this work we performed a comparative analysis of histone preparations from isolated nuclei and direct acid extracts of human adipocytes obtained from such surgery. We developed a straightforward and simple protocol for extraction of basic proteins from the adipocytes, and using controlled proteolytic digestions of these proteins and LC/MS/MS we revealed recovery of histone variants from all subfamilies, namely H1, H2A, H2B, H3 and H4, in amounts sufficient to make up to 100 nanoLC/MS/MS analyses. Indeed, the direct acid extraction of adipocytes yielded about 100 µg of protein with histone content of 60% –70% from about 10 mL of fat cells. Direct homogenization of the cells in acid also terminates enzymatic reactions which can change the pattern of the histone code during the alternative cell fractionation. We made six nanoLC/MS/MS analyses for each protein preparation from six individuals, and produced a snapshot of the histone variants in primary human adipocytes. This analysis uncovered person-specific diversity in histone variants and in their PTMs, and also demonstrated a requirement for additional histone separation that should complement sequencing of peptide digests to achieve comprehensive mapping of all histone isoforms. The recently described liquid chromatography technique [26], capable of identifying all of the major combinatorial histone codes present in a sample, looks extremely promising for this purpose. Chromatographic separation of individual modified histone variants could also allow relative quantitation of modified isoforms and their profiling in every individual acid extract from the fat tissue, which in turn can be used to track variants and modified isoforms related to diseases.

### Histone variants of the primary adipocytes

The specific biology of the fat cell is determined during adipogenesis via an intertwined network of transcription factors and regulators with chromatin-modifying activities, which are responsible for the establishment of the gene expression pattern of the mature adipocyte [63]. Although the transcription factor cascade controlling adipogenesis has been extensively studied, the structure of chromatin and its histone composition in fat cells remains to be determined [63]. We identified 5 variants of linker and 14 variants of core histones in primary human adipocytes. The core proteins encoded by the *HIST1H2AC, HIST1H2BA, HIST1H2BM, HIST1H2BN, HIST1H2BJ, HIST1H3I* and *HIST1H4A* replication-dependent histone genes [64] are presumably involved in bulk packaging of the nucleosome particles [58]. Among the other found isoforms H2AFX is an abundant histone variant which upon phosphorylation participates in the repair of double stranded DNA breaks [53] and in the associated chromatin rearrangements [65]. The histone variants H2A.Z (H2AFZ) and H3.3 (H3F3A/H3F3B) are implicated in specific transcription regulation. Both H2A.Z and H3.3 affect nucleosome positioning creating new positions and altering the relative occupancy of the existing nucleosome position space [66]. H2A.Z variant associates with gene promoters upon gene induction and helps in recruiting the transcriptional machinery [67].

Notably, H2A.Z nucleosomes protect only approximately 120 bp of DNA and exhibit specific sequence preferences, suggesting a novel mechanism of nucleosome organization for the H2A.Z variant [68]. The histone fragment H2A (GI:2118981), which we found for the first time at the protein level, lacks the C-terminal part of a normal H2A and had earlier been annotated as an H2A pseudogene [47]. Together with H2A.Z this histone H2A fragment could participate in the organization of specific nucleosomes associated with transcriptionally active chromatin in human adipocytes. It should be noted that we sequenced peptides specific to H2A fragment and to each of the other three newly identified variants (HIST1H2BN, the protein “similar to H2AFY2” and HIST2H4B) from several subjects.

Only one H4 protein has been found in humans earlier as the product of *HIST1H4A-L/HIST2H4A-B/HIST4H4* genes [58], [64]. We found the additional variant HIST2H4B, which in mature form differs from the canonical H4 in the first two amino acid residues. The function of this additional H4 variant in primary human adipocytes remains to be established. The protein “similar to H2AFY2” is analogous to macroH2A.2 variant histones, which replace conventional H2A in a subset of nucleosomes where they repress transcription. We also found H2AFY, which is a macroH2A1 enriched on the inactive X chromosome in female mammalian cells, where it functions to maintain gene silencing [69]. This may reflect the fact that all preparations were made from the fat tissue of female subjects. Accordingly, H2A.Z is known to incorporate into the promoter regions of estrogen receptor target genes upon gene induction [70] and to help in recruiting the transcriptional machinery [67]. H2A.Z-containing nucleosomes exhibit altered linker histone binding [66]. Two identified linker histones are encoded by *HIST1H1E* and *HIST1H1T* replication-dependent histone genes [64]. However, the finding of HIST1H1T, H1FOO and H1FNT in the adipocytes was surprising, because these variants are considered to be oocyte or testis specific [48]–[50]. Additionally, we sequenced specific peptides from the HIST1H2BA variant that also have been described as a testis/sperm-specific replacement histone H2B with unknown biological function [56]. Thus, a specific subset of the H2A, H2B and linker histone variants identified in our study may contribute to a distinct chromatin structure interacting with transcription factors and chromatin-modifying regulators responsible for the establishment of the gene expression pattern [63] that drives the specialized biology of adipocyte cells.

### Diversity of histone modifications in the adipocytes

Analyzing cell extracts from six subjects we identified 78 PTMs in the histone proteins with 68 modifications being found for the first time. The vast majority of the modified peptides were found by sequencing of the multiply charged ions using ETD (Tables 2, 3, 4, 5, 6), while CID analyses revealed a limited number of PTMs. We also identified alternative N-terminal processing of H2AFY, as well as complex differential N-terminal processing of H2AFX. Heterogeneity of N-terminal processing was detected in the samples from different subjects, as well as in the acid extract from adipocytes of the same person. It is well known that a majority of the functionally important modifications of the core histones are localized in their N-terminal tails [19]. We identified the N-terminal regions of mature histones H1, H2A, H2B and H4, while were not able to find the very N-terminal peptide corresponding to histone H3. All the N-terminal peptides from both H3 variants that we sequenced from six different subjects were starting from the amino acid residue 19 of the corresponding initial translation product. We suggest that despite the successful application of ETD for sequencing of several peptides with multiple modifications, the N-terminus of H3 in the primary human adipocytes is so heavily and differentially modified that its heterogeneous products did not pass the MS/MS quality criteria used for identification in this study.

We found methylation of H3.3 at Lys-28, which was earlier linked to gene repression and shown to be enriched in inactive X chromosome chromatin [62]. Similarly, the H4 Lys-21 methylation, which we identified in several subjects, maintains silent chromatin. Drosophila female X chromosome has significantly increased levels of Lys-21 methylation in H4 compared to that of males [71]. These modifications in H3.3 and H4 from the fat of female subjects are in line with identification of H2AFY and the protein “similar to H2AFY2”, the variant histones that most probably function to maintain gene silencing and inactive X chromosome in female mammalian cells [69]. Additionally, the methylation at Lys-28 in H3 is known to be involved in establishment of the gene expression pattern of mature adipocytes [63]. However, we did not find many identical histone modifications in adipocytes from six different individuals. Only 23 out of 78 histone modifications were found in the samples from two or more subjects. Moreover, the diversity of histone modifications increased with the number of subjects analyzed. For instance, Figure 2 demonstrates sequences of six differentially modified peptides corresponding to the N-terminus of H2AFZ. Only one of these peptides (Figure 2F) was found during the systematic analyses of the cell extracts from six subjects, which identified 78 PTMs. The other five peptides differentially processed by removal of the N-terminal methionine, methylation, acetylation/trimethylation, phosphorylation and ubiquitination (Figure 2A–E) were found in the cell extracts from five other subjects during the optimization stage of our proteomic study. Thus, analyses of more subjects resulted in detection of eleven additional modifications in the N-terminal tail of H2AFZ. These findings demonstrate that histone modifications in primary human adipocytes are extremely heterogeneous and person specific.

The adipose tissue and its major cell type the adipocyte is a prime mover in the coordination of energy storage with energy utilization and food intake. Metabolic diseases emanating from the adipose tissue and the adipocytes include obesity, lipodystrophy, type 2 diabetes, hypertension, cardiovascular disease and dyslipidemia. These are major diseases that are rapidly increasing in incidence worldwide. We are still far from understanding the molecular underpinnings, but this work makes it possible to study epigenetic regulation of the histones in adipocytes in relation to diseases. Subcutaneous adipose tissue is comparatively accessible for biopsies. Correlation studies of histone variants and their modifications in fat cells with disease characteristics could therefore identify disease-specific histone marks. The direct acid extraction protocol, which we have developed in this work, may, moreover, allow the conduction of personal epigenetic analyses of the adipose tissue for profiling of histone modifications related to obesity, type 2 diabetes and the metabolic syndrome; as well as for prescription of individual medical treatments.

## Supporting Information

Figure S1 **Peptide identification views from MASCOT data analyses of modified peptides from histone H1 sequenced by electron transfer dissociation of their ions.** The spectra, corresponding lists of singly and doubly charged fragment ions and positions of the modified residues identified in the MASCOT search are shown. Additional manual validation of fragment ions with the charge states higher then 2+ had been done for all spectra (not shown) to accomplish and confirm correct peptide sequencing.
(DOC)

Figure S2 **Peptide identification views from MASCOT data analyses of modified peptides from histone H2A (Figure S2A) and histone H2B (Figure S2B) sequenced by electron transfer dissociation of their ions.** The spectra, corresponding lists of singly and doubly charged fragment ions and positions of the modified residues identified in the MASCOT search are shown. Additional manual validation of fragment ions with the charge states higher then 2+ had been done for all spectra (not shown) to accomplish and confirm correct peptide sequencing.
(DOC)

Figure S3 **Peptide identification views from MASCOT data analyses of modified peptides from histone H3 sequenced by electron transfer dissociation of their ions.** The spectra, corresponding lists of singly and doubly charged fragment ions and positions of the modified residues identified in the MASCOT search are shown.
(DOC)

Figure S4 **Peptide identification views from MASCOT data analyses of modified peptides from histone H4 sequenced by electron transfer dissociation of their ions.** The spectra, corresponding lists of singly and doubly charged fragment ions and positions of the modified residues identified in the MASCOT search are shown. Additional manual validation of fragment ions with the charge states higher then 2+ had been done for all spectra (not shown) to accomplish and confirm correct peptide sequencing.
(DOC)

Figure S5 **Example of not accepted peptide identification from MASCOT data analysis of the modified N-terminal peptide (amino acids 3-18) from histone H3.** The spectrum, corresponding list of singly and doubly charged fragment ions and positions of six modified residues identified in the MASCOT search are shown. The ion fragmentation spectrum has a low MASCOT score, a high E-value and not considered as the peptide identification, although it was found in several LC/MS/MS runs from the same subject.
(DOC)

Table S1 **Characteristics of participating subjects.**
(PDF)

Table S2 **Proteins from acid extracted whole cells identified by the LC/MS/MS.**
(PDF)
