# Supplementary material for: Histone Variants and Their Post-Translational Modifications in Primary Human Fat Cells
Source: PLoS One. 2011 Jan 7;6(1):e15960. doi: 10.1371/journal.pone.0015960 (PMC3017551; doi:10.1371/journal.pone.0015960)
Supplement: Figure S1 — Peptide identification views from MASCOT data analyses of modified peptides from histone H1 sequenced by electron transfer dissociation of their ions. The spectra, corresponding lists of singly and doubly charged fragment ions and positions of the modified residues identified in the MASCOT search are shown. Additional manual validation of fragment ions with the charge states higher then 2+ had been done for all spectra (not shown) to accomplish and confirm correct peptide sequencing. (DOC) [file pone.0015960.s001.doc]

**Figure S1. Peptide identification views from MASCOT data analyses of modified peptides from histone H1 sequenced by electron transfer dissociation of their ions.**

**HIST1H1E**, GI:4885379

MS/MS Fragmentation of **SETAPAAPAAPAPAEKTPVKKKAR, 811.2^3+^ and 608.5^4+^**


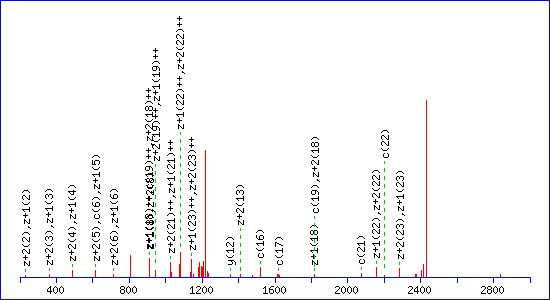


**N-term :** Acetyl (N-term)

**Ions Score:** 61 **Expect:** 0.00064

**Matches (Red):** 37/184 fragment ions using 31 most intense peaks

| **#** | **c** | **c^++^** | **Seq.** | **y** | **y^++^** | **z+1** | **z+1^++^** | **z+2** | **z+2^++^** | **#** |
| --- | --- | --- | --- | --- | --- | --- | --- | --- | --- | --- |
| **1** | 147.0764 | 74.0418 | **S** |  |  |  |  |  |  | **24** |
| **2** | 276.1190 | 138.5631 | **E** | 2300.3085 | 1150.6579 | **2284.2898** | **1142.6485** | **2285.2976** | **1143.1524** | **23** |
| **3** | 377.1667 | 189.0870 | **T** | 2171.2659 | 1086.1366 | **2155.2472** | **1078.1272** | **2156.2550** | **1078.6311** | **22** |
| **4** | 448.2038 | 224.6055 | **A** | 2070.2182 | 1035.6127 | 2054.1995 | **1027.6034** | 2055.2073 | **1028.1073** | **21** |
| **5** | 545.2566 | 273.1319 | **P** | 1999.1811 | 1000.0942 | 1983.1624 | 992.0848 | 1984.1702 | 992.5887 | **20** |
| **6** | **616.2937** | 308.6505 | **A** | 1902.1283 | 951.5678 | 1886.1096 | **943.5584** | 1887.1174 | **944.0624** | **19** |
| **7** | 687.3308 | 344.1690 | **A** | 1831.0912 | 916.0493 | **1815.0725** | **908.0399** | **1816.0803** | **908.5438** | **18** |
| **8** | 784.3836 | 392.6954 | **P** | 1760.0541 | 880.5307 | 1744.0354 | 872.5213 | 1745.0432 | 873.0252 | **17** |
| **9** | 855.4207 | 428.2140 | **A** | 1663.0014 | 832.0043 | 1646.9826 | 823.9950 | 1647.9905 | 824.4989 | **16** |
| **10** | 926.4578 | 463.7325 | **A** | 1591.9642 | 796.4858 | 1575.9455 | 788.4764 | 1576.9533 | 788.9803 | **15** |
| **11** | 1023.5106 | 512.2589 | **P** | 1520.9271 | 760.9672 | 1504.9084 | 752.9578 | 1505.9162 | 753.4618 | **14** |
| **12** | 1094.5477 | 547.7775 | **A** | 1423.8744 | 712.4408 | 1407.8556 | 704.4315 | **1408.8635** | 704.9354 | **13** |
| **13** | 1191.6004 | 596.3039 | **P** | 1352.8372 | 676.9223 | 1336.8185 | 668.9129 | 1337.8263 | 669.4168 | **12** |
| **14** | 1262.6375 | 631.8224 | **A** | 1255.7845 | 628.3959 | 1239.7658 | 620.3865 | 1240.7736 | 620.8904 | **11** |
| **15** | 1391.6801 | 696.3437 | **E** | 1184.7474 | 592.8773 | 1168.7286 | 584.8680 | 1169.7365 | 585.3719 | **10** |
| **16** | **1519.7751** | 760.3912 | **K** | 1055.7048 | 528.3560 | 1039.6861 | 520.3467 | 1040.6939 | 520.8506 | **9** |
| **17** | **1620.8228** | 810.9150 | **T** | 927.6098 | 464.3085 | **911.5911** | 456.2992 | **912.5989** | 456.8031 | **8** |
| **18** | 1717.8755 | 859.4414 | **P** | 826.5621 | 413.7847 | 810.5434 | 405.7753 | 811.5512 | 406.2793 | **7** |
| **19** | **1816.9440** | 908.9756 | **V** | 729.5094 | 365.2583 | **713.4906** | 357.2490 | **714.4985** | 357.7529 | **6** |
| **20** | 1945.0389 | 973.0231 | **K** | 630.4410 | 315.7241 | **614.4222** | 307.7148 | **615.4301** | 308.2187 | **5** |
| **21** | **2073.1339** | 1037.0706 | **K** | 502.3460 | 251.6766 | **486.3273** | 243.6673 | **487.3351** | 244.1712 | **4** |
| **22** | **2201.2288** | 1101.1181 | **K** | 374.2510 | 187.6292 | **358.2323** | 179.6198 | **359.2401** | 180.1237 | **3** |
| **23** | 2272.2660 | 1136.6366 | **A** | 246.1561 | 123.5817 | **230.1373** | 115.5723 | **231.1452** | 116.0762 | **2** |
| **24** |  |  | **R** | 175.1190 | 88.0631 | 159.1002 | 80.0538 | 160.1081 | 80.5577 | **1** |


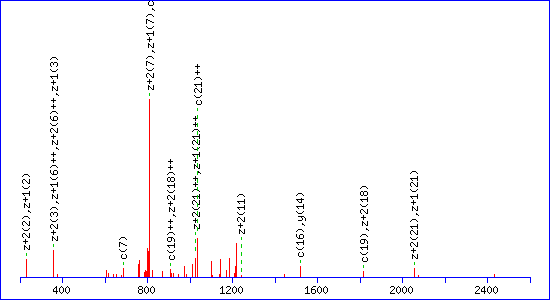


**N-term :** Acetyl (N-term)

**Ions Score:** 40 **Expect:** 0.074

**Matches (Red):** 22/184 fragment ions using 13 most intense peaks

| **#** | **c** | **c^++^** | **Seq.** | **y** | **y^++^** | **z+1** | **z+1^++^** | **z+2** | **z+2^++^** | **#** |
| --- | --- | --- | --- | --- | --- | --- | --- | --- | --- | --- |
| **1** | 147.0764 | 74.0418 | **S** |  |  |  |  |  |  | **24** |
| **2** | 276.1190 | 138.5631 | **E** | 2300.3085 | 1150.6579 | 2284.2898 | 1142.6485 | 2285.2976 | 1143.1524 | **23** |
| **3** | 377.1667 | 189.0870 | **T** | 2171.2659 | 1086.1366 | 2155.2472 | 1078.1272 | 2156.2550 | 1078.6311 | **22** |
| **4** | 448.2038 | 224.6055 | **A** | 2070.2182 | 1035.6127 | **2054.1995** | **1027.6034** | **2055.2073** | **1028.1073** | **21** |
| **5** | 545.2566 | 273.1319 | **P** | 1999.1811 | 1000.0942 | 1983.1624 | 992.0848 | 1984.1702 | 992.5887 | **20** |
| **6** | 616.2937 | 308.6505 | **A** | 1902.1283 | 951.5678 | 1886.1096 | 943.5584 | 1887.1174 | 944.0624 | **19** |
| **7** | **687.3308** | 344.1690 | **A** | 1831.0912 | 916.0493 | 1815.0725 | 908.0399 | **1816.0803** | **908.5438** | **18** |
| **8** | 784.3836 | 392.6954 | **P** | 1760.0541 | 880.5307 | 1744.0354 | 872.5213 | 1745.0432 | 873.0252 | **17** |
| **9** | 855.4207 | 428.2140 | **A** | 1663.0014 | 832.0043 | 1646.9826 | 823.9950 | 1647.9905 | 824.4989 | **16** |
| **10** | 926.4578 | 463.7325 | **A** | 1591.9642 | 796.4858 | 1575.9455 | 788.4764 | 1576.9533 | 788.9803 | **15** |
| **11** | 1023.5106 | 512.2589 | **P** | 1520.9271 | 760.9672 | 1504.9084 | 752.9578 | 1505.9162 | 753.4618 | **14** |
| **12** | 1094.5477 | 547.7775 | **A** | 1423.8744 | 712.4408 | 1407.8556 | 704.4315 | 1408.8635 | 704.9354 | **13** |
| **13** | 1191.6004 | 596.3039 | **P** | 1352.8372 | 676.9223 | 1336.8185 | 668.9129 | 1337.8263 | 669.4168 | **12** |
| **14** | 1262.6375 | 631.8224 | **A** | 1255.7845 | 628.3959 | 1239.7658 | 620.3865 | **1240.7736** | 620.8904 | **11** |
| **15** | 1391.6801 | 696.3437 | **E** | 1184.7474 | 592.8773 | 1168.7286 | 584.8680 | 1169.7365 | 585.3719 | **10** |
| **16** | **1519.7751** | 760.3912 | **K** | 1055.7048 | 528.3560 | 1039.6861 | 520.3467 | 1040.6939 | 520.8506 | **9** |
| **17** | 1620.8228 | **810.9150** | **T** | 927.6098 | 464.3085 | 911.5911 | 456.2992 | 912.5989 | 456.8031 | **8** |
| **18** | 1717.8755 | 859.4414 | **P** | 826.5621 | 413.7847 | **810.5434** | 405.7753 | **811.5512** | 406.2793 | **7** |
| **19** | **1816.9440** | **908.9756** | **V** | 729.5094 | 365.2583 | 713.4906 | **357.2490** | 714.4985 | **357.7529** | **6** |
| **20** | 1945.0389 | 973.0231 | **K** | 630.4410 | 315.7241 | 614.4222 | 307.7148 | 615.4301 | 308.2187 | **5** |
| **21** | 2073.1339 | **1037.0706** | **K** | 502.3460 | 251.6766 | 486.3273 | 243.6673 | 487.3351 | 244.1712 | **4** |
| **22** | 2201.2288 | 1101.1181 | **K** | 374.2510 | 187.6292 | **358.2323** | 179.6198 | **359.2401** | 180.1237 | **3** |
| **23** | 2272.2660 | 1136.6366 | **A** | 246.1561 | 123.5817 | **230.1373** | 115.5723 | **231.1452** | 116.0762 | **2** |
| **24** |  |  | **R** | 175.1190 | 88.0631 | 159.1002 | 80.0538 | 160.1081 | 80.5577 | **1** |

**H1FOO,** GI:28839618

MS/MS Fragmentation of **KQGGAAKDTRAQSGEAR, 592.2^3+^ and 887.8^2+^**


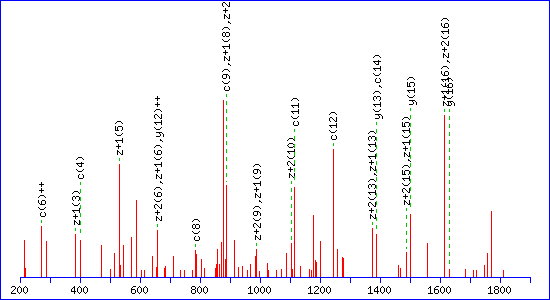


**K1 :** Methyl (K)

**R17 :** Dimethyl (R)

**Ions Score:** 46 **Expect:** 0.012

**Matches (Red):** 26/128 fragment ions using 32 most intense

| **#** | **c** | **c^++^** | **Seq.** | **y** | **y^++^** | **z+1** | **z+1^++^** | **z+2** | **z+2^++^** | **#** |
| --- | --- | --- | --- | --- | --- | --- | --- | --- | --- | --- |
| **1** | 160.1444 | 80.5759 | **K** |  |  |  |  |  |  | **17** |
| **2** | 288.2030 | 144.6051 | **Q** | **1630.8256** | 815.9164 | **1614.8068** | 807.9071 | **1615.8147** | 808.4110 | **16** |
| **3** | 345.2245 | 173.1159 | **G** | **1502.7670** | 751.8871 | **1486.7483** | 743.8778 | **1487.7561** | 744.3817 | **15** |
| **4** | **402.2459** | 201.6266 | **G** | 1445.7455 | 723.3764 | 1429.7268 | 715.3670 | 1430.7346 | 715.8710 | **14** |
| **5** | 473.2831 | 237.1452 | **A** | **1388.7241** | 694.8657 | **1372.7053** | 686.8563 | **1373.7132** | 687.3602 | **13** |
| **6** | 544.3202 | 272.6637 | **A** | 1317.6870 | 659.3471 | 1301.6682 | 651.3378 | 1302.6761 | 651.8417 | **12** |
| **7** | 672.4151 | 336.7112 | **K** | 1246.6498 | 623.8286 | 1230.6311 | 615.8192 | 1231.6389 | 616.3231 | **11** |
| **8** | **787.4421** | 394.2247 | **D** | 1118.5549 | 559.7811 | 1102.5362 | 551.7717 | **1103.5440** | 552.2756 | **10** |
| **9** | **888.4898** | 444.7485 | **T** | 1003.5279 | 502.2676 | **987.5092** | 494.2582 | **988.5170** | 494.7622 | **9** |
| **10** | 1044.5909 | 522.7991 | **R** | 902.4803 | 451.7438 | **886.4615** | 443.7344 | **887.4694** | 444.2383 | **8** |
| **11** | **1115.6280** | 558.3176 | **A** | 746.3791 | 373.6932 | 730.3604 | 365.6838 | 731.3682 | 366.1878 | **7** |
| **12** | **1243.6866** | 622.3469 | **Q** | 675.3420 | 338.1747 | **659.3233** | 330.1653 | **660.3311** | 330.6692 | **6** |
| **13** | 1330.7186 | 665.8629 | **S** | 547.2835 | 274.1454 | **531.2647** | 266.1360 | 532.2726 | 266.6399 | **5** |
| **14** | **1387.7401** | 694.3737 | **G** | 460.2514 | 230.6293 | 444.2327 | 222.6200 | 445.2405 | 223.1239 | **4** |
| **15** | 1516.7826 | 758.8950 | **E** | 403.2300 | 202.1186 | **387.2112** | 194.1093 | 388.2191 | 194.6132 | **3** |
| **16** | 1587.8198 | 794.4135 | **A** | 274.1874 | 137.5973 | 258.1686 | 129.5880 | 259.1765 | 130.0919 | **2** |
| **17** |  |  | **R** | 203.1503 | 102.0788 | 187.1315 | 94.0694 | 188.1394 | 94.5733 | **1** |


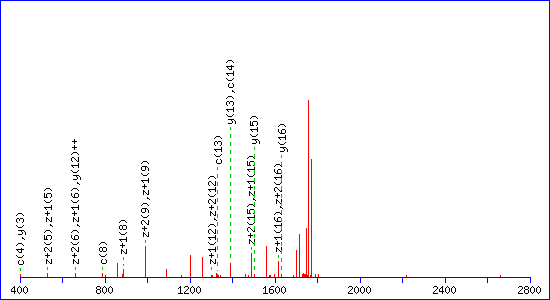


**K1 :** Methyl (K)

**R17 :** Dimethyl (R)

**Ions Score:** 34 **Expect:** 0.15

**Matches (Red):** 22/128 fragment ions using 32 most intense peaks

| **#** | **c** | **c^++^** | **Seq.** | **y** | **y^++^** | **z+1** | **z+1^++^** | **z+2** | **z+2^++^** | **#** |
| --- | --- | --- | --- | --- | --- | --- | --- | --- | --- | --- |
| **1** | 160.1444 | 80.5759 | **K** |  |  |  |  |  |  | **17** |
| **2** | 288.2030 | 144.6051 | **Q** | **1630.8256** | 815.9164 | **1614.8068** | 807.9071 | **1615.8147** | 808.4110 | **16** |
| **3** | 345.2245 | 173.1159 | **G** | **1502.7670** | 751.8871 | **1486.7483** | 743.8778 | **1487.7561** | 744.3817 | **15** |
| **4** | **402.2459** | 201.6266 | **G** | 1445.7455 | 723.3764 | 1429.7268 | 715.3670 | 1430.7346 | 715.8710 | **14** |
| **5** | 473.2831 | 237.1452 | **A** | **1388.7241** | 694.8657 | 1372.7053 | 686.8563 | 1373.7132 | 687.3602 | **13** |
| **6** | 544.3202 | 272.6637 | **A** | 1317.6870 | 659.3471 | **1301.6682** | 651.3378 | **1302.6761** | 651.8417 | **12** |
| **7** | 672.4151 | 336.7112 | **K** | 1246.6498 | 623.8286 | 1230.6311 | 615.8192 | 1231.6389 | 616.3231 | **11** |
| **8** | **787.4421** | 394.2247 | **D** | 1118.5549 | 559.7811 | 1102.5362 | 551.7717 | 1103.5440 | 552.2756 | **10** |
| **9** | 888.4898 | 444.7485 | **T** | 1003.5279 | 502.2676 | **987.5092** | 494.2582 | **988.5170** | 494.7622 | **9** |
| **10** | 1044.5909 | 522.7991 | **R** | 902.4803 | 451.7438 | **886.4615** | 443.7344 | 887.4694 | 444.2383 | **8** |
| **11** | 1115.6280 | 558.3176 | **A** | 746.3791 | 373.6932 | 730.3604 | 365.6838 | 731.3682 | 366.1878 | **7** |
| **12** | 1243.6866 | 622.3469 | **Q** | 675.3420 | 338.1747 | **659.3233** | 330.1653 | **660.3311** | 330.6692 | **6** |
| **13** | **1330.7186** | 665.8629 | **S** | 547.2835 | 274.1454 | **531.2647** | 266.1360 | **532.2726** | 266.6399 | **5** |
| **14** | **1387.7401** | 694.3737 | **G** | 460.2514 | 230.6293 | 444.2327 | 222.6200 | 445.2405 | 223.1239 | **4** |
| **15** | 1516.7826 | 758.8950 | **E** | **403.2300** | 202.1186 | 387.2112 | 194.1093 | 388.2191 | 194.6132 | **3** |
| **16** | 1587.8198 | 794.4135 | **A** | 274.1874 | 137.5973 | 258.1686 | 129.5880 | 259.1765 | 130.0919 | **2** |
| **17** |  |  | **R** | 203.1503 | 102.0788 | 187.1315 | 94.0694 | 188.1394 | 94.5733 | **1** |

**HIST1H1T,** GI:20544168, GI:184084

MS/MS Fragmentation of **AKKPRATTPKTVR**, **512.6^3+^**


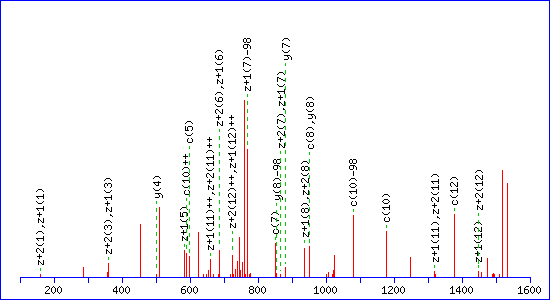


**T7 :** Phospho (ST), with neutral losses 0.0000(shown in table), 97.9769

**Ions Score:** 41 **Expect:** 0.017

**Matches (Red):** 31/144 fragment ions using 42 most intense peaks

| **#** | **c** | **c^++^** | **Seq.** | **y** | **y^++^** | **z+1** | **z+1^++^** | **z+2** | **z+2^++^** | **#** |
| --- | --- | --- | --- | --- | --- | --- | --- | --- | --- | --- |
| **1** | 89.0709 | 45.0391 | **A** |  |  |  |  |  |  | **13** |
| **2** | 217.1659 | 109.0866 | **K** | 1462.8254 | 731.9163 | **1446.8067** | 723.9070 | **1447.8145** | 724.4109 | **12** |
| **3** | 345.2609 | 173.1341 | **K** | 1334.7304 | 667.8688 | **1318.7117** | 659.8595 | **1319.7195** | 660.3634 | **11** |
| **4** | 442.3136 | 221.6605 | **P** | 1206.6354 | 603.8214 | 1190.6167 | 595.8120 | 1191.6246 | 596.3159 | **10** |
| **5** | **598.4147** | 299.7110 | **R** | 1109.5827 | 555.2950 | 1093.5640 | 547.2856 | 1094.5718 | 547.7895 | **9** |
| **6** | 669.4519 | 335.2296 | **A** | 953.4816 | 477.2444 | **937.4629** | 469.2351 | **938.4707** | 469.7390 | **8** |
| **7** | **850.4659** | 425.7366 | **T** | 882.4445 | 441.7259 | **866.4257** | 433.7165 | **867.4336** | 434.2204 | **7** |
| **8** | **951.5135** | 476.2604 | **T** | 701.4305 | 351.2189 | **685.4117** | 343.2095 | **686.4196** | 343.7134 | **6** |
| **9** | 1048.5663 | 524.7868 | **P** | 600.3828 | 300.6950 | **584.3640** | 292.6857 | 585.3719 | 293.1896 | **5** |
| **10** | **1176.6613** | 588.8343 | **K** | 503.3300 | 252.1686 | 487.3113 | 244.1593 | 488.3191 | 244.6632 | **4** |
| **11** | 1277.7089 | 639.3581 | **T** | 375.2350 | 188.1212 | **359.2163** | 180.1118 | **360.2241** | 180.6157 | **3** |
| **12** | **1376.7774** | 688.8923 | **V** | 274.1874 | 137.5973 | 258.1686 | 129.5880 | 259.1765 | 130.0919 | **2** |
| **13** |  |  | **R** | 175.1190 | 88.0631 | **159.1002** | 80.0538 | **160.1081** | 80.5577 | **1** |

**H1F0,** GI:4885371

MS/MS Fragmentation of **TENSTSAPAAKPKR, 501.1^3+^**


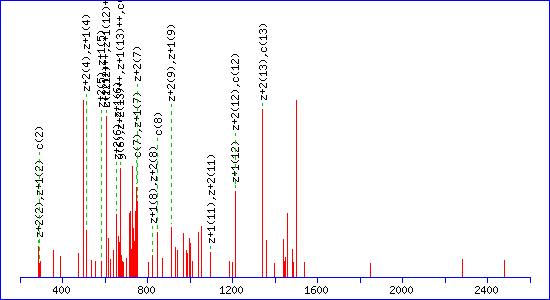


**N-term :** Acetyl (N-term)

**Ions Score:** 47 **Expect:** 0.0065

**Matches (Red):** 31/104 fragment ions using 42 most intense peaks

| **#** | **c** | **c^++^** | **Seq.** | **y** | **y^++^** | **z+1** | **z+1^++^** | **z+2** | **z+2^++^** | **#** |
| --- | --- | --- | --- | --- | --- | --- | --- | --- | --- | --- |
| **1** | 161.0921 | 81.0497 | **T** |  |  |  |  |  |  | **14** |
| **2** | **290.1347** | 145.5710 | **E** | 1356.7230 | 678.8651 | 1340.7043 | 670.8558 | **1341.7121** | 671.3597 | **13** |
| **3** | 404.1776 | 202.5924 | **N** | 1227.6804 | 614.3438 | **1211.6617** | 606.3345 | **1212.6695** | 606.8384 | **12** |
| **4** | 491.2096 | 246.1084 | **S** | 1113.6375 | 557.3224 | **1097.6188** | 549.3130 | **1098.6266** | 549.8169 | **11** |
| **5** | 592.2573 | 296.6323 | **T** | 1026.6055 | 513.8064 | 1010.5867 | 505.7970 | 1011.5946 | 506.3009 | **10** |
| **6** | 679.2893 | 340.1483 | **S** | 925.5578 | 463.2825 | **909.5391** | 455.2732 | **910.5469** | 455.7771 | **9** |
| **7** | **750.3264** | 375.6669 | **A** | 838.5257 | 419.7665 | **822.5070** | 411.7572 | **823.5148** | 412.2611 | **8** |
| **8** | **847.3792** | 424.1932 | **P** | 767.4886 | 384.2480 | **751.4699** | 376.2386 | **752.4777** | 376.7425 | **7** |
| **9** | 918.4163 | 459.7118 | **A** | 670.4359 | 335.7216 | **654.4171** | 327.7122 | **655.4250** | 328.2161 | **6** |
| **10** | 989.4534 | 495.2304 | **A** | 599.3988 | 300.2030 | **583.3800** | 292.1937 | **584.3879** | 292.6976 | **5** |
| **11** | 1117.5484 | 559.2778 | **K** | 528.3616 | 264.6845 | **512.3429** | 256.6751 | **513.3507** | 257.1790 | **4** |
| **12** | **1214.6012** | 607.8042 | **P** | 400.2667 | 200.6370 | 384.2480 | 192.6276 | 385.2558 | 193.1315 | **3** |
| **13** | **1342.6961** | 671.8517 | **K** | 303.2139 | 152.1106 | **287.1952** | 144.1012 | **288.2030** | 144.6051 | **2** |
| **14** |  |  | **R** | 175.1190 | 88.0631 | 159.1002 | 80.0538 | 160.1081 | 80.5577 | **1** |

**H1FNT,** GI: 44953495

MS/MS Fragmentation of **AKEEAGATAADEGR**, **468.7^3+^**


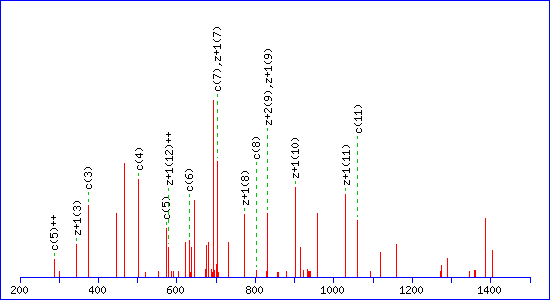


**K2 :** Dimethyl (K)

**Ions Score:** 29 **Expect:** 0.17

**Matches (Red):** 16/104 fragment ions using 32 most intense peaks

| **#** | **c** | **c^++^** | **Seq.** | **y** | **y^++^** | **z+1** | **z+1^++^** | **z+2** | **z+2^++^** | **#** |
| --- | --- | --- | --- | --- | --- | --- | --- | --- | --- | --- |
| **1** | 89.0709 | 45.0391 | **A** |  |  |  |  |  |  | **14** |
| **2** | 245.1972 | 123.1022 | **K** | 1332.6390 | 666.8231 | 1316.6203 | 658.8138 | 1317.6281 | 659.3177 | **13** |
| **3** | **374.2398** | 187.6235 | **E** | 1176.5127 | 588.7600 | 1160.4940 | 580.7506 | 1161.5018 | 581.2546 | **12** |
| **4** | **503.2824** | 252.1448 | **E** | 1047.4701 | 524.2387 | **1031.4514** | 516.2293 | 1032.4592 | 516.7333 | **11** |
| **5** | **574.3195** | 287.6634 | **A** | 918.4276 | 459.7174 | **902.4088** | 451.7081 | 903.4167 | 452.2120 | **10** |
| **6** | **631.3410** | 316.1741 | **G** | 847.3904 | 424.1989 | **831.3717** | 416.1895 | 832.3795 | 416.6934 | **9** |
| **7** | **702.3781** | 351.6927 | **A** | 790.3690 | 395.6881 | **774.3502** | 387.6788 | 775.3581 | 388.1827 | **8** |
| **8** | **803.4258** | 402.2165 | **T** | 719.3319 | 360.1696 | **703.3131** | 352.1602 | 704.3210 | 352.6641 | **7** |
| **9** | 874.4629 | 437.7351 | **A** | 618.2842 | 309.6457 | 602.2655 | 301.6364 | 603.2733 | 302.1403 | **6** |
| **10** | 945.5000 | 473.2536 | **A** | 547.2471 | 274.1272 | 531.2283 | 266.1178 | 532.2362 | 266.6217 | **5** |
| **11** | **1060.5269** | 530.7671 | **D** | 476.2100 | 238.6086 | 460.1912 | 230.5993 | 461.1991 | 231.1032 | **4** |
| **12** | 1189.5695 | 595.2884 | **E** | 361.1830 | 181.0951 | **345.1643** | 173.0858 | 346.1721 | 173.5897 | **3** |
| **13** | 1246.5910 | 623.7991 | **G** | 232.1404 | 116.5738 | 216.1217 | 108.5645 | 217.1295 | 109.0684 | **2** |
| **14** |  |  | **R** | 175.1190 | 88.0631 | 159.1002 | 80.0538 | 160.1081 | 80.5577 | **1** |
