# Supplementary material for: Histone Variants and Their Post-Translational Modifications in Primary Human Fat Cells
Source: PLoS One. 2011 Jan 7;6(1):e15960. doi: 10.1371/journal.pone.0015960 (PMC3017551; doi:10.1371/journal.pone.0015960)
Supplement: Figure S2 — Peptide identification views from MASCOT data analyses of modified peptides from histone H2A (Figure S2A) and histone H2B (Figure S2B) sequenced by electron transfer dissociation of their ions. The spectra, corresponding lists of singly and doubly charged fragment ions and positions of the modified residues identified in the MASCOT search are shown. Additional manual validation of fragment ions with the charge states higher then 2+ had been done for all spectra (not shown) to accomplish and confirm correct peptide sequencing. (DOC) [file pone.0015960.s002.doc]

**Figure S2. Peptide identification views from MASCOT data analyses of modified peptides from histone H2A (Figure S2A) and histone H2B (Figure S2B) sequenced by electron transfer dissociation of their ions.**

**Figure S2A** (pages 1-8)**.**

**Protein similar to H2AFY2,** GI:10433885

MS/MS Fragmentation of **SGRSGKKKMSNLSR, 544.5^3+^**


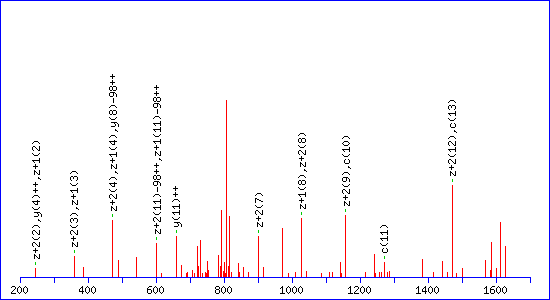


**R3 :** Methyl (R)

**S10 :** Phospho (ST), with neutral losses 0.0000(shown in table), 97.9769

**Ions Score:** 53 **Expect:** 0.001

**Matches (Red):** 19/166 fragment ions using 14 most intense peaks

| **#** | **c** | **c^++^** | **Seq.** | **y** | **y^++^** | **z+1** | **z+1^++^** | **z+2** | **z+2^++^** | **#** |
| --- | --- | --- | --- | --- | --- | --- | --- | --- | --- | --- |
| **1** | 105.0659 | 53.0366 | **S** |  |  |  |  |  |  | **14** |
| **2** | 162.0873 | 81.5473 | **G** | 1542.7934 | 771.9003 | 1526.7747 | 763.8910 | 1527.7825 | 764.3949 | **13** |
| **3** | 332.2041 | 166.6057 | **R** | 1485.7720 | 743.3896 | 1469.7532 | 735.3803 | **1470.7611** | 735.8842 | **12** |
| **4** | 419.2361 | 210.1217 | **S** | 1315.6552 | 658.3312 | 1299.6365 | 650.3219 | 1300.6443 | 650.8258 | **11** |
| **5** | 476.2576 | 238.6324 | **G** | 1228.6232 | 614.8152 | 1212.6044 | 606.8059 | 1213.6123 | 607.3098 | **10** |
| **6** | 604.3525 | 302.6799 | **K** | 1171.6017 | 586.3045 | 1155.5830 | 578.2951 | **1156.5908** | 578.7990 | **9** |
| **7** | 732.4475 | 366.7274 | **K** | 1043.5067 | 522.2570 | **1027.4880** | 514.2476 | **1028.4958** | 514.7516 | **8** |
| **8** | 860.5425 | 430.7749 | **K** | 915.4118 | 458.2095 | 899.3931 | 450.2002 | **900.4009** | 450.7041 | **7** |
| **9** | 991.5829 | 496.2951 | **M** | 787.3168 | 394.1620 | 771.2981 | 386.1527 | 772.3059 | 386.6566 | **6** |
| **10** | **1158.5813** | 579.7943 | **S** | 656.2763 | 328.6418 | 640.2576 | 320.6324 | 641.2654 | 321.1364 | **5** |
| **11** | **1272.6242** | 636.8158 | **N** | 489.2780 | 245.1426 | **473.2592** | 237.1333 | **474.2671** | 237.6372 | **4** |
| **12** | 1385.7083 | 693.3578 | **L** | 375.2350 | 188.1212 | **359.2163** | 180.1118 | **360.2241** | 180.6157 | **3** |
| **13** | **1472.7403** | 736.8738 | **S** | 262.1510 | 131.5791 | **246.1323** | 123.5698 | **247.1401** | 124.0737 | **2** |
| **14** |  |  | **R** | 175.1190 | 88.0631 | 159.1002 | 80.0538 | 160.1081 | 80.5577 | **1** |

**H2AFZ,** GI:4504255

MS/MS Fragmentation of **MAGGKAGKDSGKAKTKAVSRSQR**, **630.3^4+^**


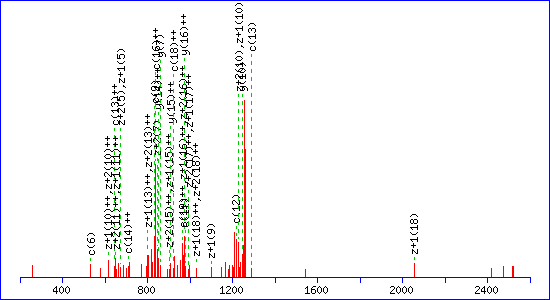


**K12 :** GlyGly (K)

**K16 :** Dimethyl (K)

**R20 :** Dimethyl (R)

**R23 :** Dimethyl (R)

**Ions Score:** 29 **Expect:** 0.97

**Matches (Red):** 36/176 fragment ions using 56 most intense peaks

| **#** | **c** | **c^++^** | **Seq.** | **y** | **y^++^** | **z+1** | **z+1^++^** | **z+2** | **z+2^++^** | **#** |
| --- | --- | --- | --- | --- | --- | --- | --- | --- | --- | --- |
| **1** | 149.0743 | 75.0408 | **M** |  |  |  |  |  |  | **23** |
| **2** | 220.1114 | 110.5593 | **A** | 2386.3637 | 1193.6855 | 2370.3450 | 1185.6761 | 2371.3528 | 1186.1800 | **22** |
| **3** | 277.1329 | 139.0701 | **G** | 2315.3266 | 1158.1669 | 2299.3079 | 1150.1576 | 2300.3157 | 1150.6615 | **21** |
| **4** | 334.1544 | 167.5808 | **G** | 2258.3051 | 1129.6562 | 2242.2864 | 1121.6468 | 2243.2942 | 1122.1508 | **20** |
| **5** | 462.2493 | 231.6283 | **K** | 2201.2837 | 1101.1455 | 2185.2649 | 1093.1361 | 2186.2728 | 1093.6400 | **19** |
| **6** | **533.2864** | 267.1469 | **A** | 2073.1887 | 1037.0980 | **2057.1700** | **1029.0886** | 2058.1778 | **1029.5925** | **18** |
| **7** | 590.3079 | 295.6576 | **G** | 2002.1516 | 1001.5794 | 1986.1329 | **993.5701** | 1987.1407 | **994.0740** | **17** |
| **8** | 718.4029 | 359.7051 | **K** | 1945.1301 | 973.0687 | 1929.1114 | **965.0593** | 1930.1192 | **965.5633** | **16** |
| **9** | **833.4298** | 417.2185 | **D** | 1817.0352 | 909.0212 | 1801.0164 | **901.0119** | 1802.0243 | **901.5158** | **15** |
| **10** | 920.4618 | 460.7346 | **S** | 1702.0082 | 851.5078 | 1685.9895 | 843.4984 | 1686.9973 | 844.0023 | **14** |
| **11** | **977.4833** | 489.2453 | **G** | 1614.9762 | 807.9917 | 1598.9575 | **799.9824** | 1599.9653 | **800.4863** | **13** |
| **12** | **1219.6212** | 610.3142 | **K** | 1557.9547 | 779.4810 | 1541.9360 | 771.4716 | 1542.9438 | 771.9756 | **12** |
| **13** | **1290.6583** | **645.8328** | **A** | 1315.8168 | 658.4121 | 1299.7981 | **650.4027** | 1300.8059 | **650.9066** | **11** |
| **14** | 1418.7533 | **709.8803** | **K** | 1244.7797 | 622.8935 | **1228.7610** | **614.8841** | 1229.7688 | **615.3881** | **10** |
| **15** | 1519.8009 | 760.4041 | **T** | 1116.6848 | 558.8460 | **1100.6660** | 550.8367 | 1101.6739 | 551.3406 | **9** |
| **16** | 1675.9272 | **838.4672** | **K** | 1015.6371 | 508.3222 | 999.6184 | 500.3128 | 1000.6262 | 500.8167 | **8** |
| **17** | 1746.9643 | 873.9858 | **A** | 859.5108 | 430.2591 | 843.4921 | 422.2497 | 844.4999 | 422.7536 | **7** |
| **18** | 1846.0327 | **923.5200** | **V** | 788.4737 | 394.7405 | 772.4550 | 386.7311 | 773.4628 | 387.2350 | **6** |
| **19** | 1933.0648 | **967.0360** | **S** | 689.4053 | 345.2063 | **673.3866** | 337.1969 | 674.3944 | 337.7008 | **5** |
| **20** | 2117.1972 | 1059.1022 | **R** | 602.3733 | 301.6903 | 586.3545 | 293.6809 | 587.3624 | 294.1848 | **4** |
| **21** | 2204.2292 | 1102.6182 | **S** | 418.2409 | 209.6241 | 402.2221 | 201.6147 | 403.2300 | 202.1186 | **3** |
| **22** | 2332.2878 | 1166.6475 | **Q** | 331.2088 | 166.1081 | 315.1901 | 158.0987 | 316.1979 | 158.6026 | **2** |
| **23** |  |  | **R** | 203.1503 | 102.0788 | 187.1315 | 94.0694 | 188.1394 | 94.5733 | **1** |

**histone H2A (fragment),** GI:2118981

MS/MS Fragmentation of **GKQGGKAKSRSSR, 463.6^3+^**


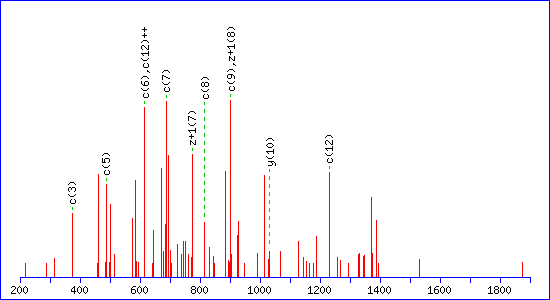


**K2 :** Acetyl (K)

**Ions Score:** 27 **Expect:** 0.38

**Matches (Red):** 11/96 fragment ions using 23 most intense peaks

| **#** | **c** | **c^++^** | **Seq.** | **y** | **y^++^** | **z+1** | **z+1^++^** | **z+2** | **z+2^++^** | **#** |
| --- | --- | --- | --- | --- | --- | --- | --- | --- | --- | --- |
| **1** | 75.0553 | 38.0313 | **G** |  |  |  |  |  |  | **13** |
| **2** | 245.1608 | 123.0840 | **K** | 1331.7502 | 666.3787 | 1315.7315 | 658.3694 | 1316.7393 | 658.8733 | **12** |
| **3** | **373.2194** | 187.1133 | **Q** | 1161.6447 | 581.3260 | 1145.6260 | 573.3166 | 1146.6338 | 573.8205 | **11** |
| **4** | 430.2409 | 215.6241 | **G** | 1033.5861 | 517.2967 | 1017.5674 | 509.2873 | 1018.5752 | 509.7912 | **10** |
| **5** | **487.2623** | 244.1348 | **G** | 976.5647 | 488.7860 | 960.5459 | 480.7766 | 961.5538 | 481.2805 | **9** |
| **6** | **615.3573** | 308.1823 | **K** | 919.5432 | 460.2752 | 903.5245 | 452.2659 | 904.5323 | 452.7698 | **8** |
| **7** | **686.3944** | 343.7008 | **A** | 791.4482 | 396.2278 | 775.4295 | 388.2184 | 776.4373 | 388.7223 | **7** |
| **8** | **814.4894** | 407.7483 | **K** | 720.4111 | 360.7092 | 704.3924 | 352.6998 | 705.4002 | 353.2037 | **6** |
| **9** | **901.5214** | 451.2643 | **S** | 592.3161 | 296.6617 | 576.2974 | 288.6523 | 577.3052 | 289.1563 | **5** |
| **10** | 1057.6225 | 529.3149 | **R** | 505.2841 | 253.1457 | 489.2654 | 245.1363 | 490.2732 | 245.6402 | **4** |
| **11** | 1144.6545 | 572.8309 | **S** | 349.1830 | 175.0951 | 333.1643 | 167.0858 | 334.1721 | 167.5897 | **3** |
| **12** | **1231.6866** | 616.3469 | **S** | 262.1510 | 131.5791 | 246.1323 | 123.5698 | 247.1401 | 124.0737 | **2** |
| **13** |  |  | **R** | 175.1190 | 88.0631 | 159.1002 | 80.0538 | 160.1081 | 80.5577 | **1** |

**H2AFY,** GI: 119582624

MS/MS Fragmentation of **MSSRGGKKKSTKTSR, 570.9^3+^**


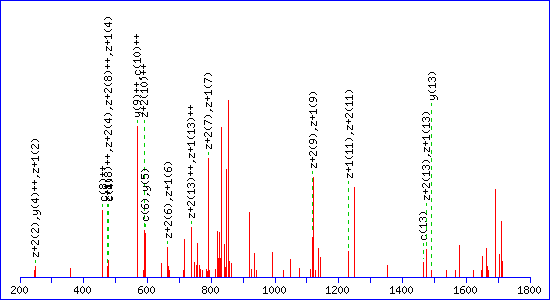


**K7 :** Acetyl (K)

**K8 :** Dimethyl (K)

**Ions Score:** 28 **Expect:** 0.63

**Matches (Red):** 28/112 fragment ions using 35 most intense peaks

| **#** | **c** | **c^++^** | **Seq.** | **y** | **y^++^** | **z+1** | **z+1^++^** | **z+2** | **z+2^++^** | **#** |
| --- | --- | --- | --- | --- | --- | --- | --- | --- | --- | --- |
| **1** | 149.0743 | 75.0408 | **M** |  |  |  |  |  |  | **15** |
| **2** | 236.1063 | 118.5568 | **S** | 1577.9082 | 789.4577 | 1561.8895 | 781.4484 | 1562.8973 | 781.9523 | **14** |
| **3** | 323.1384 | 162.0728 | **S** | 1490.8762 | 745.9417 | **1474.8574** | 737.9324 | **1475.8653** | **738.4363** | **13** |
| **4** | **479.2395** | 240.1234 | **R** | 1403.8441 | 702.4257 | 1387.8254 | 694.4163 | 1388.8332 | 694.9202 | **12** |
| **5** | 536.2609 | 268.6341 | **G** | 1247.7430 | 624.3751 | **1231.7243** | 616.3658 | **1232.7321** | 616.8697 | **11** |
| **6** | **593.2824** | 297.1448 | **G** | 1190.7215 | 595.8644 | 1174.7028 | 587.8550 | 1175.7106 | **588.3590** | **10** |
| **7** | 763.3879 | 382.1976 | **K** | 1133.7001 | 567.3537 | **1117.6814** | 559.3443 | **1118.6892** | 559.8482 | **9** |
| **8** | 919.5142 | 460.2607 | **K** | 963.5946 | 482.3009 | 947.5758 | 474.2916 | 948.5837 | **474.7955** | **8** |
| **9** | 1047.6092 | 524.3082 | **K** | 807.4683 | 404.2378 | **791.4496** | 396.2284 | **792.4574** | 396.7323 | **7** |
| **10** | 1134.6412 | 567.8242 | **S** | 679.3733 | 340.1903 | **663.3546** | 332.1809 | **664.3624** | 332.6849 | **6** |
| **11** | 1235.6889 | 618.3481 | **T** | 592.3413 | 296.6743 | 576.3226 | 288.6649 | 577.3304 | 289.1688 | **5** |
| **12** | 1363.7838 | 682.3956 | **K** | 491.2936 | 246.1504 | **475.2749** | 238.1411 | **476.2827** | 238.6450 | **4** |
| **13** | **1464.8315** | 732.9194 | **T** | 363.1987 | 182.1030 | 347.1799 | 174.0936 | 348.1878 | 174.5975 | **3** |
| **14** | 1551.8635 | 776.4354 | **S** | 262.1510 | 131.5791 | **246.1323** | 123.5698 | **247.1401** | 124.0737 | **2** |
| **15** |  |  | **R** | 175.1190 | 88.0631 | 159.1002 | 80.0538 | 160.1081 | 80.5577 | **1** |

MS/MS Fragmentation of **SSRGGKKKSTKTSR, 549.0^3+^**


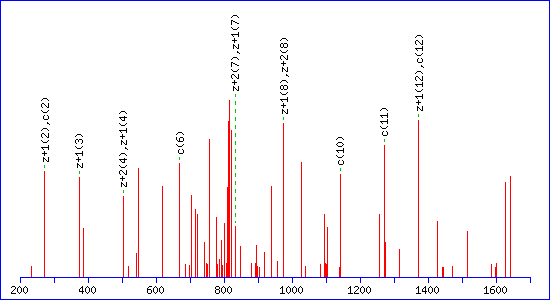


**S2 :** Phospho (ST), with neutral losses 0.0000(shown in table), 97.9769

**K7 :** Methyl (K)

**K8 :** Methyl (K)

**R14 :** Dimethyl (R)

**Ions Score:** 41 **Expect:** 0.023

**Matches (Red):** 14/134 fragment ions using 14 most intense peaks

| **#** | **c** | **c^++^** | **Seq.** | **y** | **y^++^** | **z+1** | **z+1^++^** | **z+2** | **z+2^++^** | **#** |
| --- | --- | --- | --- | --- | --- | --- | --- | --- | --- | --- |
| **1** | 105.0659 | 53.0366 | **S** |  |  |  |  |  |  | **14** |
| **2** | **272.0642** | 136.5357 | **S** | 1556.8632 | 778.9352 | 1540.8445 | 770.9259 | 1541.8523 | 771.4298 | **13** |
| **3** | 428.1653 | 214.5863 | **R** | 1389.8649 | 695.4361 | **1373.8461** | 687.4267 | 1374.8540 | 687.9306 | **12** |
| **4** | 485.1868 | 243.0970 | **G** | 1233.7637 | 617.3855 | 1217.7450 | 609.3761 | 1218.7528 | 609.8801 | **11** |
| **5** | 542.2083 | 271.6078 | **G** | 1176.7423 | 588.8748 | 1160.7236 | 580.8654 | 1161.7314 | 581.3693 | **10** |
| **6** | **670.3032** | 335.6552 | **K** | 1119.7208 | 560.3640 | 1103.7021 | 552.3547 | 1104.7099 | 552.8586 | **9** |
| **7** | 812.4138 | 406.7106 | **K** | 991.6259 | 496.3166 | **975.6071** | 488.3072 | **976.6150** | 488.8111 | **8** |
| **8** | 954.5244 | 477.7659 | **K** | 849.5152 | 425.2613 | **833.4965** | 417.2519 | **834.5043** | 417.7558 | **7** |
| **9** | 1041.5565 | 521.2819 | **S** | 707.4046 | 354.2060 | 691.3859 | 346.1966 | 692.3937 | 346.7005 | **6** |
| **10** | **1142.6041** | 571.8057 | **T** | 620.3726 | 310.6899 | 604.3539 | 302.6806 | 605.3617 | 303.1845 | **5** |
| **11** | **1270.6991** | 635.8532 | **K** | 519.3249 | 260.1661 | **503.3062** | 252.1567 | **504.3140** | 252.6606 | **4** |
| **12** | **1371.7468** | 686.3770 | **T** | 391.2300 | 196.1186 | **375.2112** | 188.1093 | 376.2191 | 188.6132 | **3** |
| **13** | 1458.7788 | 729.8930 | **S** | 290.1823 | 145.5948 | **274.1636** | 137.5854 | 275.1714 | 138.0893 | **2** |
| **14** |  |  | **R** | 203.1503 | 102.0788 | 187.1315 | 94.0694 | 188.1394 | 94.5733 | **1** |

MS/MS Fragmentation of **SSRGGKKKSTKTSR**, **549.2^3+^**


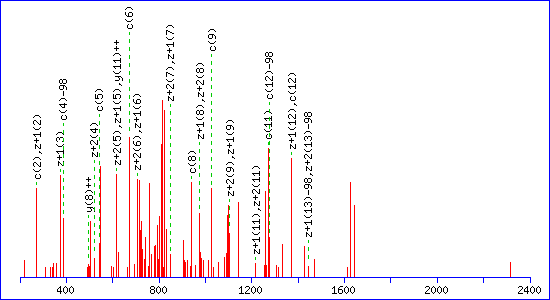


**S2 :** Phospho (ST), with neutral losses 0.0000(shown in table), 97.9769

**K8 :** Methyl (K)

**K11 :** Methyl (K)

**R14 :** Dimethyl (R)

**Ions Score:** 36 **Expect:** 0.065

**Matches (Red):** 29/134 fragment ions using 48 most intense peaks

| **#** | **c** | **c^++^** | **Seq.** | **y** | **y^++^** | **z+1** | **z+1^++^** | **z+2** | **z+2^++^** | **#** |
| --- | --- | --- | --- | --- | --- | --- | --- | --- | --- | --- |
| **1** | 105.0659 | 53.0366 | **S** |  |  |  |  |  |  | **14** |
| **2** | **272.0642** | 136.5357 | **S** | 1556.8632 | 778.9352 | 1540.8445 | 770.9259 | 1541.8523 | 771.4298 | **13** |
| **3** | 428.1653 | 214.5863 | **R** | 1389.8649 | 695.4361 | **1373.8461** | 687.4267 | 1374.8540 | 687.9306 | **12** |
| **4** | 485.1868 | 243.0970 | **G** | 1233.7637 | 617.3855 | **1217.7450** | 609.3761 | **1218.7528** | 609.8801 | **11** |
| **5** | **542.2083** | 271.6078 | **G** | 1176.7423 | 588.8748 | 1160.7236 | 580.8654 | 1161.7314 | 581.3693 | **10** |
| **6** | **670.3032** | 335.6552 | **K** | 1119.7208 | 560.3640 | **1103.7021** | 552.3547 | **1104.7099** | 552.8586 | **9** |
| **7** | 798.3982 | 399.7027 | **K** | 991.6259 | 496.3166 | **975.6071** | 488.3072 | **976.6150** | 488.8111 | **8** |
| **8** | **940.5088** | 470.7580 | **K** | 863.5309 | 432.2691 | **847.5122** | 424.2597 | **848.5200** | 424.7636 | **7** |
| **9** | **1027.5408** | 514.2740 | **S** | 721.4203 | 361.2138 | **705.4016** | 353.2044 | **706.4094** | 353.7083 | **6** |
| **10** | 1128.5885 | 564.7979 | **T** | 634.3883 | 317.6978 | **618.3695** | 309.6884 | **619.3774** | 310.1923 | **5** |
| **11** | **1270.6991** | 635.8532 | **K** | 533.3406 | 267.1739 | 517.3218 | 259.1646 | **518.3297** | 259.6685 | **4** |
| **12** | **1371.7468** | 686.3770 | **T** | 391.2300 | 196.1186 | **375.2112** | 188.1093 | 376.2191 | 188.6132 | **3** |
| **13** | 1458.7788 | 729.8930 | **S** | 290.1823 | 145.5948 | **274.1636** | 137.5854 | 275.1714 | 138.0893 | **2** |
| **14** |  |  | **R** | 203.1503 | 102.0788 | 187.1315 | 94.0694 | 188.1394 | 94.5733 | **1** |

MS/MS Fragmentation of **SSRGGKKKSTKTSR, 549.2^3+^**


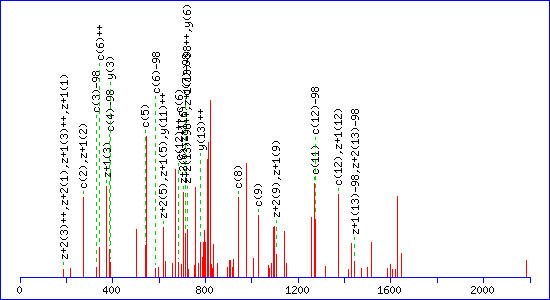


**S2 :** Phospho (ST), with neutral losses 97.9769(shown in table), 0.0000

**K6 :** Methyl (K)

**K11 :** Methyl (K)

**R14 :** Dimethyl (R)

**Ions Score:** 31 **Expect:** 0.24

**Matches (Red):** 35/134 fragment ions using 59 most intense peaks

| **#** | **c** | **c^++^** | **Seq.** | **y** | **y^++^** | **z+1** | **z+1^++^** | **z+2** | **z+2^++^** | **#** |
| --- | --- | --- | --- | --- | --- | --- | --- | --- | --- | --- |
| **1** | 105.0659 | 53.0366 | **S** |  |  |  |  |  |  | **14** |
| **2** | 174.0873 | 87.5473 | **S** | 1458.8863 | 729.9468 | **1442.8676** | 721.9374 | **1443.8754** | 722.4413 | **13** |
| **3** | **330.1884** | 165.5979 | **R** | 1389.8649 | 695.4361 | **1373.8461** | 687.4267 | 1374.8540 | 687.9306 | **12** |
| **4** | **387.2099** | 194.1086 | **G** | 1233.7637 | 617.3855 | 1217.7450 | 609.3761 | 1218.7528 | 609.8801 | **11** |
| **5** | 444.2314 | 222.6193 | **G** | 1176.7423 | 588.8748 | 1160.7236 | 580.8654 | 1161.7314 | 581.3693 | **10** |
| **6** | **586.3420** | 293.6746 | **K** | 1119.7208 | 560.3640 | **1103.7021** | 552.3547 | **1104.7099** | 552.8586 | **9** |
| **7** | **714.4369** | 357.7221 | **K** | 977.6102 | 489.3087 | 961.5915 | 481.2994 | 962.5993 | 481.8033 | **8** |
| **8** | 842.5319 | 421.7696 | **K** | 849.5152 | 425.2613 | 833.4965 | 417.2519 | 834.5043 | 417.7558 | **7** |
| **9** | 929.5639 | 465.2856 | **S** | 721.4203 | 361.2138 | **705.4016** | 353.2044 | **706.4094** | 353.7083 | **6** |
| **10** | 1030.6116 | 515.8094 | **T** | 634.3883 | 317.6978 | **618.3695** | 309.6884 | **619.3774** | 310.1923 | **5** |
| **11** | 1172.7222 | 586.8647 | **K** | 533.3406 | 267.1739 | 517.3218 | 259.1646 | 518.3297 | 259.6685 | **4** |
| **12** | **1273.7699** | 637.3886 | **T** | 391.2300 | 196.1186 | **375.2112** | 188.1093 | 376.2191 | 188.6132 | **3** |
| **13** | 1360.8019 | 680.9046 | **S** | 290.1823 | 145.5948 | **274.1636** | 137.5854 | 275.1714 | 138.0893 | **2** |
| **14** |  |  | **R** | 203.1503 | 102.0788 | **187.1315** | 94.0694 | **188.1394** | 94.5733 | **1** |

MS/MS Fragmentation of **GKKKSTKTSR, 533.6^3+^**


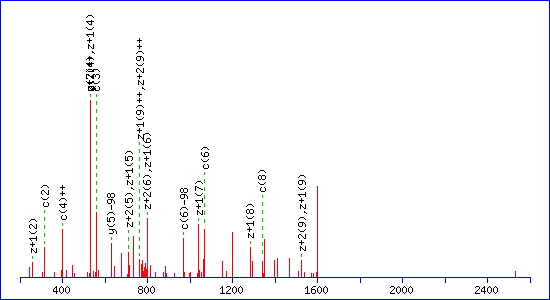


**K2 :** GlyGly (K)

**K3 :** GlyGly (K)

**K4 :** GlyGly (K)

**T6 :** Phospho (ST), with neutral losses 0.0000(shown in table), 97.9769

**K7 :** Acetyl (K)

**R10 :** Methyl (R)

**Ions Score:** 32 **Expect:** 0.14

**Matches (Red):** 21/110 fragment ions using 28 most intense peaks

| **#** | **c** | **c^++^** | **Seq.** | **y** | **y^++^** | **z+1** | **z+1^++^** | **z+2** | **z+2^++^** | **#** |
| --- | --- | --- | --- | --- | --- | --- | --- | --- | --- | --- |
| **1** | 75.0553 | 38.0313 | **G** |  |  |  |  |  |  | **10** |
| **2** | **317.1932** | 159.1002 | **K** | 1541.7795 | 771.3934 | **1525.7608** | 763.3840 | **1526.7686** | 763.8880 | **9** |
| **3** | **559.3311** | 280.1692 | **K** | 1299.6417 | 650.3245 | **1283.6229** | 642.3151 | 1284.6308 | 642.8190 | **8** |
| **4** | 801.4690 | 401.2381 | **K** | 1057.5038 | 529.2555 | **1041.4850** | 521.2462 | 1042.4929 | 521.7501 | **7** |
| **5** | 888.5010 | 444.7541 | **S** | 815.3659 | 408.1866 | **799.3472** | 400.1772 | **800.3550** | 400.6811 | **6** |
| **6** | **1069.5150** | 535.2611 | **T** | 728.3338 | 364.6706 | **712.3151** | 356.6612 | **713.3229** | 357.1651 | **5** |
| **7** | 1239.6205 | 620.3139 | **K** | 547.3198 | 274.1636 | **531.3011** | 266.1542 | **532.3089** | 266.6581 | **4** |
| **8** | **1340.6682** | 670.8377 | **T** | 377.2143 | 189.1108 | 361.1956 | 181.1014 | 362.2034 | 181.6053 | **3** |
| **9** | 1427.7002 | 714.3538 | **S** | 276.1666 | 138.5870 | **260.1479** | 130.5776 | 261.1557 | 131.0815 | **2** |
| **10** |  |  | **R** | 189.1346 | 95.0709 | 173.1159 | 87.0616 | 174.1237 | 87.5655 | **1** |

**Figure S2B. Peptide identification views from MASCOT data analyses of modified peptides from histone H2B sequenced by electron transfer dissociation of their ions.**

**HIST1H2BM protein,** GI:45768638

MS/MS Fragmentation of **PEPVKSAPVPKKGSKKAINKAQKKDGKKRKR, 642.4^6+^**


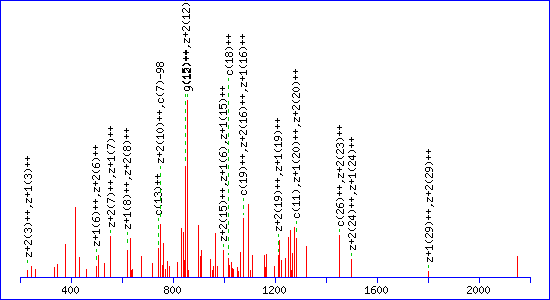


**K5 :** Acetyl (K)

**S6 :** Phospho (ST), with neutral losses 0.0000(shown in table), 97.9769

**K11 :** Methyl (K)

**K12 :** Methyl (K)

**K16 :** Dimethyl (K)

**K27 :** GlyGly (K)

**K28 :** GlyGly (K)

**K30 :** Methyl (K)

**Ions Score:** 44 **Expect:** 0.096

**Matches (Red):** 32/320 fragment ions using 45 most intense peaks

| **#** | **c** | **c^++^** | **Seq.** | **y** | **y^++^** | **z+1** | **z+1^++^** | **z+2** | **z+2^++^** | **#** |
| --- | --- | --- | --- | --- | --- | --- | --- | --- | --- | --- |
| **1** | 115.0866 | 58.0469 | **P** |  |  |  |  |  |  | **31** |
| **2** | 244.1292 | 122.5682 | **E** | 3750.1742 | 1875.5907 | 3734.1555 | 1867.5814 | 3735.1633 | 1868.0853 | **30** |
| **3** | 341.1819 | 171.0946 | **P** | 3621.1316 | 1811.0694 | 3605.1129 | **1803.0601** | 3606.1207 | **1803.5640** | **29** |
| **4** | 440.2504 | 220.6288 | **V** | 3524.0788 | 1762.5431 | 3508.0601 | 1754.5337 | 3509.0680 | 1755.0376 | **28** |
| **5** | 610.3559 | 305.6816 | **K** | 3425.0104 | 1713.0089 | 3408.9917 | 1704.9995 | 3409.9995 | 1705.5034 | **27** |
| **6** | 777.3542 | 389.1808 | **S** | 3254.9049 | 1627.9561 | 3238.8862 | 1619.9467 | 3239.8940 | 1620.4506 | **26** |
| **7** | 848.3914 | 424.6993 | **A** | 3087.9065 | 1544.4569 | 3071.8878 | 1536.4476 | 3072.8956 | 1536.9515 | **25** |
| **8** | 945.4441 | 473.2257 | **P** | 3016.8694 | 1508.9384 | 3000.8507 | **1500.9290** | 3001.8585 | **1501.4329** | **24** |
| **9** | 1044.5125 | 522.7599 | **V** | 2919.8167 | 1460.4120 | 2903.7979 | 1452.4026 | 2904.8058 | **1452.9065** | **23** |
| **10** | 1141.5653 | 571.2863 | **P** | 2820.7483 | 1410.8778 | 2804.7295 | 1402.8684 | 2805.7374 | 1403.3723 | **22** |
| **11** | 1283.6759 | 642.3416 | **K** | 2723.6955 | 1362.3514 | 2707.6768 | 1354.3420 | 2708.6846 | 1354.8459 | **21** |
| **12** | 1425.7865 | 713.3969 | **K** | 2581.5849 | 1291.2961 | 2565.5662 | **1283.2867** | 2566.5740 | **1283.7906** | **20** |
| **13** | 1482.8080 | **741.9076** | **G** | 2439.4743 | 1220.2408 | 2423.4555 | **1212.2314** | 2424.4634 | **1212.7353** | **19** |
| **14** | 1569.8400 | 785.4236 | **S** | 2382.4528 | 1191.7300 | 2366.4341 | 1183.7207 | 2367.4419 | 1184.2246 | **18** |
| **15** | 1697.9350 | **849.4711** | **K** | 2295.4208 | 1148.2140 | 2279.4021 | 1140.2047 | 2280.4099 | 1140.7086 | **17** |
| **16** | 1854.0612 | 927.5343 | **K** | 2167.3258 | 1084.1665 | 2151.3071 | **1076.1572** | 2152.3149 | **1076.6611** | **16** |
| **17** | 1925.0984 | 963.0528 | **A** | 2011.1995 | 1006.1034 | 1995.1808 | **998.0941** | 1996.1886 | **998.5980** | **15** |
| **18** | 2038.1824 | **1019.5949** | **I** | 1940.1624 | 970.5849 | 1924.1437 | 962.5755 | 1925.1515 | 963.0794 | **14** |
| **19** | 2152.2254 | **1076.6163** | **N** | 1827.0784 | 914.0428 | 1811.0596 | 906.0335 | 1812.0675 | 906.5374 | **13** |
| **20** | 2280.3203 | 1140.6638 | **K** | 1713.0354 | 857.0214 | 1697.0167 | 849.0120 | 1698.0245 | **849.5159** | **12** |
| **21** | 2351.3574 | 1176.1824 | **A** | 1584.9405 | 792.9739 | 1568.9218 | 784.9645 | 1569.9296 | 785.4684 | **11** |
| **22** | 2479.4160 | 1240.2116 | **Q** | 1513.9034 | 757.4553 | 1497.8846 | 749.4460 | 1498.8925 | **749.9499** | **10** |
| **23** | 2607.5110 | 1304.2591 | **K** | 1385.8448 | 693.4260 | 1369.8261 | 685.4167 | 1370.8339 | 685.9206 | **9** |
| **24** | 2735.6059 | 1368.3066 | **K** | 1257.7498 | 629.3786 | 1241.7311 | **621.3692** | 1242.7389 | **621.8731** | **8** |
| **25** | 2850.6329 | 1425.8201 | **D** | 1129.6549 | 565.3311 | 1113.6361 | **557.3217** | 1114.6440 | **557.8256** | **7** |
| **26** | 2907.6543 | **1454.3308** | **G** | 1014.6279 | 507.8176 | 998.6092 | **499.8082** | 999.6170 | **500.3121** | **6** |
| **27** | 3149.7922 | 1575.3998 | **K** | 957.6065 | 479.3069 | 941.5877 | 471.2975 | 942.5956 | 471.8014 | **5** |
| **28** | 3391.9301 | 1696.4687 | **K** | 715.4686 | 358.2379 | 699.4498 | 350.2286 | 700.4577 | 350.7325 | **4** |
| **29** | 3548.0312 | 1774.5193 | **R** | 473.3307 | 237.1690 | 457.3120 | **229.1596** | 458.3198 | **229.6635** | **3** |
| **30** | 3690.1418 | 1845.5746 | **K** | 317.2296 | 159.1184 | 301.2108 | 151.1091 | 302.2187 | 151.6130 | **2** |
| **31** |  |  | **R** | 175.1190 | 88.0631 | 159.1002 | 80.0538 | 160.1081 | 80.5577 | **1** |

MS/MS Fragmentation of **LLLPGELAKHAVSEGTKAVTKYTSSKCASR, 543.9^6+^**


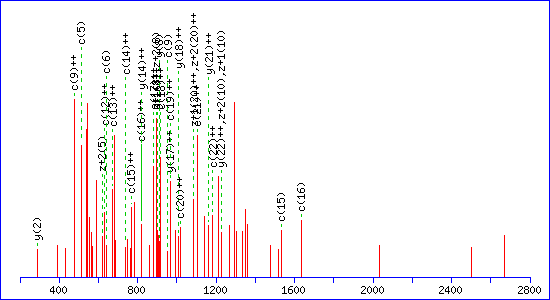


**K21 :** Acetyl (K)

**K26 :** Acetyl (K)

**R30 :** Dimethyl (R)

**Ions Score:** 37 **Expect:** 0.2

**Matches (Red):** 32/232 fragment ions using 66 most intense peaks

| **#** | **c** | **c^++^** | **Seq.** | **y** | **y^++^** | **z+1** | **z+1^++^** | **z+2** | **z+2^++^** | **#** |
| --- | --- | --- | --- | --- | --- | --- | --- | --- | --- | --- |
| **1** | 131.1179 | 66.0626 | **L** |  |  |  |  |  |  | **30** |
| **2** | 244.2020 | 122.6046 | **L** | 3144.6722 | 1572.8397 | 3128.6534 | 1564.8304 | 3129.6613 | 1565.3343 | **29** |
| **3** | 357.2860 | 179.1466 | **L** | 3031.5881 | 1516.2977 | 3015.5694 | 1508.2883 | 3016.5772 | 1508.7922 | **28** |
| **4** | 454.3388 | 227.6730 | **P** | 2918.5040 | 1459.7557 | 2902.4853 | 1451.7463 | 2903.4931 | 1452.2502 | **27** |
| **5** | **511.3602** | 256.1838 | **G** | 2821.4513 | 1411.2293 | 2805.4325 | 1403.2199 | 2806.4404 | 1403.7238 | **26** |
| **6** | **640.4028** | 320.7051 | **E** | 2764.4298 | 1382.7185 | 2748.4111 | 1374.7092 | 2749.4189 | 1375.2131 | **25** |
| **7** | 753.4869 | 377.2471 | **L** | 2635.3872 | 1318.1972 | 2619.3685 | 1310.1879 | 2620.3763 | 1310.6918 | **24** |
| **8** | 824.5240 | 412.7656 | **A** | 2522.3031 | 1261.6552 | 2506.2844 | 1253.6459 | 2507.2923 | 1254.1498 | **23** |
| **9** | **952.6190** | **476.8131** | **K** | 2451.2660 | 1226.1367 | 2435.2473 | 1218.1273 | 2436.2551 | 1218.6312 | **22** |
| **10** | 1089.6779 | 545.3426 | **H** | 2323.1711 | 1162.0892 | 2307.1523 | 1154.0798 | 2308.1602 | 1154.5837 | **21** |
| **11** | 1160.7150 | 580.8611 | **A** | 2186.1122 | 1093.5597 | 2170.0934 | 1085.5504 | 2171.1013 | 1086.0543 | **20** |
| **12** | 1259.7834 | **630.3953** | **V** | 2115.0750 | 1058.0412 | 2099.0563 | 1050.0318 | 2100.0641 | 1050.5357 | **19** |
| **13** | 1346.8154 | **673.9114** | **S** | 2016.0066 | 1008.5070 | 1999.9879 | 1000.4976 | 2000.9957 | 1001.0015 | **18** |
| **14** | 1475.8580 | **738.4327** | **E** | 1928.9746 | 964.9909 | 1912.9559 | 956.9816 | 1913.9637 | 957.4855 | **17** |
| **15** | **1532.8795** | **766.9434** | **G** | 1799.9320 | 900.4696 | 1783.9133 | 892.4603 | 1784.9211 | 892.9642 | **16** |
| **16** | **1633.9272** | **817.4672** | **T** | 1742.9105 | 871.9589 | 1726.8918 | 863.9496 | 1727.8996 | 864.4535 | **15** |
| **17** | 1762.0221 | **881.5147** | **K** | 1641.8629 | 821.4351 | 1625.8441 | 813.4257 | 1626.8520 | 813.9296 | **14** |
| **18** | 1833.0593 | **917.0333** | **A** | 1513.7679 | 757.3876 | 1497.7492 | 749.3782 | 1498.7570 | 749.8821 | **13** |
| **19** | 1932.1277 | **966.5675** | **V** | 1442.7308 | 721.8690 | 1426.7121 | 713.8597 | 1427.7199 | 714.3636 | **12** |
| **20** | 2033.1754 | **1017.0913** | **T** | 1343.6624 | 672.3348 | 1327.6437 | 664.3255 | 1328.6515 | 664.8294 | **11** |
| **21** | 2203.2809 | **1102.1441** | **K** | 1242.6147 | 621.8110 | 1226.5960 | 613.8016 | 1227.6038 | 614.3055 | **10** |
| **22** | 2366.3442 | **1183.6757** | **Y** | 1072.5092 | 536.7582 | 1056.4904 | 528.7489 | 1057.4983 | 529.2528 | **9** |
| **23** | 2467.3919 | 1234.1996 | **T** | 909.4458 | 455.2266 | 893.4271 | 447.2172 | 894.4349 | 447.7211 | **8** |
| **24** | 2554.4239 | 1277.7156 | **S** | 808.3982 | 404.7027 | 792.3794 | 396.6934 | 793.3873 | 397.1973 | **7** |
| **25** | 2641.4559 | 1321.2316 | **S** | 721.3661 | 361.1867 | 705.3474 | 353.1773 | 706.3552 | 353.6813 | **6** |
| **26** | 2811.5615 | 1406.2844 | **K** | 634.3341 | 317.6707 | 618.3154 | 309.6613 | 619.3232 | 310.1652 | **5** |
| **27** | 2914.5707 | 1457.7890 | **C** | 464.2286 | 232.6179 | 448.2099 | 224.6086 | 449.2177 | 225.1125 | **4** |
| **28** | 2985.6078 | 1493.3075 | **A** | 361.2194 | 181.1133 | 345.2007 | 173.1040 | 346.2085 | 173.6079 | **3** |
| **29** | 3072.6398 | 1536.8235 | **S** | 290.1823 | 145.5948 | 274.1636 | 137.5854 | 275.1714 | 138.0893 | **2** |
| **30** |  |  | **R** | 203.1503 | 102.0788 | 187.1315 | 94.0694 | 188.1394 | 94.5733 | **1** |

**HIST1H2BA,** GI:42542570

MS/MS Fragmentation of **IASEAPRLAHYSKR, 588.8^3+^**


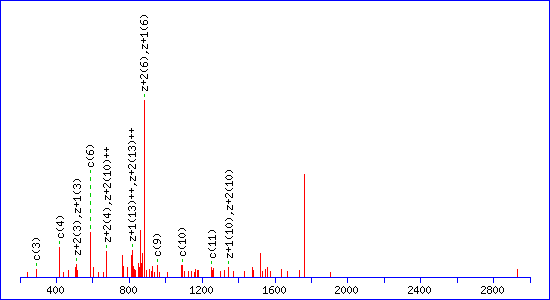


**R7 :** Dimethyl (R)

**S12 :** Phospho (ST), with neutral losses 0.0000(shown in table), 97.9769

**K13 :** Acetyl (K)

**R14 :** Methyl (R)

**Ions Score:** 38 **Expect:** 0.12

**Matches (Red):** 16/174 fragment ions using 15 most intense peaks

| **#** | **c** | **c^++^** | **Seq.** | **y** | **y^++^** | **z+1** | **z+1^++^** | **z+2** | **z+2^++^** | **#** |
| --- | --- | --- | --- | --- | --- | --- | --- | --- | --- | --- |
| **1** | 131.1179 | 66.0626 | **I** |  |  |  |  |  |  | **14** |
| **2** | 202.1550 | 101.5811 | **A** | 1649.8159 | 825.4116 | 1633.7972 | 817.4022 | 1634.8050 | **817.9062** | **13** |
| **3** | **289.1870** | 145.0972 | **S** | 1578.7788 | 789.8930 | 1562.7601 | 781.8837 | 1563.7679 | 782.3876 | **12** |
| **4** | **418.2296** | 209.6184 | **E** | 1491.7468 | 746.3770 | 1475.7281 | 738.3677 | 1476.7359 | 738.8716 | **11** |
| **5** | 489.2667 | 245.1370 | **A** | 1362.7042 | 681.8557 | **1346.6855** | 673.8464 | **1347.6933** | **674.3503** | **10** |
| **6** | **586.3195** | 293.6634 | **P** | 1291.6671 | 646.3372 | 1275.6484 | 638.3278 | 1276.6562 | 638.8317 | **9** |
| **7** | 770.4519 | 385.7296 | **R** | 1194.6143 | 597.8108 | 1178.5956 | 589.8014 | 1179.6034 | 590.3053 | **8** |
| **8** | 883.5360 | 442.2716 | **L** | 1010.4819 | 505.7446 | 994.4632 | 497.7352 | 995.4710 | 498.2391 | **7** |
| **9** | **954.5731** | 477.7902 | **A** | 897.3978 | 449.2026 | **881.3791** | 441.1932 | **882.3869** | 441.6971 | **6** |
| **10** | **1091.6320** | 546.3196 | **H** | 826.3607 | 413.6840 | 810.3420 | 405.6746 | 811.3498 | 406.1786 | **5** |
| **11** | **1254.6953** | 627.8513 | **Y** | 689.3018 | 345.1545 | 673.2831 | 337.1452 | **674.2909** | 337.6491 | **4** |
| **12** | 1421.6937 | 711.3505 | **S** | 526.2385 | 263.6229 | **510.2198** | 255.6135 | **511.2276** | 256.1174 | **3** |
| **13** | 1591.7992 | 796.4032 | **K** | 359.2401 | 180.1237 | 343.2214 | 172.1143 | 344.2292 | 172.6183 | **2** |
| **14** |  |  | **R** | 189.1346 | 95.0709 | 173.1159 | 87.0616 | 174.1237 | 87.5655 | **1** |

MS/MS Fragmentation of **IASEAPRLAHYSKR, 442.0^4+^**


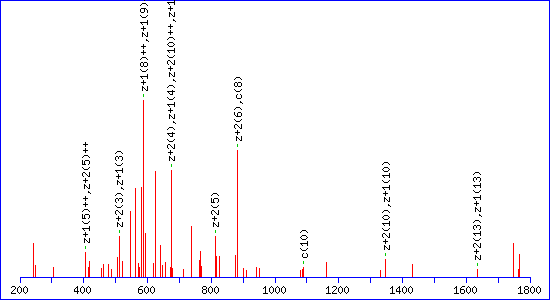


**R7 :** Dimethyl (R)

**S12 :** Phospho (ST), with neutral losses 0.0000(shown in table), 97.9769

**K13 :** Acetyl (K)

**R14 :** Methyl (R)

**Ions Score:** 37 **Expect:** 0.17

**Matches (Red):** 19/174 fragment ions using 14 most intense peaks

| **#** | **c** | **c^++^** | **Seq.** | **y** | **y^++^** | **z+1** | **z+1^++^** | **z+2** | **z+2^++^** | **#** |
| --- | --- | --- | --- | --- | --- | --- | --- | --- | --- | --- |
| **1** | 131.1179 | 66.0626 | **I** |  |  |  |  |  |  | **14** |
| **2** | 202.1550 | 101.5811 | **A** | 1649.8159 | 825.4116 | **1633.7972** | 817.4022 | **1634.8050** | 817.9062 | **13** |
| **3** | 289.1870 | 145.0972 | **S** | 1578.7788 | 789.8930 | 1562.7601 | 781.8837 | 1563.7679 | 782.3876 | **12** |
| **4** | 418.2296 | 209.6184 | **E** | 1491.7468 | 746.3770 | 1475.7281 | 738.3677 | 1476.7359 | 738.8716 | **11** |
| **5** | 489.2667 | 245.1370 | **A** | 1362.7042 | 681.8557 | **1346.6855** | **673.8464** | **1347.6933** | **674.3503** | **10** |
| **6** | 586.3195 | 293.6634 | **P** | 1291.6671 | 646.3372 | 1275.6484 | 638.3278 | 1276.6562 | 638.8317 | **9** |
| **7** | 770.4519 | 385.7296 | **R** | 1194.6143 | 597.8108 | 1178.5956 | **589.8014** | 1179.6034 | 590.3053 | **8** |
| **8** | **883.5360** | 442.2716 | **L** | 1010.4819 | 505.7446 | 994.4632 | 497.7352 | 995.4710 | 498.2391 | **7** |
| **9** | 954.5731 | 477.7902 | **A** | 897.3978 | 449.2026 | 881.3791 | 441.1932 | **882.3869** | 441.6971 | **6** |
| **10** | **1091.6320** | 546.3196 | **H** | 826.3607 | 413.6840 | 810.3420 | **405.6746** | **811.3498** | **406.1786** | **5** |
| **11** | 1254.6953 | 627.8513 | **Y** | 689.3018 | 345.1545 | **673.2831** | 337.1452 | **674.2909** | 337.6491 | **4** |
| **12** | 1421.6937 | 711.3505 | **S** | 526.2385 | 263.6229 | **510.2198** | 255.6135 | **511.2276** | 256.1174 | **3** |
| **13** | 1591.7992 | 796.4032 | **K** | 359.2401 | 180.1237 | 343.2214 | 172.1143 | 344.2292 | 172.6183 | **2** |
| **14** |  |  | **R** | 189.1346 | 95.0709 | 173.1159 | 87.0616 | 174.1237 | 87.5655 | **1** |

MS/MS Fragmentation of **STISSREIQTAVR**, **488.4^3+^**


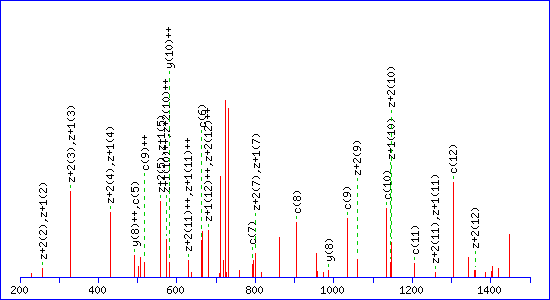


**R6 :** Methyl (R)

**Ions Score:** 47 **Expect:** 0.0064

**Matches (Red):** 34/96 fragment ions using 50 most intense peaks

| **#** | **c** | **c^++^** | **Seq.** | **y** | **y^++^** | **z+1** | **z+1^++^** | **z+2** | **z+2^++^** | **#** |
| --- | --- | --- | --- | --- | --- | --- | --- | --- | --- | --- |
| **1** | 105.0659 | 53.0366 | **S** |  |  |  |  |  |  | **13** |
| **2** | 206.1135 | 103.5604 | **T** | 1374.7700 | 687.8886 | 1358.7512 | 679.8793 | **1359.7591** | 680.3832 | **12** |
| **3** | 319.1976 | 160.1024 | **I** | 1273.7223 | 637.3648 | **1257.7036** | 629.3554 | **1258.7114** | 629.8593 | **11** |
| **4** | 406.2296 | 203.6184 | **S** | 1160.6382 | 580.8227 | **1144.6195** | 572.8134 | **1145.6273** | 573.3173 | **10** |
| **5** | **493.2617** | 247.1345 | **S** | 1073.6062 | 537.3067 | 1057.5875 | 529.2974 | **1058.5953** | 529.8013 | **9** |
| **6** | **663.3784** | 332.1928 | **R** | 986.5742 | 493.7907 | 970.5554 | 485.7814 | 971.5633 | 486.2853 | **8** |
| **7** | **792.4210** | 396.7141 | **E** | 816.4574 | 408.7323 | **800.4387** | 400.7230 | **801.4465** | 401.2269 | **7** |
| **8** | **905.5051** | 453.2562 | **I** | 687.4148 | 344.2110 | 671.3961 | 336.2017 | 672.4039 | 336.7056 | **6** |
| **9** | **1033.5636** | 517.2855 | **Q** | 574.3307 | 287.6690 | **558.3120** | 279.6596 | **559.3198** | 280.1636 | **5** |
| **10** | **1134.6113** | 567.8093 | **T** | 446.2722 | 223.6397 | **430.2534** | 215.6304 | **431.2613** | 216.1343 | **4** |
| **11** | **1205.6484** | 603.3279 | **A** | 345.2245 | 173.1159 | **329.2058** | 165.1065 | **330.2136** | 165.6104 | **3** |
| **12** | **1304.7169** | 652.8621 | **V** | 274.1874 | 137.5973 | **258.1686** | 129.5880 | **259.1765** | 130.0919 | **2** |
| **13** |  |  | **R** | 175.1190 | 88.0631 | 159.1002 | 80.0538 | 160.1081 | 80.5577 | **1** |

**HIST1H2BN,** GI:119623520

MS/MS Fragmentation of **LLLPGELAKHAVSEGTKAVTKYTSSKKR, 543.8^6+^**


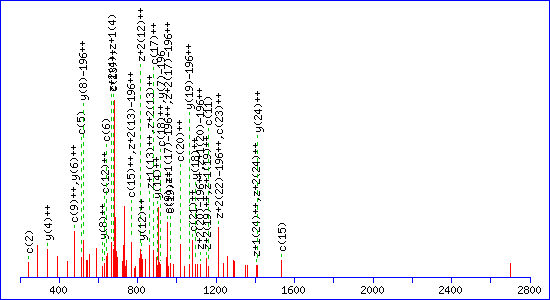


**S24 :** Phospho (ST), with neutral losses 0.0000(shown in table), 97.9769

**S25 :** Phospho (ST), with neutral losses 0.0000(shown in table), 97.9769

**K26 :** Acetyl (K)

**K27 :** Acetyl (K)

**Ions Score:** 40 **Expect:** 0.09

**Matches (Red):** 41/368 fragment ions using 65 most intense peaks

| **#** | **c** | **c^++^** | **Seq.** | **y** | **y^++^** | **z+1** | **z+1^++^** | **z+2** | **z+2^++^** | **#** |
| --- | --- | --- | --- | --- | --- | --- | --- | --- | --- | --- |
| **1** | 131.1179 | 66.0626 | **L** |  |  |  |  |  |  | **28** |
| **2** | **244.2020** | 122.6046 | **L** | 3143.5902 | 1572.2987 | 3127.5714 | 1564.2894 | 3128.5793 | 1564.7933 | **27** |
| **3** | 357.2860 | 179.1466 | **L** | 3030.5061 | 1515.7567 | 3014.4874 | 1507.7473 | 3015.4952 | 1508.2512 | **26** |
| **4** | 454.3388 | 227.6730 | **P** | 2917.4220 | 1459.2147 | 2901.4033 | 1451.2053 | 2902.4111 | 1451.7092 | **25** |
| **5** | **511.3602** | 256.1838 | **G** | 2820.3693 | 1410.6883 | 2804.3505 | 1402.6789 | 2805.3584 | **1403.1828** | **24** |
| **6** | **640.4028** | 320.7051 | **E** | 2763.3478 | 1382.1775 | 2747.3291 | 1374.1682 | 2748.3369 | 1374.6721 | **23** |
| **7** | 753.4869 | 377.2471 | **L** | 2634.3052 | 1317.6562 | 2618.2865 | 1309.6469 | 2619.2943 | 1310.1508 | **22** |
| **8** | 824.5240 | 412.7656 | **A** | 2521.2211 | 1261.1142 | 2505.2024 | 1253.1048 | 2506.2102 | 1253.6088 | **21** |
| **9** | **952.6190** | **476.8131** | **K** | 2450.1840 | 1225.5957 | 2434.1653 | 1217.5863 | 2435.1731 | 1218.0902 | **20** |
| **10** | 1089.6779 | 545.3426 | **H** | 2322.0891 | 1161.5482 | 2306.0703 | 1153.5388 | 2307.0782 | **1154.0427** | **19** |
| **11** | **1160.7150** | 580.8611 | **A** | 2185.0302 | 1093.0187 | 2169.0114 | 1085.0094 | 2170.0193 | 1085.5133 | **18** |
| **12** | 1259.7834 | **630.3953** | **V** | 2113.9930 | 1057.5002 | 2097.9743 | 1049.4908 | 2098.9821 | 1049.9947 | **17** |
| **13** | 1346.8154 | **673.9114** | **S** | 2014.9246 | 1007.9660 | 1998.9059 | 999.9566 | 1999.9137 | 1000.4605 | **16** |
| **14** | 1475.8580 | 738.4327 | **E** | 1927.8926 | 964.4499 | 1911.8739 | 956.4406 | 1912.8817 | 956.9445 | **15** |
| **15** | **1532.8795** | **766.9434** | **G** | 1798.8500 | 899.9286 | 1782.8313 | 891.9193 | 1783.8391 | 892.4232 | **14** |
| **16** | 1633.9272 | 817.4672 | **T** | 1741.8285 | 871.4179 | 1725.8098 | 863.4085 | 1726.8176 | **863.9125** | **13** |
| **17** | 1762.0221 | **881.5147** | **K** | 1640.7809 | 820.8941 | 1624.7621 | 812.8847 | 1625.7700 | **813.3886** | **12** |
| **18** | 1833.0593 | **917.0333** | **A** | 1512.6859 | 756.8466 | 1496.6672 | 748.8372 | 1497.6750 | 749.3411 | **11** |
| **19** | 1932.1277 | **966.5675** | **V** | 1441.6488 | 721.3280 | 1425.6301 | 713.3187 | 1426.6379 | 713.8226 | **10** |
| **20** | 2033.1754 | **1017.0913** | **T** | 1342.5804 | 671.7938 | 1326.5617 | 663.7845 | 1327.5695 | 664.2884 | **9** |
| **21** | 2161.2703 | **1081.1388** | **K** | 1241.5327 | 621.2700 | 1225.5140 | 613.2606 | 1226.5218 | 613.7645 | **8** |
| **22** | 2324.3336 | 1162.6705 | **Y** | 1113.4377 | 557.2225 | 1097.4190 | 549.2131 | 1098.4268 | 549.7171 | **7** |
| **23** | 2425.3813 | **1213.1943** | **T** | 950.3744 | 475.6908 | 934.3557 | 467.6815 | 935.3635 | 468.1854 | **6** |
| **24** | 2592.3797 | 1296.6935 | **S** | 849.3267 | 425.1670 | 833.3080 | 417.1576 | 834.3158 | 417.6616 | **5** |
| **25** | 2759.3780 | 1380.1927 | **S** | 682.3284 | 341.6678 | 666.3096 | 333.6585 | 667.3175 | 334.1624 | **4** |
| **26** | 2929.4836 | 1465.2454 | **K** | 515.3300 | 258.1686 | 499.3113 | 250.1593 | 500.3191 | 250.6632 | **3** |
| **27** | 3099.5891 | 1550.2982 | **K** | 345.2245 | 173.1159 | 329.2058 | 165.1065 | 330.2136 | 165.6104 | **2** |
| **28** |  |  | **R** | 175.1190 | 88.0631 | 159.1002 | 80.0538 | 160.1081 | 80.5577 | **1** |

MS/MS Fragmentation of **LLLPGELAKHAVSEGTKAVTKYTSSKKR, 547.4^6+^**


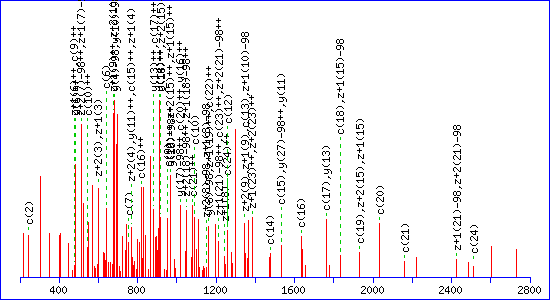


**S25 :** Phospho (ST), with neutral losses 97.9769(shown in table), 0.0000

**K26 :** Acetyl (K)

**K27 :** GlyGly (K)

**R28 :** Dimethyl (R)

**Ions Score:** 77 **Expect:** 1.7e-05

**Matches (Red):** 76/366 fragment ions using 85 most intense peaks

| **#** | **c** | **c^++^** | **Seq.** | **y** | **y^++^** | **z+1** | **z+1^++^** | **z+2** | **z+2^++^** | **#** |
| --- | --- | --- | --- | --- | --- | --- | --- | --- | --- | --- |
| **1** | 131.1179 | 66.0626 | **L** |  |  |  |  |  |  | **28** |
| **2** | **244.2020** | 122.6046 | **L** | 3065.7106 | 1533.3589 | 3049.6919 | 1525.3496 | 3050.6997 | 1525.8535 | **27** |
| **3** | 357.2860 | 179.1466 | **L** | 2952.6265 | 1476.8169 | 2936.6078 | 1468.8075 | 2937.6156 | 1469.3115 | **26** |
| **4** | 454.3388 | 227.6730 | **P** | 2839.5425 | 1420.2749 | 2823.5237 | 1412.2655 | 2824.5316 | 1412.7694 | **25** |
| **5** | **511.3602** | 256.1838 | **G** | 2742.4897 | 1371.7485 | 2726.4710 | 1363.7391 | 2727.4788 | 1364.2430 | **24** |
| **6** | **640.4028** | 320.7051 | **E** | 2685.4682 | 1343.2378 | 2669.4495 | 1335.2284 | 2670.4573 | 1335.7323 | **23** |
| **7** | **753.4869** | 377.2471 | **L** | 2556.4256 | 1278.7165 | 2540.4069 | 1270.7071 | 2541.4147 | 1271.2110 | **22** |
| **8** | 824.5240 | 412.7656 | **A** | 2443.3416 | 1222.1744 | **2427.3229** | **1214.1651** | 2428.3307 | **1214.6690** | **21** |
| **9** | **952.6190** | **476.8131** | **K** | 2372.3045 | 1186.6559 | 2356.2857 | 1178.6465 | 2357.2936 | 1179.1504 | **20** |
| **10** | **1089.6779** | **545.3426** | **H** | 2244.2095 | 1122.6084 | 2228.1908 | 1114.5990 | 2229.1986 | 1115.1029 | **19** |
| **11** | 1160.7150 | 580.8611 | **A** | 2107.1506 | 1054.0789 | 2091.1319 | **1046.0696** | 2092.1397 | **1046.5735** | **18** |
| **12** | **1259.7834** | 630.3953 | **V** | 2036.1135 | 1018.5604 | 2020.0948 | 1010.5510 | 2021.1026 | 1011.0549 | **17** |
| **13** | **1346.8154** | **673.9114** | **S** | 1937.0451 | 969.0262 | 1921.0263 | 961.0168 | 1922.0342 | 961.5207 | **16** |
| **14** | **1475.8580** | 738.4327 | **E** | 1850.0130 | 925.5102 | **1833.9943** | **917.5008** | 1835.0021 | **918.0047** | **15** |
| **15** | **1532.8795** | **766.9434** | **G** | 1720.9704 | 860.9889 | 1704.9517 | 852.9795 | 1705.9595 | 853.4834 | **14** |
| **16** | **1633.9272** | **817.4672** | **T** | 1663.9490 | 832.4781 | 1647.9303 | 824.4688 | 1648.9381 | 824.9727 | **13** |
| **17** | **1762.0221** | **881.5147** | **K** | 1562.9013 | 781.9543 | 1546.8826 | 773.9449 | 1547.8904 | 774.4488 | **12** |
| **18** | **1833.0593** | **917.0333** | **A** | 1434.8063 | 717.9068 | 1418.7876 | 709.8974 | 1419.7954 | 710.4014 | **11** |
| **19** | **1932.1277** | **966.5675** | **V** | 1363.7692 | 682.3883 | **1347.7505** | **674.3789** | 1348.7583 | **674.8828** | **10** |
| **20** | **2033.1754** | **1017.0913** | **T** | 1264.7008 | 632.8540 | 1248.6821 | 624.8447 | 1249.6899 | 625.3486 | **9** |
| **21** | **2161.2703** | **1081.1388** | **K** | 1163.6531 | 582.3302 | **1147.6344** | 574.3208 | 1148.6422 | 574.8248 | **8** |
| **22** | 2324.3336 | **1162.6705** | **Y** | 1035.5582 | 518.2827 | 1019.5394 | **510.2734** | 1020.5473 | **510.7773** | **7** |
| **23** | 2425.3813 | **1213.1943** | **T** | 872.4948 | 436.7511 | 856.4761 | 428.7417 | 857.4839 | 429.2456 | **6** |
| **24** | **2512.4134** | **1256.7103** | **S** | 771.4472 | 386.2272 | 755.4284 | 378.2179 | 756.4363 | 378.7218 | **5** |
| **25** | 2581.4348 | 1291.2210 | **S** | 684.4151 | 342.7112 | 668.3964 | 334.7018 | 669.4042 | 335.2058 | **4** |
| **26** | 2751.5403 | 1376.2738 | **K** | 615.3937 | 308.2005 | **599.3749** | 300.1911 | 600.3828 | 300.6950 | **3** |
| **27** | 2993.6782 | 1497.3428 | **K** | 445.2881 | 223.1477 | 429.2694 | 215.1383 | 430.2772 | 215.6423 | **2** |
| **28** |  |  | **R** | 203.1503 | 102.0788 | 187.1315 | 94.0694 | 188.1394 | 94.5733 | **1** |
