# Supplementary material for: Histone Variants and Their Post-Translational Modifications in Primary Human Fat Cells
Source: PLoS One. 2011 Jan 7;6(1):e15960. doi: 10.1371/journal.pone.0015960 (PMC3017551; doi:10.1371/journal.pone.0015960)
Supplement: Figure S3 — Peptide identification views from MASCOT data analyses of modified peptides from histone H3 sequenced by electron transfer dissociation of their ions. The spectra, corresponding lists of singly and doubly charged fragment ions and positions of the modified residues identified in the MASCOT search are shown. (DOC) [file pone.0015960.s003.doc]

**Figure S3. Peptide identification views from MASCOT data analyses of modified peptides from histone H3 sequenced by electron transfer dissociation of their ions.**

**H3F3A/H3F3B,** GI: 45044279

MS/MS Fragmentation of **KSAPSTGGVKKPHR**, **503.0^3+^**


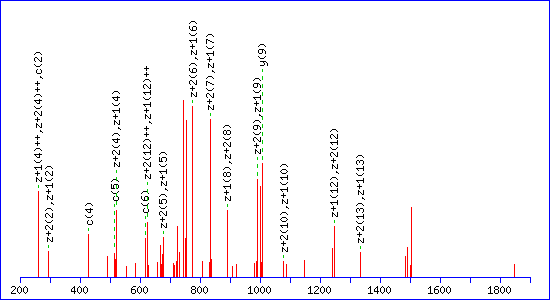


**K1 :** Dimethyl (K)

**K10 :** Dimethyl (K)

**Ions Score:** 60 **Expect:** 0.00031

**Matches (Red):** 29/104 fragment ions using 39 most intense peaks

| **#** | **c** | **c^++^** | **Seq.** | **y** | **y^++^** | **z+1** | **z+1^++^** | **z+2** | **z+2^++^** | **#** |
| --- | --- | --- | --- | --- | --- | --- | --- | --- | --- | --- |
| **1** | 174.1601 | 87.5837 | **K** |  |  |  |  |  |  | **14** |
| **2** | **261.1921** | 131.0997 | **S** | 1349.7648 | 675.3860 | ***1333.7461*** | 667.3767 | **1334.7539** | 667.8806 | **13** |
| **3** | 332.2292 | 166.6183 | **A** | 1262.7328 | 631.8700 | ***1246.7141*** | 623.8607 | **1247.7219** | 624.3646 | **12** |
| **4** | **429.2820** | 215.1446 | **P** | 1191.6957 | 596.3515 | 1175.6769 | 588.3421 | 1176.6848 | 588.8460 | **11** |
| **5** | **516.3140** | 258.6606 | **S** | 1094.6429 | 547.8251 | ***1078.6242*** | 539.8157 | **1079.6320** | 540.3196 | **10** |
| **6** | **617.3617** | 309.1845 | **T** | 1007.6109 | 504.3091 | ***991.5922*** | 496.2997 | **992.6000** | 496.8036 | **9** |
| **7** | 674.3832 | 337.6952 | **G** | 906.5632 | 453.7852 | ***890.5445*** | 445.7759 | **891.5523** | 446.2798 | **8** |
| **8** | 731.4046 | 366.2060 | **G** | 849.5417 | 425.2745 | ***833.5230*** | 417.2651 | **834.5308** | 417.7691 | **7** |
| **9** | 830.4730 | 415.7402 | **V** | 792.5203 | 396.7638 | ***776.5015*** | 388.7544 | **777.5094** | 389.2583 | **6** |
| **10** | 986.5993 | 493.8033 | **K** | 693.4519 | 347.2296 | ***677.4331*** | 339.2202 | **678.4410** | 339.7241 | **5** |
| **11** | 1114.6943 | 557.8508 | **K** | 537.3256 | 269.1664 | ***521.3069*** | 261.1571 | **522.3147** | 261.6610 | **4** |
| **12** | 1211.7470 | 606.3772 | **P** | 409.2306 | 205.1190 | 393.2119 | 197.1096 | 394.2197 | 197.6135 | **3** |
| **13** | 1348.8059 | 674.9066 | **H** | 312.1779 | 156.5926 | ***296.1591*** | 148.5832 | **297.1670** | 149.0871 | **2** |
| **14** |  |  | **R** | 175.1190 | 88.0631 | 159.1002 | 80.0538 | 160.1081 | 80.5577 | **1** |

**H3F3A/H3F3B,** GI: 45044279 and **HIST1H3I,** GI: 45219796

MS/MS Fragmentation of **KQLATKAAR, 515.1^2+^**


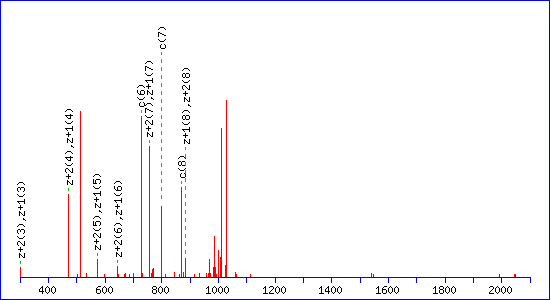


**K6 :** Acetyl (K)

**Ions Score:** 41 **Expect:** 0.019

**Matches (Red):** 15/64 fragment ions using 21 most intense peaks

| **#** | **c** | **c^++^** | **Seq.** | **y** | **y^++^** | **z+1** | **z+1^++^** | **z+2** | **z+2^++^** | **#** |
| --- | --- | --- | --- | --- | --- | --- | --- | --- | --- | --- |
| **1** | 146.1288 | 73.5680 | **K** |  |  |  |  |  |  | **9** |
| **2** | 274.1874 | 137.5973 | **Q** | 900.5261 | 450.7667 | **884.5074** | 442.7573 | **885.5152** | 443.2613 | **8** |
| **3** | 387.2714 | 194.1394 | **L** | 772.4676 | 386.7374 | **756.4488** | 378.7281 | **757.4567** | 379.2320 | **7** |
| **4** | 458.3085 | 229.6579 | **A** | 659.3835 | 330.1954 | **643.3648** | 322.1860 | **644.3726** | 322.6899 | **6** |
| **5** | 559.3562 | 280.1817 | **T** | 588.3464 | 294.6768 | **572.3277** | 286.6675 | **573.3355** | 287.1714 | **5** |
| **6** | **729.4618** | 365.2345 | **K** | 487.2987 | 244.1530 | **471.2800** | 236.1436 | **472.2878** | 236.6475 | **4** |
| **7** | **800.4989** | 400.7531 | **A** | 317.1932 | 159.1002 | **301.1745** | 151.0909 | **302.1823** | 151.5948 | **3** |
| **8** | **871.5360** | 436.2716 | **A** | 246.1561 | 123.5817 | 230.1373 | 115.5723 | 231.1452 | 116.0762 | **2** |
| **9** |  |  | **R** | 175.1190 | 88.0631 | 159.1002 | 80.0538 | 160.1081 | 80.5577 | **1** |
