# Supplementary material for: Histone Variants and Their Post-Translational Modifications in Primary Human Fat Cells
Source: PLoS One. 2011 Jan 7;6(1):e15960. doi: 10.1371/journal.pone.0015960 (PMC3017551; doi:10.1371/journal.pone.0015960)
Supplement: Figure S4 — Peptide identification views from MASCOT data analyses of modified peptides from histone H4 sequenced by electron transfer dissociation of their ions. The spectra, corresponding lists of singly and doubly charged fragment ions and positions of the modified residues identified in the MASCOT search are shown. Additional manual validation of fragment ions with the charge states higher then 2+ had been done for all spectra (not shown) to accomplish and confirm correct peptide sequencing. (DOC) [file pone.0015960.s004.doc]

**Figure S4. Peptide identification views from MASCOT data analyses of modified peptides from histone H4 sequenced by electron transfer dissociation of their ions.**

**HIST2H4B,** GI:124504316

MS/MS Fragmentation of **VWRGKGGKGLGKGGAKR, 647.6^3+^**


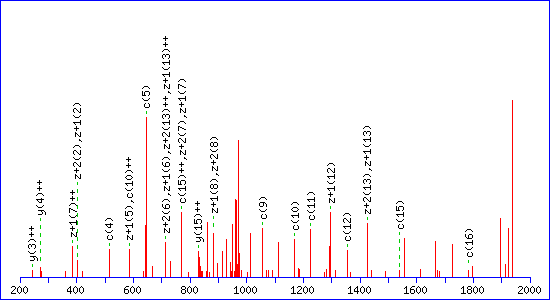


**K8 :** GlyGly (K)

**K16 :** GlyGly (K)

**Ions Score:** 45 **Expect:** 0.016

**Matches (Red):** 28/128 fragment ions using 34 most intense peaks

| **#** | **c** | **c^++^** | **Seq.** | **y** | **y^++^** | **z+1** | **z+1^++^** | **z+2** | **z+2^++^** | **#** |
| --- | --- | --- | --- | --- | --- | --- | --- | --- | --- | --- |
| **1** | 117.1022 | 59.0548 | **V** |  |  |  |  |  |  | **17** |
| **2** | 303.1816 | 152.0944 | **W** | 1841.0365 | 921.0219 | 1825.0178 | 913.0125 | 1826.0256 | 913.5164 | **16** |
| **3** | 459.2827 | 230.1450 | **R** | 1654.9572 | 827.9822 | 1638.9385 | 819.9729 | 1639.9463 | 820.4768 | **15** |
| **4** | **516.3041** | 258.6557 | **G** | 1498.8561 | 749.9317 | 1482.8374 | 741.9223 | 1483.8452 | 742.4262 | **14** |
| **5** | **644.3991** | 322.7032 | **K** | 1441.8346 | 721.4209 | **1425.8159** | 713.4116 | **1426.8237** | 713.9155 | **13** |
| **6** | 701.4206 | 351.2139 | **G** | 1313.7397 | 657.3735 | **1297.7209** | 649.3641 | 1298.7288 | 649.8680 | **12** |
| **7** | 758.4420 | 379.7246 | **G** | 1256.7182 | 628.8627 | 1240.6995 | 620.8534 | 1241.7073 | 621.3573 | **11** |
| **8** | 1000.5799 | 500.7936 | **K** | 1199.6967 | 600.3520 | 1183.6780 | 592.3426 | 1184.6858 | 592.8466 | **10** |
| **9** | **1057.6014** | 529.3043 | **G** | 957.5588 | 479.2831 | 941.5401 | 471.2737 | 942.5479 | 471.7776 | **9** |
| **10** | **1170.6854** | 585.8464 | **L** | 900.5374 | 450.7723 | **884.5187** | 442.7630 | **885.5265** | 443.2669 | **8** |
| **11** | **1227.7069** | 614.3571 | **G** | 787.4533 | 394.2303 | **771.4346** | 386.2209 | **772.4424** | 386.7248 | **7** |
| **12** | **1355.8019** | 678.4046 | **K** | 730.4318 | 365.7196 | **714.4131** | 357.7102 | **715.4209** | 358.2141 | **6** |
| **13** | 1412.8233 | 706.9153 | **G** | 602.3369 | 301.6721 | **586.3182** | 293.6627 | 587.3260 | 294.1666 | **5** |
| **14** | 1469.8448 | 735.4260 | **G** | 545.3154 | 273.1613 | 529.2967 | 265.1520 | 530.3045 | 265.6559 | **4** |
| **15** | **1540.8819** | 770.9446 | **A** | 488.2940 | 244.6506 | 472.2752 | 236.6413 | 473.2831 | 237.1452 | **3** |
| **16** | **1783.0198** | 892.0135 | **K** | 417.2568 | 209.1321 | **401.2381** | 201.1227 | **402.2459** | 201.6266 | **2** |
| **17** |  |  | **R** | 175.1190 | 88.0631 | 159.1002 | 80.0538 | 160.1081 | 80.5577 | **1** |

MS/MS Fragmentation of **VWRGKGGKGLGKGGAKR**, **585.6^3+^**


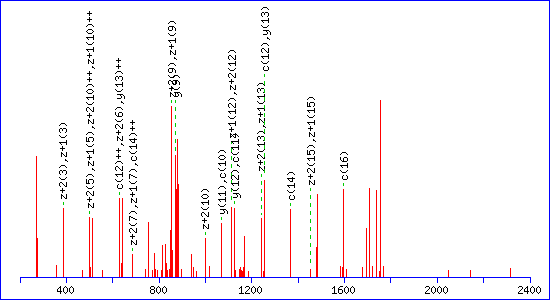


**K8 :** Methyl (K)

**K16 :** Dimethyl (K)

**Ions Score:** 53 **Expect:** 0.001

**Matches (Red):** 31/128 fragment ions using 28 most intense peaks

| **#** | **c** | **c^++^** | **Seq.** | **y** | **y^++^** | **z+1** | **z+1^++^** | **z+2** | **z+2^++^** | **#** |
| --- | --- | --- | --- | --- | --- | --- | --- | --- | --- | --- |
| **1** | 117.1022 | 59.0548 | **V** |  |  |  |  |  |  | **17** |
| **2** | 303.1816 | 152.0944 | **W** | 1654.9976 | 828.0024 | 1638.9789 | 819.9931 | 1639.9867 | 820.4970 | **16** |
| **3** | 459.2827 | 230.1450 | **R** | 1468.9183 | 734.9628 | **1452.8996** | 726.9534 | ***1453.9074*** | 727.4573 | **15** |
| **4** | 516.3041 | 258.6557 | **G** | 1312.8172 | 656.9122 | 1296.7985 | 648.9029 | 1297.8063 | 649.4068 | **14** |
| **5** | 644.3991 | 322.7032 | **K** | **1255.7957** | 628.4015 | **1239.7770** | 620.3921 | ***1240.7848*** | 620.8960 | **13** |
| **6** | 701.4206 | 351.2139 | **G** | **1127.7008** | 564.3540 | **1111.6820** | 556.3447 | ***1112.6899*** | 556.8486 | **12** |
| **7** | 758.4420 | 379.7246 | **G** | **1070.6793** | 535.8433 | 1054.6606 | 527.8339 | 1055.6684 | 528.3378 | **11** |
| **8** | 900.5526 | 450.7800 | **K** | 1013.6578 | 507.3326 | 997.6391 | 499.3232 | ***998.6469*** | 499.8271 | **10** |
| **9** | 957.5741 | 479.2907 | **G** | **871.5472** | 436.2772 | **855.5285** | 428.2679 | ***856.5363*** | 428.7718 | **9** |
| **10** | **1070.6582** | 535.8327 | **L** | 814.5257 | 407.7665 | 798.5070 | 399.7571 | 799.5148 | 400.2611 | **8** |
| **11** | **1127.6796** | 564.3434 | **G** | 701.4417 | 351.2245 | **685.4230** | 343.2151 | ***686.4308*** | 343.7190 | **7** |
| **12** | **1255.7746** | 628.3909 | **K** | 644.4202 | 322.7137 | **628.4015** | 314.7044 | ***629.4093*** | 315.2083 | **6** |
| **13** | 1312.7960 | 656.9017 | **G** | 516.3253 | 258.6663 | **500.3065** | 250.6569 | ***501.3144*** | 251.1608 | **5** |
| **14** | **1369.8175** | 685.4124 | **G** | 459.3038 | 230.1555 | 443.2851 | 222.1462 | 444.2929 | 222.6501 | **4** |
| **15** | 1440.8546 | 720.9310 | **A** | 402.2823 | 201.6448 | **386.2636** | 193.6354 | ***387.2714*** | 194.1394 | **3** |
| **16** | **1596.9809** | 798.9941 | **K** | 331.2452 | 166.1262 | 315.2265 | 158.1169 | 316.2343 | 158.6208 | **2** |
| **17** |  |  | **R** | 175.1190 | 88.0631 | 159.1002 | 80.0538 | 160.1081 | 80.5577 | **1** |

**HIST1H4A-L/HIST2H4A-B/HIST4H4**, GI: 223582

MS/MS Fragmentation of **GKGGKGLGKGGAKR, 485.8^3+^**


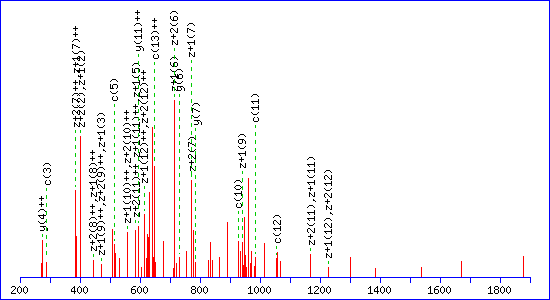


**K2 :** Dimethyl (K)

**K5 :** Acetyl (K)

**K13 :** GlyGly (K)

**Ions Score:** 31 **Expect:** 0.23

**Matches (Red):** 35/104 fragment ions using 51 most intense peaks

| **#** | **c** | **c^++^** | **Seq.** | **y** | **y^++^** | **z+1** | **z+1^++^** | **z+2** | **z+2^++^** | **#** |
| --- | --- | --- | --- | --- | --- | --- | --- | --- | --- | --- |
| **1** | 75.0553 | 38.0313 | **G** |  |  |  |  |  |  | **14** |
| **2** | 231.1816 | 116.0944 | **K** | 1397.8336 | 699.4204 | 1381.8148 | 691.4111 | 1382.8227 | 691.9150 | **13** |
| **3** | **288.2030** | 144.6051 | **G** | 1241.7073 | 621.3573 | **1225.6886** | **613.3479** | **1226.6964** | **613.8518** | **12** |
| **4** | 345.2245 | 173.1159 | **G** | 1184.6858 | 592.8466 | **1168.6671** | **584.8372** | **1169.6749** | **585.3411** | **11** |
| **5** | **515.3300** | 258.1686 | **K** | 1127.6644 | 564.3358 | 1111.6456 | **556.3265** | 1112.6535 | **556.8304** | **10** |
| **6** | 572.3515 | 286.6794 | **G** | 957.5588 | 479.2831 | **941.5401** | **471.2737** | 942.5479 | **471.7776** | **9** |
| **7** | 685.4355 | 343.2214 | **L** | 900.5374 | 450.7723 | 884.5187 | **442.7630** | 885.5265 | **443.2669** | **8** |
| **8** | 742.4570 | 371.7321 | **G** | 787.4533 | 394.2303 | **771.4346** | **386.2209** | **772.4424** | **386.7248** | **7** |
| **9** | 870.5520 | 435.7796 | **K** | 730.4318 | 365.7196 | **714.4131** | 357.7102 | **715.4209** | 358.2141 | **6** |
| **10** | **927.5734** | 464.2904 | **G** | 602.3369 | 301.6721 | **586.3182** | 293.6627 | 587.3260 | 294.1666 | **5** |
| **11** | **984.5949** | 492.8011 | **G** | 545.3154 | 273.1613 | 529.2967 | 265.1520 | 530.3045 | 265.6559 | **4** |
| **12** | **1055.6320** | 528.3196 | **A** | 488.2940 | 244.6506 | **472.2752** | 236.6413 | 473.2831 | 237.1452 | **3** |
| **13** | 1297.7699 | 649.3886 | **K** | 417.2568 | 209.1321 | **401.2381** | 201.1227 | **402.2459** | 201.6266 | **2** |
| **14** |  |  | **R** | 175.1190 | 88.0631 | 159.1002 | 80.0538 | 160.1081 | 80.5577 | **1** |

MS/MS Fragmentation of **GKGGKGLGKGGAKR, 514.6^3+^**


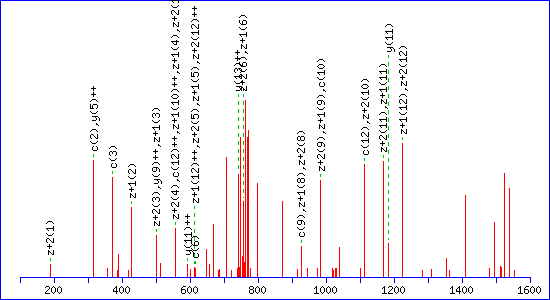


**K2 :** GlyGly (K)

**K9 :** Methyl (K)

**K13 :** GlyGly (K)

**R14 :** Dimethyl (R)

**Ions Score:** 44 **Expect:** 0.018

**Matches (Red):** 35/104 fragment ions using 55 most intense peaks

| **#** | **c** | **c^++^** | **Seq.** | **y** | **y^++^** | **z+1** | **z+1^++^** | **z+2** | **z+2^++^** | **#** |
| --- | --- | --- | --- | --- | --- | --- | --- | --- | --- | --- |
|  |  |  |  |  |  |  |  |  |  |  |
| **1** | 75.0553 | 38.0313 | **G** |  |  |  |  |  |  | **14** |
| **2** | **317.1932** | 159.1002 | **K** | 1483.8816 | 742.4444 | 1467.8628 | 734.4351 | 1468.8707 | 734.9390 | **13** |
| **3** | **374.2146** | 187.6110 | **G** | 1241.7437 | 621.3755 | **1225.7250** | 613.3661 | **1226.7328** | 613.8700 | **12** |
| **4** | 431.2361 | 216.1217 | **G** | 1184.7222 | 592.8647 | **1168.7035** | 584.8554 | **1169.7113** | 585.3593 | **11** |
| **5** | 559.3311 | 280.1692 | **K** | 1127.7008 | 564.3540 | 1111.6820 | 556.3447 | **1112.6899** | 556.8486 | **10** |
| **6** | **616.3525** | 308.6799 | **G** | 999.6058 | 500.3065 | **983.5871** | 492.2972 | **984.5949** | 492.8011 | **9** |
| **7** | 729.4366 | 365.2219 | **L** | 942.5843 | 471.7958 | **926.5656** | 463.7864 | **927.5734** | 464.2904 | **8** |
| **8** | 786.4581 | 393.7327 | **G** | 829.5003 | 415.2538 | 813.4815 | 407.2444 | 814.4894 | 407.7483 | **7** |
| **9** | **928.5687** | 464.7880 | **K** | 772.4788 | 386.7430 | **756.4601** | 378.7337 | **757.4679** | 379.2376 | **6** |
| **10** | **985.5901** | 493.2987 | **G** | 630.3682 | 315.6877 | **614.3495** | 307.6784 | **615.3573** | 308.1823 | **5** |
| **11** | 1042.6116 | 521.8094 | **G** | 573.3467 | 287.1770 | **557.3280** | 279.1676 | **558.3358** | 279.6715 | **4** |
| **12** | **1113.6487** | 557.3280 | **A** | 516.3253 | 258.6663 | **500.3065** | 250.6569 | **501.3144** | 251.1608 | **3** |
| **13** | 1355.7866 | 678.3969 | **K** | 445.2881 | 223.1477 | **429.2694** | 215.1383 | 430.2772 | 215.6423 | **2** |
| **14** |  |  | **R** | 203.1503 | 102.0788 | 187.1315 | 94.0694 | **188.1394** | 94.5733 | **1** |

MS/MS Fragmentation of **KVLRDNIQGITKPAIR**, **618.3^3+^**


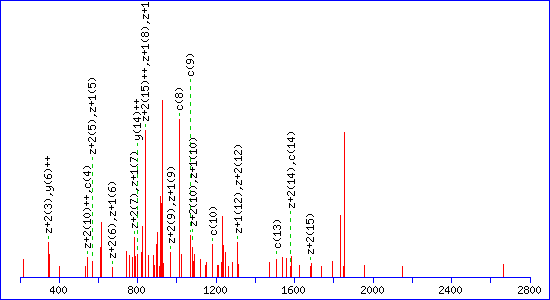


**K1 :** Dimethyl (K)

**Ions Score:** 41 **Expect:** 0.027

**Matches (Red):** 29/120 fragment ions using 45 most intense peaks

| **#** | **c** | **c^++^** | **Seq.** | **y** | **y^++^** | **z+1** | **z+1^++^** | **z+2** | **z+2^++^** | **#** |
| --- | --- | --- | --- | --- | --- | --- | --- | --- | --- | --- |
| **1** | 174.1601 | 87.5837 | **K** |  |  |  |  |  |  | **16** |
| **2** | 273.2285 | 137.1179 | **V** | 1694.0072 | 847.5072 | 1677.9884 | 839.4979 | **1678.9963** | 840.0018 | **15** |
| **3** | 386.3126 | 193.6599 | **L** | 1594.9388 | 797.9730 | 1578.9200 | 789.9637 | **1579.9279** | 790.4676 | **14** |
| **4** | **542.4137** | 271.7105 | **R** | 1481.8547 | 741.4310 | 1465.8360 | 733.4216 | 1466.8438 | 733.9255 | **13** |
| **5** | 657.4406 | 329.2239 | **D** | 1325.7536 | 663.3804 | **1309.7349** | 655.3711 | **1310.7427** | 655.8750 | **12** |
| **6** | 771.4835 | 386.2454 | **N** | 1210.7266 | 605.8670 | 1194.7079 | 597.8576 | 1195.7157 | 598.3615 | **11** |
| **7** | 884.5676 | 442.7874 | **I** | 1096.6837 | 548.8455 | **1080.6650** | 540.8361 | **1081.6728** | 541.3400 | **10** |
| **8** | **1012.6262** | 506.8167 | **Q** | 983.5996 | 492.3035 | **967.5809** | 484.2941 | **968.5887** | 484.7980 | **9** |
| **9** | **1069.6477** | 535.3275 | **G** | 855.5411 | 428.2742 | **839.5223** | 420.2648 | **840.5302** | 420.7687 | **8** |
| **10** | **1182.7317** | 591.8695 | **I** | 798.5196 | 399.7634 | **782.5009** | 391.7541 | **783.5087** | 392.2580 | **7** |
| **11** | 1283.7794 | 642.3933 | **T** | 685.4355 | 343.2214 | **669.4168** | 335.2120 | **670.4246** | 335.7160 | **6** |
| **12** | 1411.8744 | 706.4408 | **K** | 584.3879 | 292.6976 | **568.3691** | 284.6882 | **569.3770** | 285.1921 | **5** |
| **13** | **1508.9271** | 754.9672 | **P** | 456.2929 | 228.6501 | 440.2742 | 220.6407 | 441.2820 | 221.1446 | **4** |
| **14** | **1579.9642** | 790.4858 | **A** | 359.2401 | 180.1237 | **343.2214** | 172.1143 | **344.2292** | 172.6183 | **3** |
| **15** | 1693.0483 | 847.0278 | **I** | 288.2030 | 144.6051 | 272.1843 | 136.5958 | 273.1921 | 137.0997 | **2** |
| **16** |  |  | **R** | 175.1190 | 88.0631 | 159.1002 | 80.0538 | 160.1081 | 80.5577 | **1** |

MS/MS Fragmentation of **KVLRDDIQGITKPAIR, 617.7^3+^**


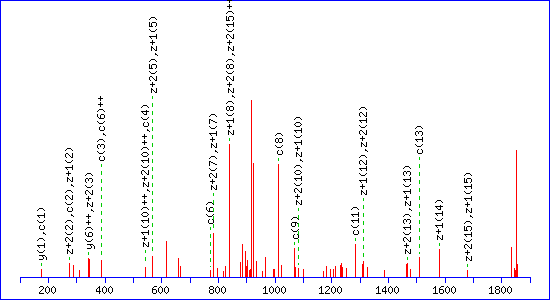


**K1 :** Dimethyl (K)

**Ions Score:** 75 **Expect:** 2.1e-05

**Matches (Red):** 35/120 fragment ions using 30 most intense peaks

| **#** | **c** | **c^++^** | **Seq.** | **y** | **y^++^** | **z+1** | **z+1^++^** | **z+2** | **z+2^++^** | **#** |
| --- | --- | --- | --- | --- | --- | --- | --- | --- | --- | --- |
| **1** | **174.1601** | 87.5837 | **K** |  |  |  |  |  |  | **16** |
| **2** | **273.2285** | 137.1179 | **V** | 1694.9912 | 847.9992 | **1678.9725** | 839.9899 | **1679.9803** | 840.4938 | **15** |
| **3** | **386.3126** | 193.6599 | **L** | 1595.9228 | 798.4650 | **1579.9040** | 790.4557 | 1580.9119 | 790.9596 | **14** |
| **4** | **542.4137** | 271.7105 | **R** | 1482.8387 | 741.9230 | **1466.8200** | 733.9136 | **1467.8278** | 734.4175 | **13** |
| **5** | 657.4406 | 329.2239 | **D** | 1326.7376 | 663.8724 | **1310.7189** | 655.8631 | **1311.7267** | 656.3670 | **12** |
| **6** | **772.4676** | 386.7374 | **D** | 1211.7106 | 606.3590 | 1195.6919 | 598.3496 | 1196.6997 | 598.8535 | **11** |
| **7** | 885.5516 | 443.2795 | **I** | 1096.6837 | 548.8455 | **1080.6650** | 540.8361 | **1081.6728** | 541.3400 | **10** |
| **8** | **1013.6102** | 507.3087 | **Q** | 983.5996 | 492.3035 | 967.5809 | 484.2941 | 968.5887 | 484.7980 | **9** |
| **9** | **1070.6317** | 535.8195 | **G** | 855.5411 | 428.2742 | **839.5223** | 420.2648 | **840.5302** | 420.7687 | **8** |
| **10** | 1183.7157 | 592.3615 | **I** | 798.5196 | 399.7634 | **782.5009** | 391.7541 | **783.5087** | 392.2580 | **7** |
| **11** | **1284.7634** | 642.8853 | **T** | 685.4355 | 343.2214 | 669.4168 | 335.2120 | 670.4246 | 335.7160 | **6** |
| **12** | 1412.8584 | 706.9328 | **K** | 584.3879 | 292.6976 | **568.3691** | 284.6882 | **569.3770** | 285.1921 | **5** |
| **13** | **1509.9111** | 755.4592 | **P** | 456.2929 | 228.6501 | 440.2742 | 220.6407 | 441.2820 | 221.1446 | **4** |
| **14** | 1580.9483 | 790.9778 | **A** | 359.2401 | 180.1237 | **343.2214** | 172.1143 | **344.2292** | 172.6183 | **3** |
| **15** | 1694.0323 | 847.5198 | **I** | 288.2030 | 144.6051 | **272.1843** | 136.5958 | **273.1921** | 137.0997 | **2** |
| **16** |  |  | **R** | 175.1190 | 88.0631 | 159.1002 | 80.0538 | 160.1081 | 80.5577 | **1** |

MS/MS Fragmentation of **KVLRDDIQGITKPAIR, 464.0^4+^**


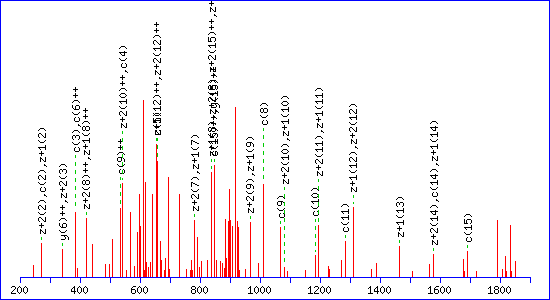


**K1 :** Dimethyl (K)

**Ions Score:** 81 **Expect:** 4.3e-06

**Matches (Red):** 41/120 fragment ions using 33 most intense peaks

| **#** | **c** | **c^++^** | **Seq.** | **y** | **y^++^** | **z+1** | **z+1^++^** | **z+2** | **z+2^++^** | **#** |
| --- | --- | --- | --- | --- | --- | --- | --- | --- | --- | --- |
| **1** | 174.1601 | 87.5837 | **K** |  |  |  |  |  |  | **16** |
| **2** | **273.2285** | 137.1179 | **V** | 1694.9912 | 847.9992 | 1678.9725 | **839.9899** | 1679.9803 | **840.4938** | **15** |
| **3** | **386.3126** | 193.6599 | **L** | 1595.9228 | 798.4650 | **1579.9040** | 790.4557 | **1580.9119** | 790.9596 | **14** |
| **4** | **542.4137** | 271.7105 | **R** | 1482.8387 | 741.9230 | **1466.8200** | 733.9136 | 1467.8278 | 734.4175 | **13** |
| **5** | **657.4406** | 329.2239 | **D** | 1326.7376 | 663.8724 | **1310.7189** | **655.8631** | **1311.7267** | **656.3670** | **12** |
| **6** | 772.4676 | **386.7374** | **D** | 1211.7106 | 606.3590 | **1195.6919** | 598.3496 | **1196.6997** | 598.8535 | **11** |
| **7** | 885.5516 | 443.2795 | **I** | 1096.6837 | 548.8455 | **1080.6650** | 540.8361 | **1081.6728** | **541.3400** | **10** |
| **8** | **1013.6102** | 507.3087 | **Q** | 983.5996 | 492.3035 | **967.5809** | 484.2941 | **968.5887** | 484.7980 | **9** |
| **9** | **1070.6317** | **535.8195** | **G** | 855.5411 | 428.2742 | **839.5223** | **420.2648** | **840.5302** | **420.7687** | **8** |
| **10** | **1183.7157** | 592.3615 | **I** | 798.5196 | 399.7634 | **782.5009** | 391.7541 | **783.5087** | 392.2580 | **7** |
| **11** | **1284.7634** | 642.8853 | **T** | 685.4355 | 343.2214 | 669.4168 | 335.2120 | 670.4246 | 335.7160 | **6** |
| **12** | 1412.8584 | 706.9328 | **K** | 584.3879 | 292.6976 | 568.3691 | 284.6882 | 569.3770 | 285.1921 | **5** |
| **13** | 1509.9111 | 755.4592 | **P** | 456.2929 | 228.6501 | 440.2742 | 220.6407 | 441.2820 | 221.1446 | **4** |
| **14** | **1580.9483** | 790.9778 | **A** | 359.2401 | 180.1237 | **343.2214** | 172.1143 | **344.2292** | 172.6183 | **3** |
| **15** | **1694.0323** | **847.5198** | **I** | 288.2030 | 144.6051 | **272.1843** | 136.5958 | **273.1921** | 137.0997 | **2** |
| **16** |  |  | **R** | 175.1190 | 88.0631 | 159.1002 | 80.0538 | 160.1081 | 80.5577 | **1** |
