# Supplementary material for: Histone Variants and Their Post-Translational Modifications in Primary Human Fat Cells
Source: PLoS One. 2011 Jan 7;6(1):e15960. doi: 10.1371/journal.pone.0015960 (PMC3017551; doi:10.1371/journal.pone.0015960)
Supplement: Figure S5 — Example of not accepted peptide identification from MASCOT data analysis of the modified N-terminal peptide (amino acids 3-18) from histone H3. The spectrum, corresponding list of singly and doubly charged fragment ions and positions of six modified residues identified in the MASCOT search are shown. The ion fragmentation spectrum has a low MASCOT score, a high E-value and not considered as the peptide identification, although it was found in several LC/MS/MS runs from the same subject. (DOC) [file pone.0015960.s005.doc]

**Figure S5. Example of not accepted peptide identification from MASCOT data analysis of the modified N-terminal peptide (amino acids 3-18) from histone H3.**

**H3 histone, family 3A,** GI: 51859376

MS/MS Fragmentation of **TKQTALKSTGGKAPR, 623,6^3+^**


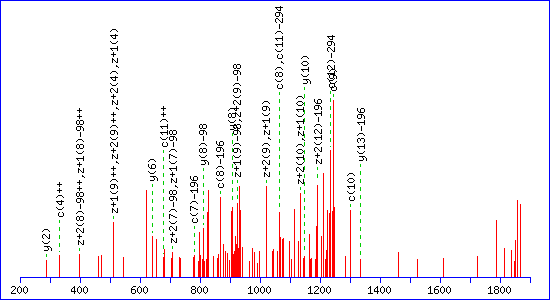


**T1 :** Phospho (ST), with neutral losses 97.9769

**K2 :** Dimethyl (K)

**T4 :** Phospho (ST), with neutral losses 97.9769

**T9 :** Phospho (ST), with neutral losses 97.9769

**K12 :** Acetyl (K)

**R15 :** Methyl (R)

**Ions Score:** 12 **Expect:** 18

**Matches (Red) :** 30/188 fragment ions using 62 most intense peaks

| **#** | **c** | **c^++^** | **Seq.** | **y** | **y^++^** | **z+1** | **z+1^++^** | **z+2** | **z+2^++^** | **#** |
| --- | --- | --- | --- | --- | --- | --- | --- | --- | --- | --- |
| **1** | 101.0709 | 51.0391 | **T** |  |  |  |  |  |  | **15** |
| **2** | 257.1972 | 129.1022 | **K** | 1490.8802 | 745.9437 | 1474.8614 | 737.9344 | 1475.8693 | 738.4383 | **14** |
| **3** | 385.2558 | 193.1315 | **Q** | **1334.7539** | 667.8806 | 1318.7352 | 659.8712 | 1319.7430 | 660.3751 | **13** |
| **4** | 468.2929 | 234.6501 | **T** | 1206.6953 | 603.8513 | 1190.6766 | 595.8419 | **1191.6844** | 596.3459 | **12** |
| **5** | 539.3300 | 270.1686 | **A** | 1123.6582 | 562.3327 | 1107.6395 | 554.3234 | 1108.6473 | 554.8273 | **11** |
| **6** | 652.4141 | 326.7107 | **L** | 1052.6211 | 526.8142 | 1036.6024 | 518.8048 | 1037.6102 | 519.3087 | **10** |
| **7** | **780.5090** | 390.7582 | **K** | 939.5370 | 470.2722 | 923.5183 | 462.2628 | **924.5261** | 462.7667 | **9** |
| **8** | **867.5411** | 434.2742 | **S** | **811.4421** | 406.2247 | 795.4234 | 398.2153 | 796.4312 | 398.7192 | **8** |
| **9** | 950.5782 | 475.7927 | **T** | 724.4101 | 362.7087 | 708.3913 | 354.6993 | **709.3992** | 355.2032 | **7** |
| **10** | 1007.5996 | 504.3035 | **G** | **641.3729** | 321.1901 | 625.3542 | 313.1807 | 626.3620 | 313.6847 | **6** |
| **11** | **1064.6211** | 532.8142 | **G** | 584.3515 | 292.6794 | 568.3327 | 284.6700 | 569.3406 | 285.1739 | **5** |
| **12** | **1234.7266** | 617.8670 | **K** | 527.3300 | 264.1686 | 511.3113 | 256.1593 | **512.3191** | 256.6632 | **4** |
| **13** | 1305.7637 | 653.3855 | **A** | 357.2245 | 179.1159 | 341.2058 | 171.1065 | 342.2136 | 171.6104 | **3** |
| **14** | 1402.8165 | 701.9119 | **P** | **286.1874** | 143.5973 | 270.1686 | 135.5880 | 271.1765 | 136.0919 | **2** |
| **15** |  |  | **R** | 189.1346 | 95.0709 | 173.1159 | 87.0616 | 174.1237 | 87.5655 | **1** |
