# Supplementary material for: Histone Variants and Their Post-Translational Modifications in Primary Human Fat Cells
Source: PLoS One. 2011 Jan 7;6(1):e15960. doi: 10.1371/journal.pone.0015960 (PMC3017551; doi:10.1371/journal.pone.0015960)
Supplement: Table S1 — Characteristics of participating subjects. (PDF) [file pone.0015960.s006.pdf]

Table S1. Characteristics of participating subjects.
All subjects were female and nobody was diagnosed with diabetes

Subject	Age (years)	BMI (kg/m^2)
1	50	27.0
2	68	24.5
3	76	31.6
4	35	24.4
5	68	32.2
6	72	27.3
