# Supplementary material for: Histone Variants and Their Post-Translational Modifications in Primary Human Fat Cells
Source: PLoS One. 2011 Jan 7;6(1):e15960. doi: 10.1371/journal.pone.0015960 (PMC3017551; doi:10.1371/journal.pone.0015960)
Supplement: Table S2 — Proteins from acid extracted whole cells identified by the LC/MS/MS. (PDF) [file pone.0015960.s007.pdf]

Table S2. Proteins from acid extracted whole cells identified by the LC/MS/MS

Protein	NCBI acc. number	MW (kDa)	pI	location[1]	peptide#[2]
Adipose specific 2	GI:5802976	7.9	5.2		4
Annexin A1	GI:4502101	38.7	6.6	Cyt,Nuc	5
Annexin A2	GI:18645167	38.6	7.6	End	5
Calmodulin	GI:825635	17.2	4.1	Cyt,Nuc	5
chaperonin 10	GI:4008131	10.6	9.4	Mt	3
diazepam binding inhibitor	GI:10140853	11.8	5.4		3
dismutase,Cu/Zn superoxide	GI:4507149	15.9	5.7	Cyt	3
elongation factor 1-alpha	GI:62896597	39.3	9	Cyt	2
fatty acid binding protein 4	GI:4557579	14.7	6.6	Cyt,Nuc	2
fatty acid binding protein 5	GI:4557581	15.2	6.6	Cyt	2
ferritin, heavy polypeptide 1	GI:56682959	21.2	5.3		4
Gamma-synuclein	GI:3347842	13.3	5	Cyt	8
Alpha 2 globin	GI:4504345	15.3	8.7	Rbc	5
Beta globin	GI:71727231	16	7.9	Rbc	9
Histone H1	different isof.	21	11	Nuc	7
Histone H2A	different isof.	14	11	Nuc	10
Histone H2B	different isof.	14	10.3	Nuc	12
Histone H3	different isof.	15	11.3	Nuc	6
Histone H4	different isof.	11	11	Nuc	7
Perilipin	GI:21410323	55.9	5.8	LD	3
plasma membrane associated protein, S3-12	GI:122937195	134.4	8.9	Mem,Cyt,LD	30
PTRF	GI:42734430	43.5	5.5	Nuc,Mem	5
ribosomal protein L13	GI:15431295	24.0	11.6	Rb	2
ribosomal protein P2	GI:4506671	12.0	4.4	Rb	3
S100 calcium binding protein A6	GI:7657532	10.2	5.3	Cyt,NucE	2
S100 calcium binding protein A10	GI:4506761	11.2	6.8	Mt	4
S100 calcium binding protein A11	GI:5032057	11.7	6.6	Cyt,Nuc	3
serum albumin	GI:28592	69.4	6.1	Sc	12
tropomyosin 1	GI:63252898	32.7	4.7	Cyt	2
tropomyosin 4	GI:4507651	28.5	4.7	Cyt	7
Vimentin (desmin)	GI:62414289	53.7	5.1	Cyt	27

1: as in table 1 *2:* peptides are identified with both CID and ETD or have a MASCOT score over 45
